# Honeysuckle‐Derived Nanovesicles Regulate Gut Microbiota for the Treatment of Inflammatory Bowel Disease

**DOI:** 10.1002/advs.202505208

**Published:** 2025-09-19

**Authors:** Yuanyuan Wang, Yuanhao Zhou, Qingyuan Wu, Yishu Li, Yilin Huang, Kexin Yu, Ping Li, Zhenye Lv, Haotian Liu, Hai Zou, Huiyu Liu, Xiaozhou Mou

**Affiliations:** ^1^ Center for Rehabilitation Medicine Rehabilitation and Sports Medicine Research Institute of Zhejiang Province Department of Rehabilitation Medicine Clinical Research Institute Zhejiang Provincial People's Hospital, Affiliated People's Hospital Hangzhou Medical College Hangzhou 310014 P. R. China; ^2^ Beijing Advanced Innovation Center for Soft Matter Science and Engineering State Key Laboratory of Organic‐Inorganic Composites Beijing Laboratory of Biomedical Materials Bionanomaterials & Translational Engineering Laboratory Beijing Key Laboratory of Bioprocess Beijing University of Chemical Technology Beijing 100029 P. R. China; ^3^ Department of Critical Care Shanghai Cancer Center Fudan University Shanghai 200032 China

**Keywords:** inflammatory bowel disease, honeysuckle‐derived nanovesicles, gut microbiota, metabolite, short chain fatty acids, secondary bile acid

## Abstract

Inflammatory bowel disease (IBD), which includes ulcerative colitis (UC) and Crohn's disease, significantly impairs patients’ quality of life and is characterized by a compromised intestinal barrier, pathogenic bacterial colonization, and chronic inflammation. The gut microbiota has emerged as a therapeutic target for IBD owing to its role in modulating immune responses, metabolic pathways, and barrier function. In this study, the therapeutic potential of honeysuckle‐derived nanovesicles (HNVs) in a dextran sodium sulfate‐induced murine model of UC is investigated. Oral administration of HNVs alleviates colitis symptoms, reduces colonic inflammation and histopathological damage, restores Treg/Th17 balance, and repairs intestinal barrier integrity. These effects are associated with favorable alterations in the gut microbiota, including an increase in beneficial bacteria and a decrease in pathogenic species, along with elevated levels of short‐chain fatty acids (SCFAs), which are essential for maintaining mucosal immunity and barrier function. Moreover, metabolomic analysis reveals that HNVs promoted bile acid (BA) absorption and regulated BA metabolism, contributing to the maintenance of intestinal immune homeostasis. Collectively, these findings demonstrate the protective effects of HNVs through modulation of SCFA levels and BA metabolism, supporting their potential as a promising therapeutic strategy for the treatment of IBD.

## Graphical abstract

This study explores honeysuckle‐derived nanovesicles (HNVs) for treating inflammatory bowel disease. Orally administered HNVs alleviates colitis in mice by repairing the gut barrier, reducing inflammation, and restoring a healthy gut microbiota. This is linked to increased beneficial short‐chain fatty acids and regulated bile acid metabolism, highlighting HNVs' therapeutic potential.

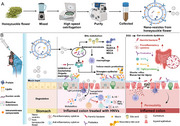

## 1 Introduction

Inflammatory bowel disease (IBD), comprising both Crohn's disease and ulcerative colitis (UC), is a chronic, nonspecific inflammatory gastrointestinal disorder that has emerged as a growing global healthcare burden.^[^ ^1^ ^]^ By 2017, the prevalence of IBD had escalated to 6.8 million patients worldwide, seriously impacting quality of life and increasing the risk of colorectal cancer.^[^ ^2^ ^]^ With a large number of high‐risk individuals, the absolute number of IBD patients in emerging industrialized countries such as China and India may approach that of Western nations by 2025.^[^ ^3^ ^]^ The pathogenesis of IBD is generally attributed to the intricate interplay between genetic predisposition and environmental factors, which collectively influence the composition and function of the gut microbiota. This imbalance compromises intestinal barrier integrity and subsequently leads to immune dysregulation, including excessive activation of Th1/Th17 cells and impairment of Foxp3⁺ regulatory T (Treg) cells.^[^ ^4^, ^5^ ^]^

In healthy individuals, the gut microbiota maintains homeostasis through its roles in intestinal metabolism, preservation of mucosal integrity, clearance of pathogens, and modulation of the host immune system.^[^ ^6^ ^]^ The gut microbiome is primarily composed of Firmicutes, Bacteroidetes, Proteobacteria, and Actinobacteria, with Firmicutes and Bacteroidetes accounting for nearly 90% of the total microbiota.^[^ ^7^ ^]^ However, patients with IBD exhibit gut microbial dysbiosis, characterized by reduced microbial diversity and decreased abundance of Bacteroidetes and Firmicutes.^[^ ^8^ ^]^

Metabolites produced by the gut microbiota serve as crucial molecular mediators that facilitate interactions between the microbiome and the host.^[^ ^9^ ^]^ Notably, metabolites derived from tryptophan, short‐chain fatty acids (SCFAs), and bile acids (BAs) have been identified as key modulators of the intestinal immune response.^[^ ^10^ ^]^ Alterations in gut microbial composition lead to changes in metabolite profiles, which play a significant role in the pathogenesis of IBD.^[^ ^11^ ^]^ Studies have revealed marked differences in gut metabolites between patients with IBD and healthy individuals, particularly in BAs, sphingolipids (SPHs), and cholesterol.^[^ ^12^ ^]^ The complex pathology of IBD is characterized by excessive apoptosis of intestinal epithelial cells (IECs), gut microbiota dysbiosis, intestinal barrier damage, heightened inflammatory responses, and immune dysfunction.^[^ ^13^, ^14^, ^15^ ^]^

Current clinical treatments primarily include anti‐inflammatory small‐molecule drugs, immunosuppressants, and biologics such as infliximab and adalimumab.^[^ ^16^ ^]^ However, the therapeutic efficacy of these interventions remains limited, and they are often associated with significant complications, the development of drug tolerance, and long‐term immune‐related adverse events. Consequently, there is an urgent need for alternative therapies that offer high efficacy with minimal side effects for patients with IBD.

Recently discovered plant‐derived exosome‐like nanovesicles (PNVs) are nanoscale vesicles with a bilayer membrane structure that play crucial roles in intercellular communication, signal transduction, and the maintenance of organismal homeostasis. PNVs are rich in bioactive molecules such as nucleic acids, proteins, and lipids, and exhibit immunomodulatory, antitumor, regenerative, and anti‐inflammatory effects.^[^ ^17^, ^18^, ^19^, ^20^ ^]^ PNVs from various sources, including grapes, grapefruit, broccoli, ginger, sweet oranges, mulberry bark, and tea, have demonstrated potential in the prevention and treatment of IBD.^[^ ^21^, ^22^, ^23^, ^24^, ^25^, ^26^ ^]^

Our current study showed that honeysuckle‐derived exosome‐like nanovesicles (HNVs) effectively alleviated weight loss in dextran sodium sulfate (DSS)‐induced mouse colitis models. Honeysuckle is a medicinal and edible plant known for its heat‐clearing, detoxifying, and anti‐inflammatory properties. It is also the main ingredient of the Chinese recipe *Peach Blossom Soup*, a well‐known herbal prescription for the treatment of IBD and UC in China.^[^ ^27^ ^]^ Modern pharmacological studies have confirmed its antibacterial, antiviral, anti‐inflammatory, antipyretic, and immune‐enhancing effects.^[^ ^28^, ^29^, ^30^, ^31^, ^32^ ^]^ However, the specific mechanisms through which HNVs alleviate IBD symptoms remain unclear.

In this study, we investigated the protective effects of HNVs on intestinal barrier integrity, as well as their therapeutic potential in DSS‐induced mouse colitis models. Our findings revealed that HNVs exert protective effects on gut barrier integrity at multiple levels, including the chemical, physical, and immune barriers, and demonstrated substantial therapeutic efficacy. The mechanisms of action include: (a) modification of the gut microbiome; (b) alteration of gut microbial metabolites; and (c) protection of the intestinal barrier through microbe–host interactions, reduction of inflammation in the blood and colon, and rebalancing of the Treg/Th17 response. These results suggest that HNVs maintain gut mucosal repair and immune homeostasis through a multifactorial approach.

## 2 Results

### 2.1 Isolation and Characterization of HNVs

**Figure 1:**
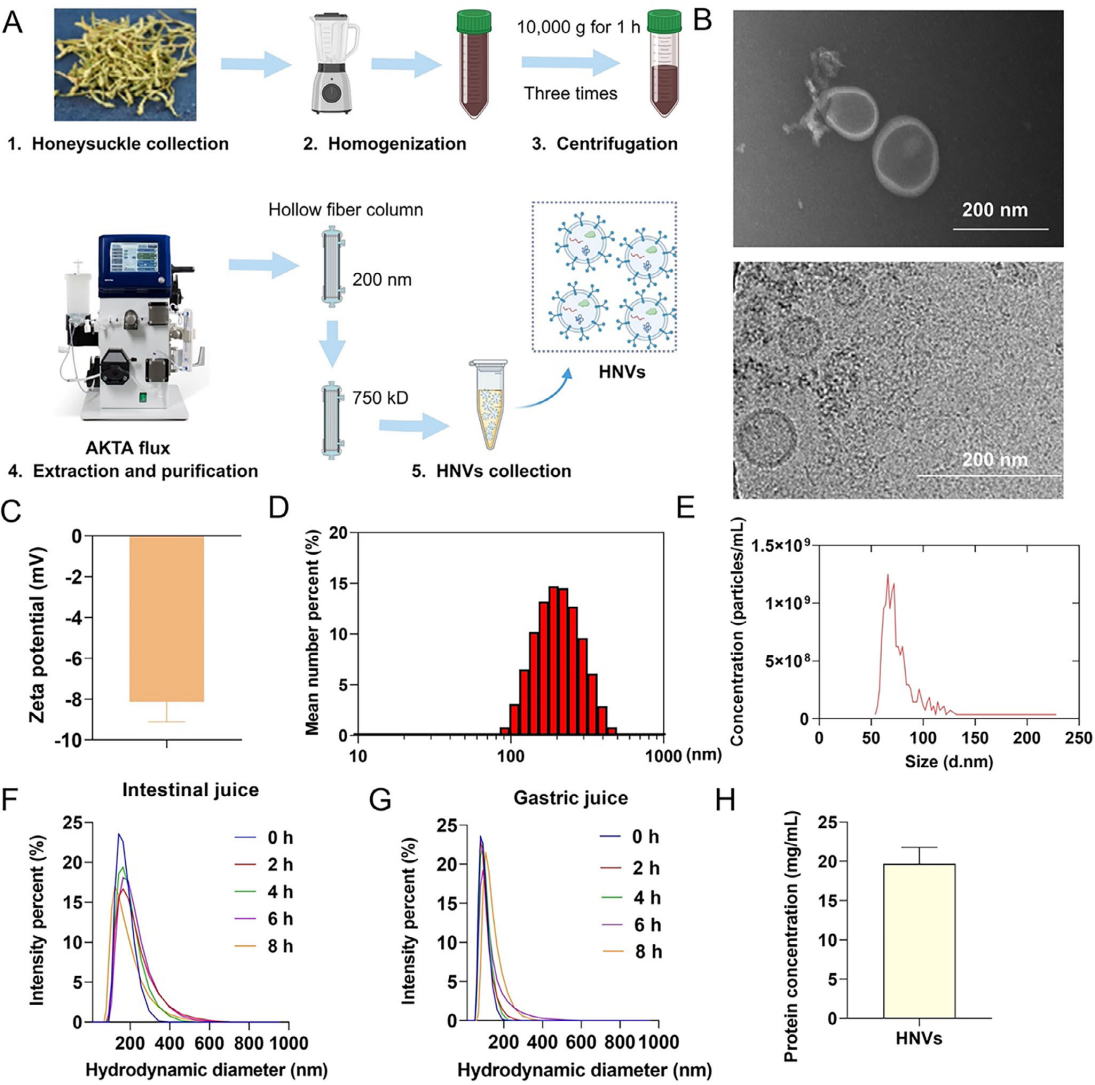
Identification and characterization of honeysuckle exosome‐like vesicles (HNVs). A) HNVs were isolated and purified following a detailed and standardized protocol. B) TEM (upper panel) and Cryo‐TEM (lower panel) imaging of HNVs. C–D) DLS analysis showing C) zeta potential and D) size distributions of HNVs. E) Counting of HNVs particles by nanoparticle tracking analysis (NTA). F–G) Changes in size distribution of HNVs after incubation with F) gastric juice and G) intestinal juice at 37 °C. H) Quantification of HNVs protein concentration by Bradford assay. Scale bar = 200 nm.

Honeysuckle juice (HJ) was obtained from the homogenate via centrifugation, followed by the isolation of HNVs using AKTA tangential flow filtration (**Figure** **1**A). The morphology of HNVs was characterized using transmission electron microscopy (TEM) and cryo‐TEM, which revealed that the HNVs were uniformly oval‐shaped vesicles with an approximate diameter of 100 nm (Figure 1B). The size, diameter, and zeta potential of the HNVs were further analyzed by dynamic light scattering (DLS). The HNVs exhibited a negative zeta potential of −8.13 mV and an average size of 196 nm (Figure 1C,D). Additionally, nanoparticle tracking analysis indicated that the majority of HNVs were ≈77 ± 19.6 nm in diameter, and the particle concentration was found to be 1.48 × 10^12^ particles/mL (Figure 1E). Stability assessments in simulated small intestinal fluid (Figure 1F) and simulated gastric fluid (Figure 1G) at 37 °C indicated that the HNVs remained stable for at least 8 h.

Quantification of HNVs was performed by measuring protein concentration using the Bicinchoninic Acid, which determined a concentration of 19.67 mg/mL of fresh HNVs, with 1 mL being obtained from 2 g of dried honeysuckle (Figure 1H). The yield and number of HNVs per gram of honeysuckle tissue, and the yield of HNVs‐RNA per milligram of HNVs, were also quantitatively analyzed (Figure S1A–C, Supporting Information). The purity of the HNVs preparations was determined by comparing the ratio of HNVs counts to protein concentration (Figure S1D, Supporting Information).

In this study, in addition to TEM and cryo‐TEM imaging, HNVs were characterized based on their protein content (Figure S2A,B, Supporting Information) and RNA profiles (Figure S2C, Supporting Information). Nontargeted lipidomics was used to analyze the lipid composition of HNVs. The results showed that the lipid components of HNVs were mainly triglycerides (63.81%), diglycerides (20.87%), ceramides (4.77%), monoglycerides (3.61%), SPHs (1.52%), and cardiolipins (0.89%) (Figure S2D, Supporting Information). The number of double bonds per lipid carbon chain was mainly two (20.70%), three (17.68%), four (17.17%), and one (11.03%) (Figure S2E, Supporting Information). The chain length of the lipid carbon atoms was predominantly 36 carbons (21.54%), 54 carbons (11.80%), 38 carbons (8.36%), 57 carbons (5.87%), and 52 carbons (5.16%) (Figure S2F, Supporting Information).

Furthermore, nontargeted metabolomics analysis was performed to identify compounds present in HNVs. The results indicated that HNVs primarily contained organooxygen compounds, carboxylic acids and derivatives, fatty acyls, prenol lipids, and flavonoids (Figure S2G, Supporting Information). Next‐generation sequencing analysis of HNVs‐RNA further revealed that HNVs contained 2932 miRNAs. The most abundant HNVs miRNAs included PC‐5p‐23_116203 (37.56%), PC‐5p‐39_54233 (22.99%), PC‐3p‐33_68273 (8.94%), PC‐3p‐119_17928 (4.25%), ptc‐miR6478_R‐1 (1.07%), and ath‐miR319a_L‐1R‐1 (1.06%) (Figure S2H, Supporting Information).

In addition, the double‐layer membrane structure of exosome‐like nanovesicles from honeysuckle decoction (HD) was severely damaged (Figure S3A, Supporting Information). These vesicles exhibited a negative zeta potential of −7.29 mV (Figure S3B, Supporting Information), and the particle concentration was found to be 2.87 × 10^10^ particles/mL, which is significantly lower than that of the HJ extract, only 1% of its concentration (Figure S3C, Supporting Information).

### 2.2 Therapeutic Efficacy of HNVs in DSS‐Induced IBD Mouse Model

**Figure 2:**
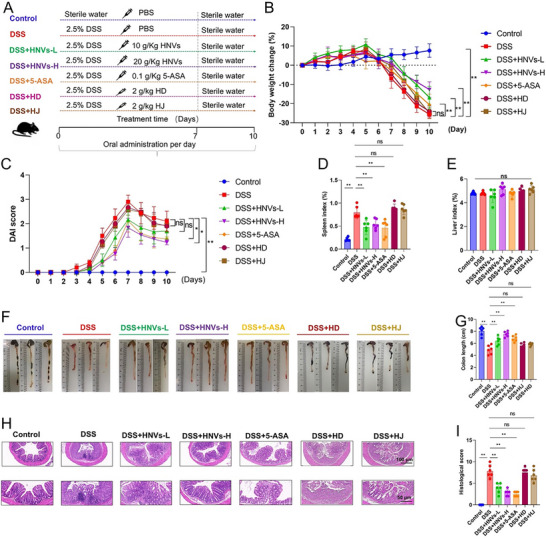
Effects of HNVs treatment on DSS‐induced experimental IBD symptoms in C57BL/6 mice. Schematic illustration of the DSS‐induced IBD mouse model. (B) Body weight changes over 10 days. C) Disease activity index (DAI) scores over 10 days. D–E) Organ indices for the spleen (D) and liver (E). F) Representative colon photographs and G) colon length measurements. H) H&E staining of colonic tissues. I) Histopathological scores based on H&E staining analysis. Data are presented as mean ± SD (*n* = 6). *P*‐values were determined using one‐way ANOVA. *P < 0.05, **P < 0.01, ns: not significant. HNVs‐H: high‐dose treatment group; HNVs‐L: low‐dose treatment group. Scale bar = 50 or 100 µm.

The experimental design and corresponding flowchart are presented in **Figure** **2**A. Compared with the DSS‐only group, both the HNVs and 5‐aminosalicylic acid (5‐ASA) treatment groups exhibited reduced body weight loss (Figure 2B), lower disease activity index (DAI) scores (Figure 2C), and decreased spleen indices (Figure 2D), while liver indices showed no significant differences (Figure 2E). Furthermore, these groups demonstrated increased colon lengths (Figure 2F,G), normal colonoscopic appearances (Figure S4A, Supporting Information), and improved histological architecture with less inflammatory cell infiltration (Figure 2H), resulting in significantly lower histological scores (Figure 2I) and reduced intestinal permeability (Figure S4B¸ Supporting Information).

Notably, the therapeutic differences between the high‐dose treatment group receiving 20 g kg^−1^ of HNVs (DSS + HNVs‐H) and the healthy control group were less pronounced than those between the DSS + 5‐ASA group and the healthy control, suggesting that HNVs may provide enhanced therapeutic benefits in treating DSS‐induced colitis. It is important to note that neither the HD nor HJ treatment groups showed significant therapeutic effects. This may be due to the lower concentration of exosome‐like nanovesicles in the HD and HJ stock solutions. HNVs contain abundant miRNAs and other bioactive components and demonstrate high bioavailability, which may account for their strong therapeutic efficacy. Furthermore, the high‐dose treatment group (20 g kg^−1^) showed greater therapeutic efficacy compared to the low‐dose treatment group (10 g kg^−1^, DSS + HNVs‐L).

### 2.3 HNVs Attenuate Mucosal Immune Infiltration and Inflammation in DSS‐Induced Mice

**Figure 3:**
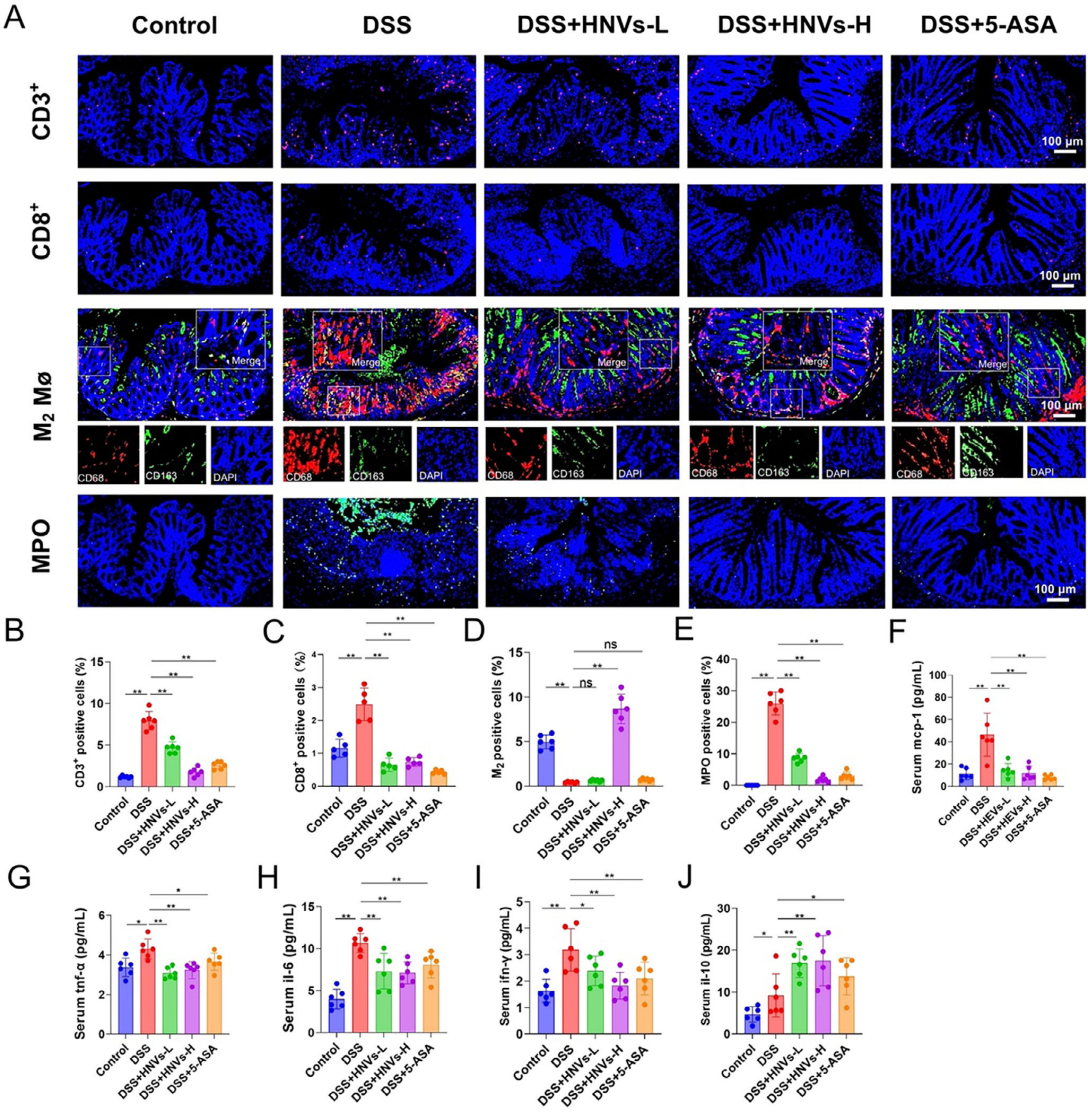
Effects of HNVs treatment on the inflammatory response in DSS‐induced experimental IBD in C57BL/6 mice. A) Immunofluorescence staining of colonic tissues showing CD3⁺, CD8⁺, M2 macrophage (Mø), and MPO‐positive cells. B–E) Quantification of CD3⁺, CD8⁺, M2 Mø, and MPO‐positive cells in colonic tissues of each group using ImageJ software. (F–J) ELISA‐based quantification of MCP‐1, TNF‐α, IL‐6, IFN‐γ, and IL‐10 protein levels in serum from each group. Data are represented as mean ± SD (*n* = 6). *P*‐values were determined using one‐way ANOVA. *P < 0.05, **P < 0.01, ns: not significant. HNVs‐H: high‐dose treatment group; HNVs‐L: low‐dose treatment group. Scale bar = 100 µm.

In patients with IBD, high levels of immune cell infiltration in the mucosa are associated with inflammatory activity.^[^ ^33^ ^]^ Therefore, we examined the distribution of immune infiltrates in colon tissues among the five groups (**Figure** **3**A). The DSS group exhibited significantly more CD3⁺ T cells, CD8⁺ T cells, and myeloperoxidase (MPO)‐positive cells in the mucosa compared to the HNVs or 5‐ASA treatment groups. Additionally, the number of M2 macrophage (Mø)‐positive cells was markedly lower in the DSS group than in the DSS + HNVs‐H group (Figure 3B–E).

Furthermore, both the HNVs and 5‐ASA treatment groups showed significant reductions in pro‐inflammatory cytokines, including MCP‐1, TNF‐α, IL‐6, and IFN‐γ, accompanied by an increase in the anti‐inflammatory cytokine IL‐10, compared with the DSS group (Figure 3F–J).

The therapeutic efficacy of HNVs is primarily attributed to their capacity to selectively target inflamed lesions while minimizing adverse effects on other organs. In vivo cytotoxicity was evaluated in healthy C57BL/6 mice following oral administration of HNVs at doses of 10 and 20 g kg^−1^ daily for 8 consecutive days. During treatment, no significant differences in body weight were observed between the HNVs‐treated groups and the PBS control group (Figure S5A, Supporting Information). At the end of the study, mice were sacrificed for routine blood tests. Biochemical markers including blood urea nitrogen, creatinine, alanine aminotransferase, and aspartate aminotransferase remained within normal physiological ranges, with no significant differences between the HNVs‐treated and control groups, confirming the absence of hepatic or renal toxicity (Figure S5B, Supporting Information).

Histopathological evaluation by H&E staining of major organs, including the heart, liver, spleen, lungs, kidneys, and colon, revealed no notable pathological changes or inflammatory lesions in the HNVs‐treated mice compared to the healthy control group (Figure S5C, Supporting Information). These results emphasize the excellent safety profile of HNVs for biomedical applications.

The biodistribution of HNVs was further investigated in a mouse model of IBD. HNVs were conjugated with the fluorescent dye Cy5.5 to track their in vivo localization after oral administration. In IBD mice, HNVs accumulated significantly in inflamed colonic lesions and in major organs such as the liver and kidneys. In contrast, healthy control mice showed no notable HNVs accumulation in these organs. The distribution of HNVs in the colon was markedly lower in healthy mice than in IBD mice following oral administration. Additionally, HNVs persisted in colonic lesions of IBD mice for over 24 h.

Immunofluorescence staining further demonstrated that HNVs crossed the intestinal epithelium and reached the serosa, where they were taken up by dendritic cells (CD11c⁺) and macrophages (CD68⁺) in IBD mice (Figure S6, Supporting Information). This phenomenon was not observed in normal healthy mice (Figure S7, Supporting Information). Subsequently, co‐incubation of HNVs with RAW264.7 cells showed time‐dependent uptake, as observed by confocal laser scanning microscopy (CLSM). The nuclear morphology and structure of RAW264.7 cells remained normal and well‐defined. After 3 h of incubation, strong green fluorescence was observed, indicating significant HNVs uptake by the cells (Figure S8, Supporting Information).

### 2.4 HNVs Protect Colonic Mucosal Barrier Function in DSS‐Induced Mice

**Figure 4:**
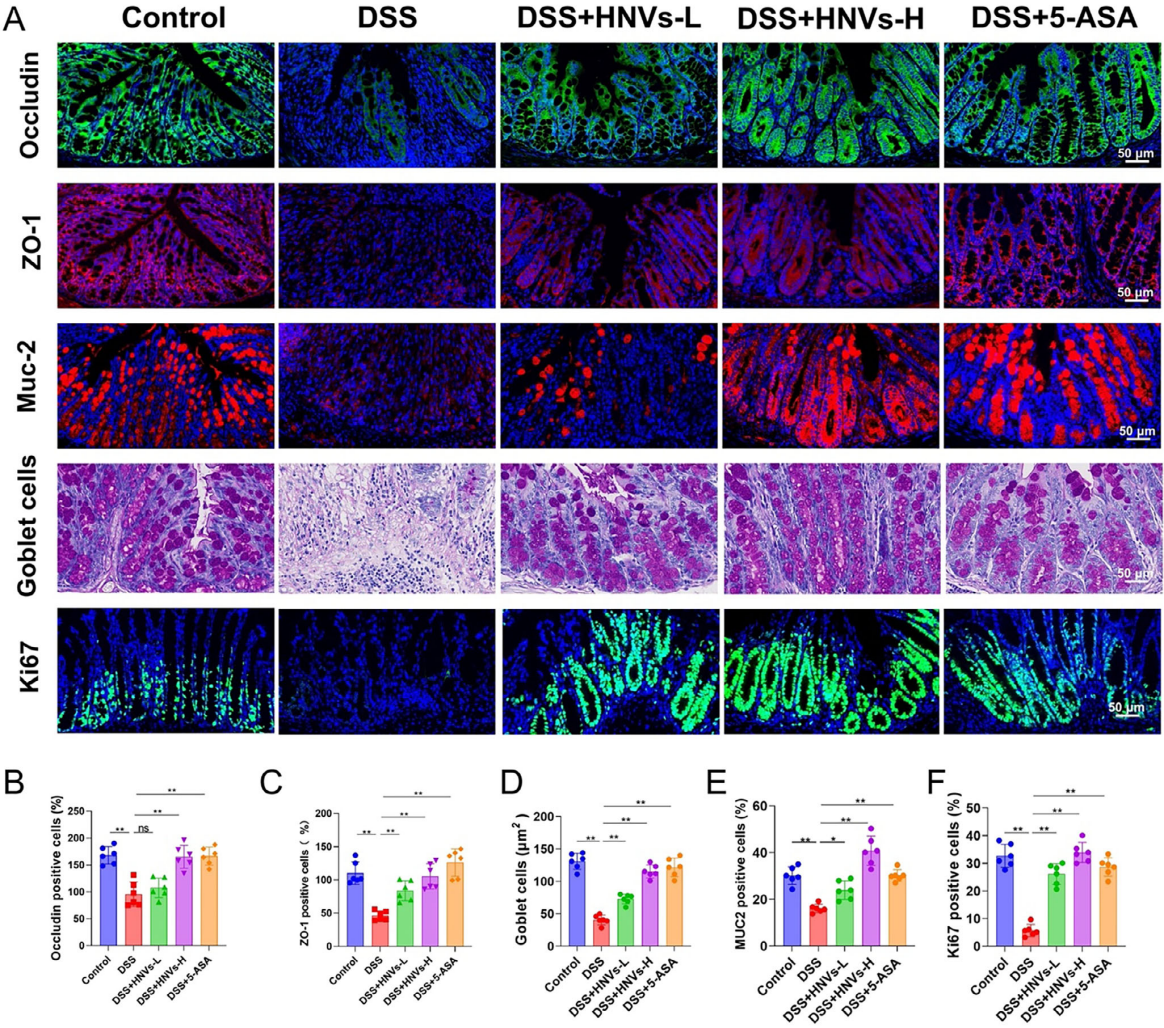
Effects of HNVs treatment on the colonic tissue mucosal barrier in DSS‐induced experimental IBD in C57BL/6 mice. From top to bottom: A) Colonic tissue fluorescence intensity of Occludin, ZO‐1, and Muc2; number of goblet cells based on PAS staining; and cellular proliferation assessed by Ki67 staining. B–F) Quantification of Occludin‐, ZO‐1‐, Muc2‐, and Ki67‐positive cells in colonic tissues of mice in each group using Image J software, and quantification of goblet cell numbers using Image Pro Plus software. Data are presented as mean ± SD (*n* = 6). *P*‐values were determined using one‐way ANOVA. *P < 0.05, **P < 0.01, ns: not significant. HNVs‐H: high‐dose treatment group; HNVs‐L: low‐dose treatment group. Scale bar = 50 µm.

In patients with IBD, the expression and function of tight junction proteins are impaired, leading to decreased intestinal barrier function. As a result, harmful substances can easily penetrate the intestinal mucosa and trigger inflammatory responses.^[^ ^34^ ^]^ Therefore, we examined the expression of tight junction proteins in colonic tissues across the five groups (**Figure** **4**A).

HNVs treatment contributed to the preservation of colonic mucosal barrier integrity, as evidenced by increased expression of Occludin‐ and ZO‐1‐positive cells, a higher number of mucin‐2 (Muc2)‐expressing and mucin‐secreting goblet cells within the colonic epithelium, and an expanded area of Ki67‐positive proliferating cells (Figure 4B–F).

### 2.5 HNVs Regulate Gut Microbes in DSS‐Induced Mice

An expanding body of research highlights the crucial role of the intestinal microbiome in maintaining gut homeostasis by promoting epithelial barrier function and immune tolerance.^[^ ^35^ ^]^ Disruptions in the composition of gut microbiota have been closely associated with the initiation and progression of IBD.^[^ ^36^ ^]^ To evaluate the impact of HNVs treatment on the gut microbiota, cecal contents were collected and subjected to 16S rRNA gene sequencing analysis.

**Figure 5:**
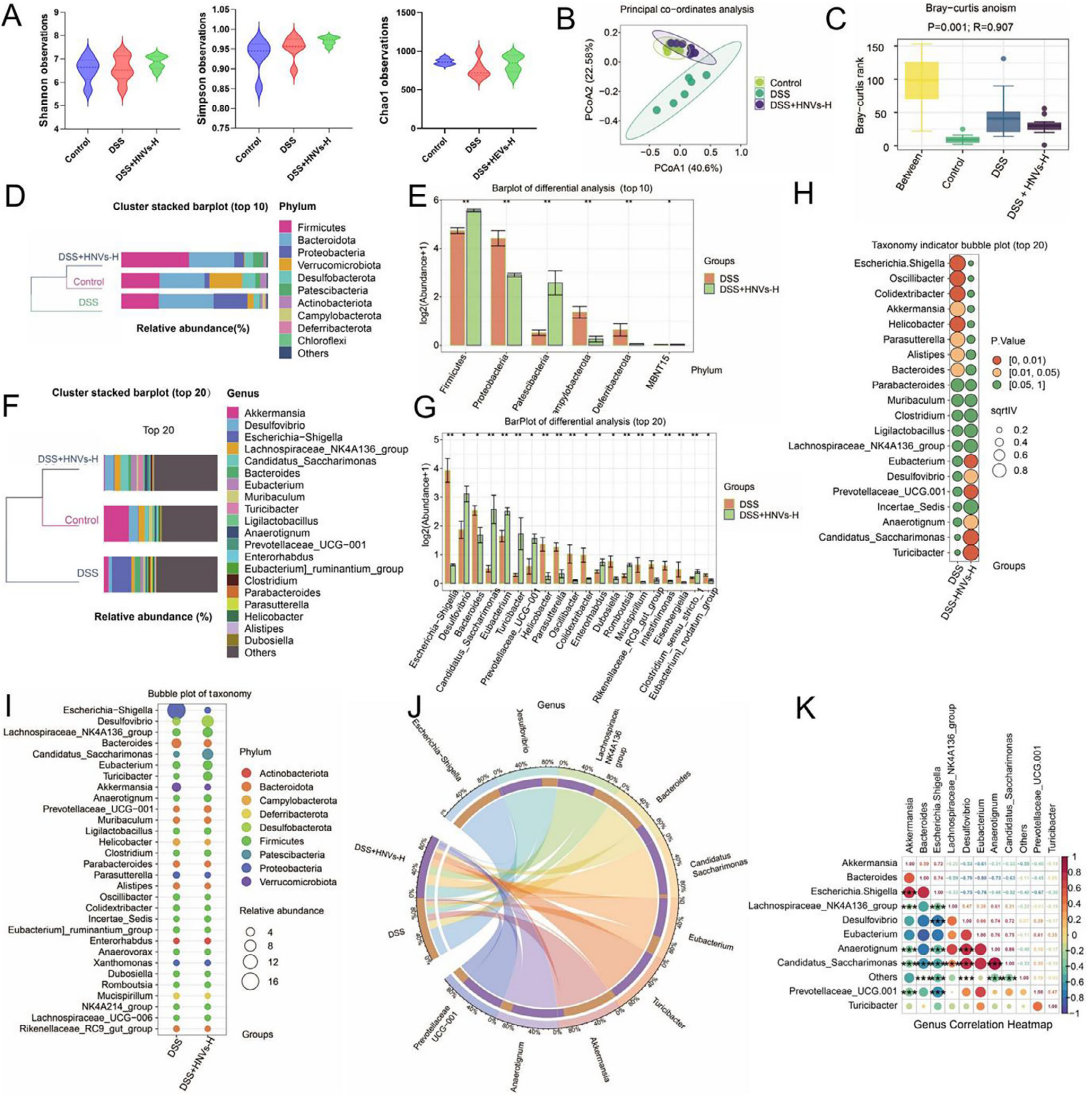
Effects of HNVs treatment on the gut microbiota structure in DSS‐induced experimental IBD in C57BL/6 mice. A) Microbial community α‐diversity was assessed using the Shannon index, Simpson index, and Chao1 index based on observed operational taxonomic units. B) Principal coordinate analysis and C) Bray–Curtis dissimilarity analysis illustrating β‐diversity of the gut microbiome. D) Relative abundance of microbial taxa at the phylum level. E) Significant enrichment of microbial taxa at the phylum level. F) Microbial composition at the genus level. G) Significant enrichment of specific genera. H) Biomarker selection: indicator analysis (sqrtIVt value represents the square root of the indicator value; higher values reflect stronger potential as biomarkers for a given treatment group). I) Bubble plot showing species annotation and relative abundance at the genus level (bubble size) across different treatment groups, with phylogenetic phylum‐level information indicated by bubble color. J) Circos plot: the left half depicts the top five phyla and their relative abundances; the right half indicates grouping information, with wider segments representing higher abundance. K) Correlation analysis: heatmap based on Spearman's correlation of differentially abundant genera. Data are presented as mean ± SD (*n* = 6). *P*‐values were determined using one‐way ANOVA. *P < 0.05, **P < 0.01, ns: not significant. HNVs‐H: high‐dose treatment group.

Analysis of alpha diversity, using the Shannon, Simpson, and Chao1 indices, revealed no statistically significant differences among the three experimental groups (**Figure** **5**A). However, principal coordinate analysis (based on weighted UniFrac distances) and analysis of similarities using Bray–Curtis distances indicated that HNVs treatment shifted the gut microbiota composition toward that of the normal control group (Control), forming distinct clusters separate from the DSS group. This suggests that HNVs significantly altered microbial structure compared to DSS‐treated mice (Figure 5B,C).

Next, we assessed the relative abundance of gut microbiota across the three treatment groups. The most abundant taxa—specifically the top 10 at the phylum level and the top 20 at the genus level—are shown in Figure 5D–G. At the phylum level, DSS‐treated mice exhibited reduced levels of Firmicutes and increased levels of Proteobacteria, Campylobacterota, and Deferribacterota. These changes were significantly reversed following HNVs treatment (Figure 5D,E).

At the genus level, the HNVs treatment group in DSS‐induced mice showed significantly increased abundance of *Candidatus_Saccharimonas*, *Eubacterium*, *Turicibacter*, *Prevotellaceae_UCG‐001*, and *Clostridium_sensu_stricto_1*, along with significantly decreased abundance of *Escherichia‐Shigella*, *Akkermansia*, *Helicobacter*, and *Bacteroides* (Figure 5F,G).

To further explore gut microbiota composition and the associations between the DSS and DSS + HNVs‐H groups, biomarker selection indicator analysis, bubble plot visualization, Circos plot representation, and correlation analysis were conducted. In the DSS group, *Escherichia‐Shigella* was identified as the primary indicator bacterium, whereas in the HNVs treatment group, *Turicibacter* was identified as the predominant indicator species (Figure 5H).

Moreover, the DSS group was primarily characterized by the presence of *Escherichia‐Shigella*, *Akkermansia*, and *Bacteroides*, while the dominant taxa in the HNVs treatment group were *Lachnospiraceae_NK4A136_group*, *Candidatus_Saccharimonas*, *Eubacterium*, *Turicibacter*, *Anaerotignum*, and *Prevotellaceae_UCG‐001* (Figure 5I,J). A significant negative correlation was observed between the dominant bacteria in the DSS group and those in the DSS + HNVs‐H group (Figure 5K).

Furthermore, PICRUSt2‐based functional prediction analysis indicated differences in metabolic pathways, including fatty acid metabolism, between the two groups (Figure S9, Supporting Information). These findings suggest that HNVs may exert therapeutic effects on colitis by suppressing the excessive proliferation of *Escherichia‐Shigella* and *Akkermansia*, while enhancing the growth of beneficial bacteria such as *Eubacterium* and *Turicibacter*.

### 2.6 HNVs Influence the Production of Intestinal Metabolites in Mice with DSS‐Induced Colitis

The gut microbiota produces a wide variety of bioactive small‐molecule metabolites that play crucial roles in cell signaling, maintaining mucosal barrier integrity, and modulating immune responses.^[^ ^37^ ^]^ We hypothesized that the protective effects of HNVs may be related to their ability to enhance the production of specific bioactive metabolites by the gut microbiota, particularly SCFAs and BAs. To investigate the protective mechanisms of HNVs, targeted metabolomics analysis was conducted to assess intestinal SCFA and BA concentrations.

**Figure 6:**
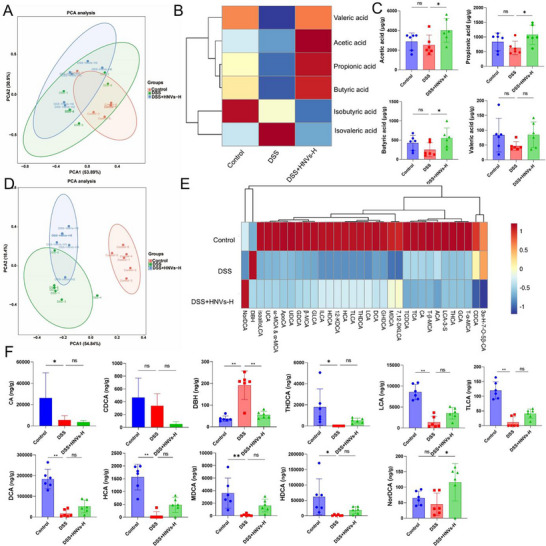
Effects of HNVs treatment on the short‐chain fatty acid (SCFA) and bile acid (BA) concentrations in the cecum contents of DSS‐induced experimental IBD in C57BL/6 mice. A) Principal component analysis (PCA) of SCFAs in cecal contents. B) Cluster analysis of differential SCFAs. C) Bar chart of differential SCFA concentrations. D) PCA of BAs in cecal contents. E) Cluster analysis of differential BAs. F) Bar chart of differential BA concentrations. Data are presented as mean ± SD (*n* = 6), and *P*‐values were determined using one‐way ANOVA. *P < 0.05, **P < 0.01, ns: not significant. HNVs‐H: high‐dose treatment group.

Notably, the cecal contents of the DSS + HNVs‐H group showed significantly elevated levels of acetate and butyrate compared with those of the DSS group (**Figure** **6**A–C). Moreover, HNVs treatment markedly reduced the levels of hydroquinone carboxylic acid (DBH) while increasing the concentrations of 23‐nordeoxycholic acid (NorDCA) in DSS‐treated mice (Figure 6D–F). These findings demonstrate that HNVs significantly modulate the gut microbiota‐related metabolite profile, particularly by enhancing SCFA production.

### 2.7 Transcriptome Analysis of the Therapeutic Mechanism of HNVs in DSS‐Induced Mice

**Figure 7:**
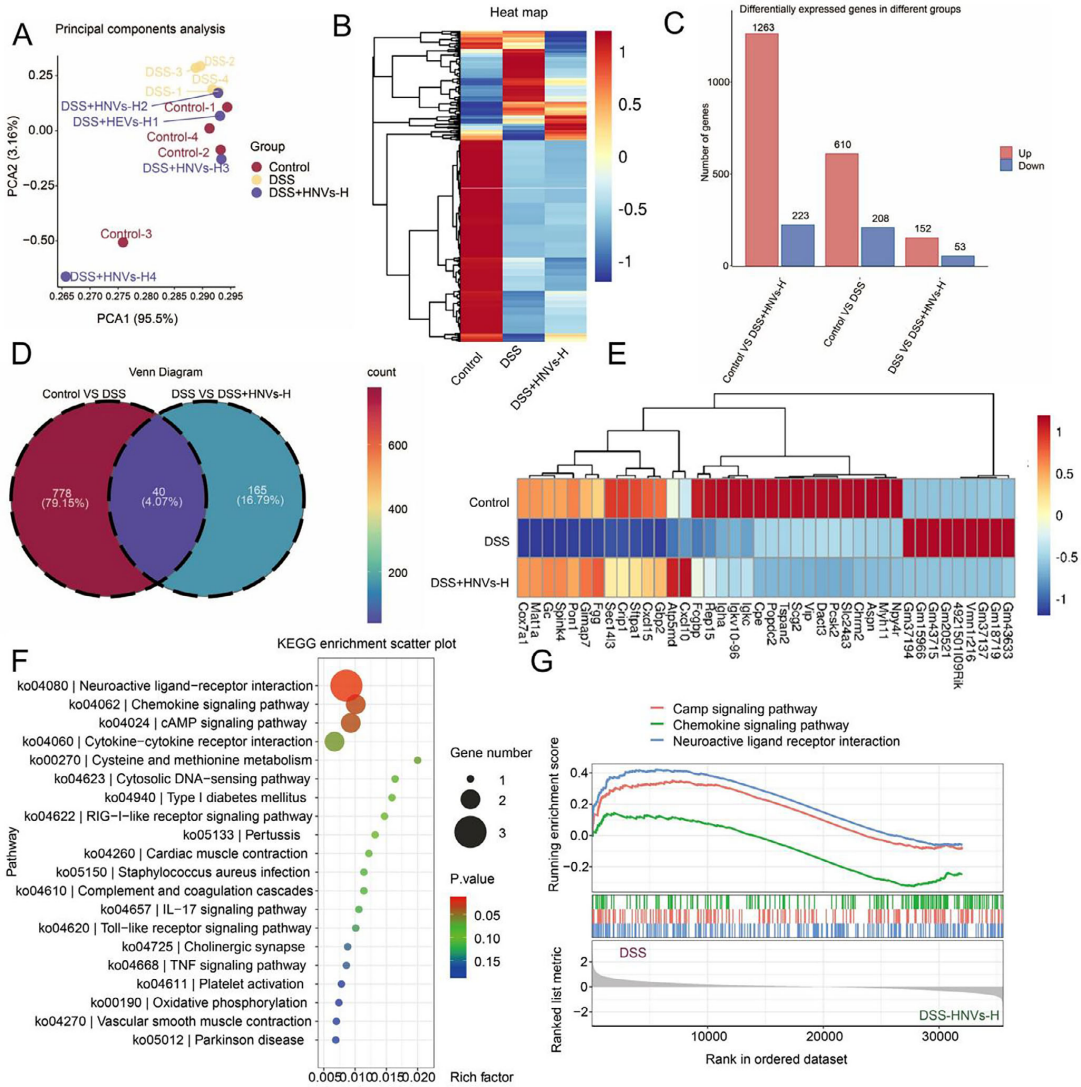
Effects of HNVs treatment on the transcriptome of colonic tissue in DSS‐induced experimental IBD in C57BL/6 mice. A) Principal component analysis (PCA) of gene expression profiles. B) Cluster analysis of differentially expressed genes (DEGs). C) Bar chart showing upregulated and downregulated genes. D) Venn diagram of DEGs in Control versus DSS and DSS versus DSS + HNVs‐H groups. E) Heatmap of 40 overlapping DEGs identified in both comparisons. F) KEGG enrichment bubble chart based on the 40 shared DEGs. G) Gene set enrichment analysis (GSEA) of chemokine and cAMP signaling pathways. HNVs‐H: high‐dose treatment group.

To elucidate the therapeutic mechanisms underlying HNVs in IBD, RNA sequencing (RNA‐seq) was performed on colon tissues collected from mice. Principal component analysis (PCA) revealed distinct clustering of the three experimental groups, Control, DSS, and DSS + HNVs‐H, based on their gene expression profiles (**Figure** **7**A). Heatmap analysis of differentially expressed genes (DEGs) showed significant upregulation and downregulation of genes in the HNVs‐treated mice compared with the DSS group. Additionally, cluster analysis of significantly altered DEGs revealed distinct gene expression profiles across the experimental groups (Figure 7B).

Bar chart analysis showed that, compared with the healthy Control group, the DSS group had 208 upregulated and 610 downregulated genes. In contrast, the DSS + HNVs‐H group exhibited 223 upregulated and 1263 downregulated genes, indicating the impact of inflammatory processes and subsequent therapeutic effects (Figure 7C). Correlation analysis of the DEGs revealed 40 commonly regulated genes between the Control versus DSS and DSS versus DSS + HNVs‐H comparisons, based on a fold change > 2, normalized expression (log_2_) > 1, and q‐value < 0.05 (Figure 7D,E).

Kyoto Encyclopedia of Genes and Genomes (KEGG) pathway enrichment analysis of these DEGs identified several significantly enriched metabolic and signaling pathways, including the neuroactive ligand–receptor interaction pathway, the chemokine signaling pathway, and the cyclic adenosine monophosphate (cAMP) signaling pathway (Figure 7F). To further explore the molecular mechanisms involved, we performed gene set enrichment analysis (GSEA) to evaluate the signaling pathways activated in the DSS and DSS + HNVs‐H groups. As expected, DSS‐treated mice exhibited significant upregulation of the chemokine and cAMP signaling pathways.

The chemokine signaling pathway plays a key role in immune cell recruitment and inflammatory responses, whereas the cAMP signaling pathway is involved in a wide range of physiological processes, including metabolism, secretion, cell proliferation, and differentiation (Figure 7G). These findings suggest that HNVs exert therapeutic effects in part by inhibiting the chemokine signaling pathway, thereby alleviating inflammation, restoring intestinal barrier integrity, and modulating gut microbiota and their associated metabolites.

### 2.8 HNVs Rebalancing CD25^+^Foxp3^+^Treg and IL17^+^CD4^+^T Cell Response in DSS‐Induced Mice

The expression of IL‐17 is significantly increased in the serum and inflamed mucosa of patients with IBD, and IL‐17 produced by Th17 cells plays a critical role in chronic intestinal inflammation⁵. IL‐17–driven inflammation is normally regulated by Tregs and anti‐inflammatory cytokines such as IL‐10 and TGF‐β. However, when dysregulated, the IL‐17 response can promote infection.^[^ ^38^ ^]^ In the present study, we found that HNVs inhibited DSS‐induced inflammation by inducing Foxp3⁺ Tregs and suppressing IL‐17⁺CD4⁺ T cells.

**Figure 8:**
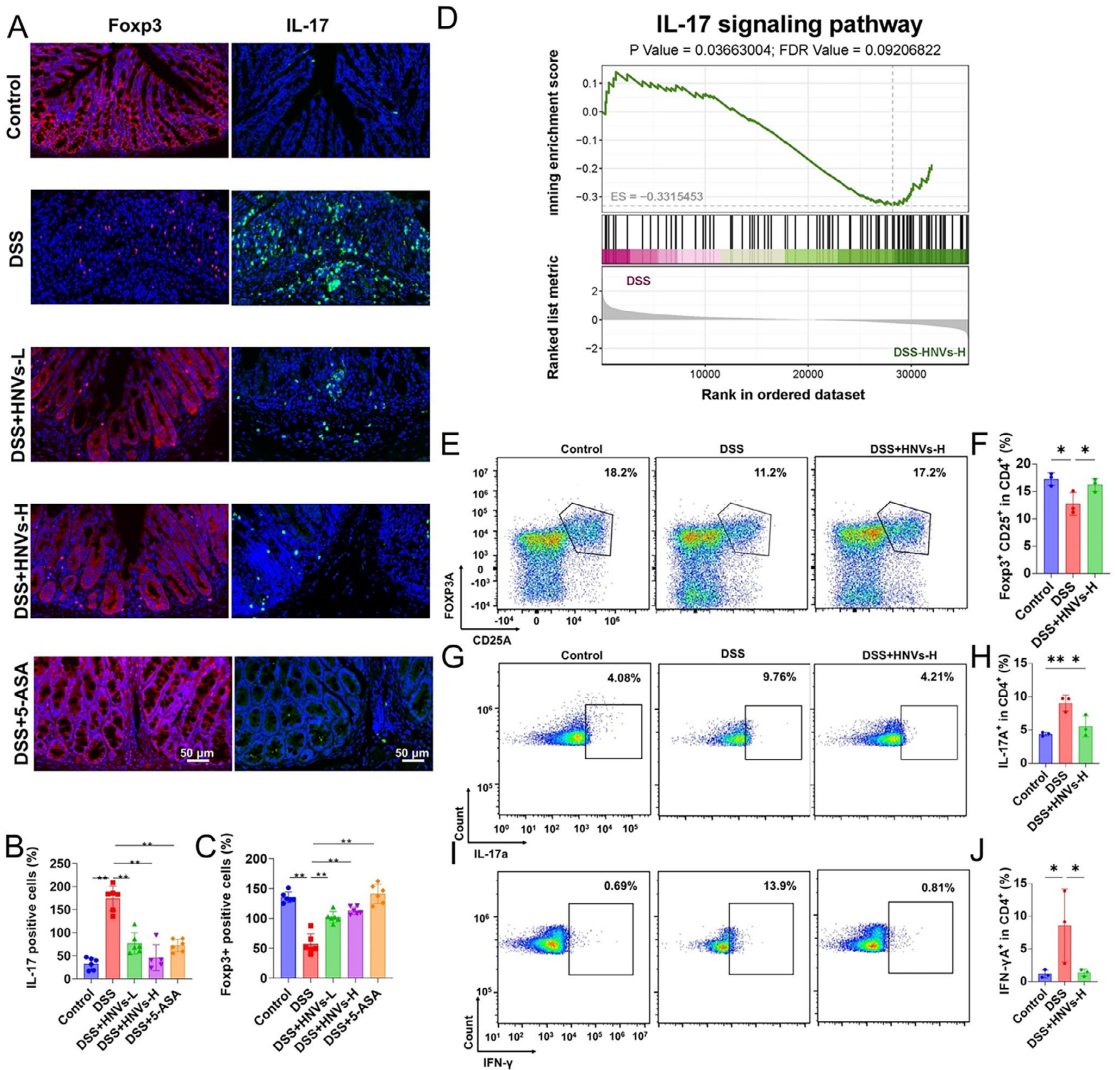
Effects of HNVs treatment on the balance of Th17/Treg cells in DSS‐induced experimental IBD in C57BL/6 mice. A) Colonic tissue immunofluorescence showing expression of Foxp3 and IL‐17. B–C) Quantification of Foxp3⁺ and IL‐17⁺ cells in colonic tissues using ImageJ2x software. D) GSEA of the IL‐17 signaling pathway. E–J) Flow cytometry analysis of Treg cells E,F), Th17 cells G,H), and Th1 cells I,J) in the spleen. Data are presented as mean ± SD (*n* = 3), and *P*‐values were determined using one‐way ANOVA. *P < 0.05, **P < 0.01, ns: not significant. HNVs‐H: high‐dose treatment group. Scale bar = 50 µm.

To confirm this, the expression of Foxp3 and IL‐17 proteins in colonic tissues was examined across the five groups (**Figure** **8**A). HNVs treatment contributed to immunosuppression in the colonic mucosa, as indicated by a marked increase in Foxp3⁺ cells and a decrease in the area of IL‐17–positive cells (Figure 8B,C). In addition, RNA‐seq analysis revealed significant downregulation of pro‐inflammatory pathways, including the IL‐17 signaling pathway, in colon tissues from the DSS + HNVs‐H group compared with the DSS group (Figure 8D). Consistently, we observed downregulation of pro‐inflammatory IL‐17 and upregulation of the anti‐inflammatory cytokine IL‐10 in HNVs‐treated mice.

Furthermore, it was found that HNVs treatment inhibited DSS‐induced inflammation by inducing Foxp3⁺ Tregs and suppressing IL‐17⁺CD4⁺ T cells and IFN‐γ⁺CD4⁺ T cells in the spleen. These findings suggest that HNVs may promote mucosal healing by rebalancing the CD25⁺Foxp3⁺ Treg and IL‐17⁺CD4⁺ T cell responses (Figure 8E–J).

### 2.9 HNVs do not Alleviate Colitis Symptoms in DSS‐Induced Mice with Depleted Gut Microbiota

**Figure 9:**
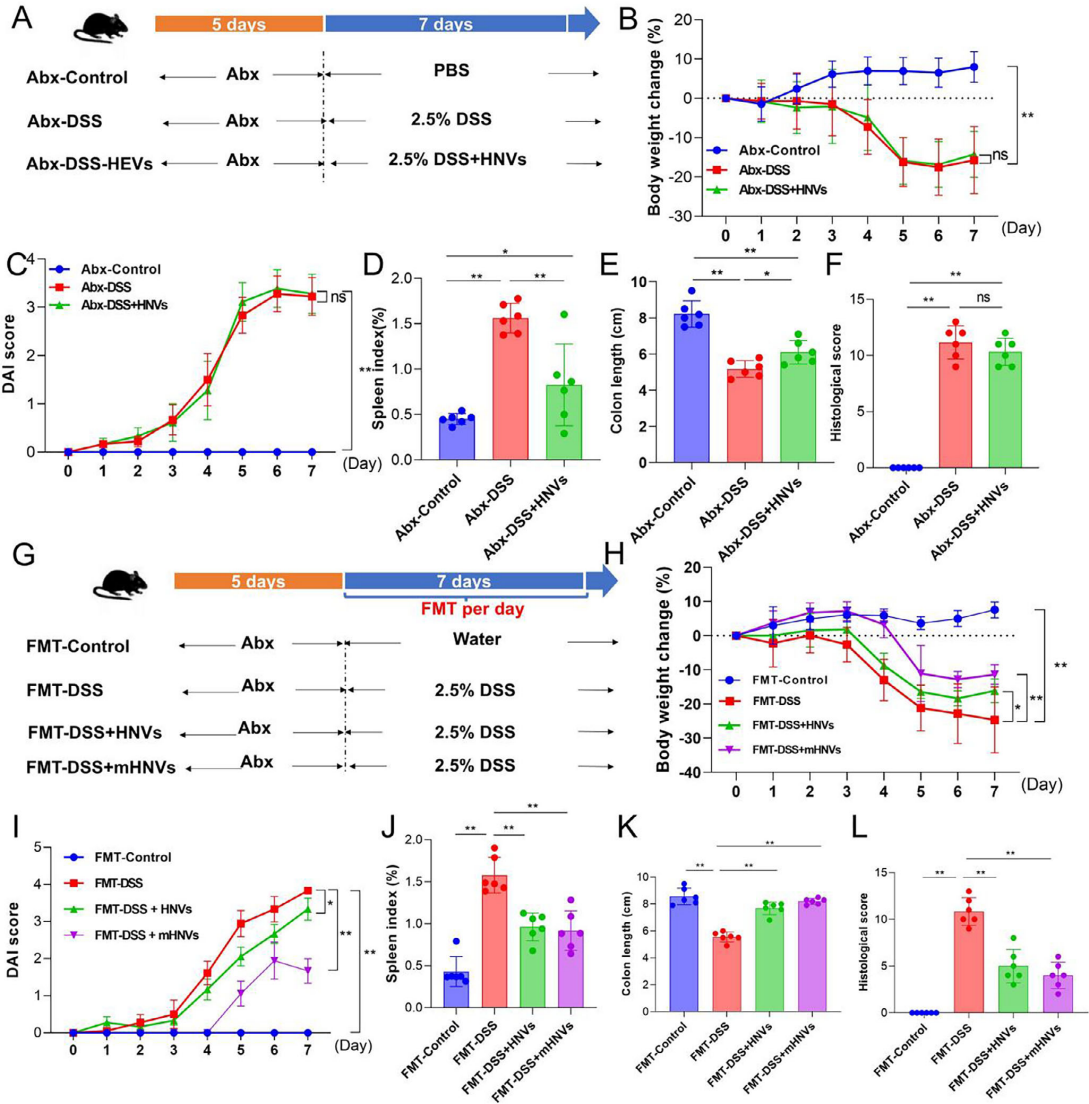
Effects of HNVs treatment on DSS‐induced experimental IBD symptoms in C57BL/6 mice with depleted gut microbiota. A) Schematic illustration depicts the establishment of the DSS‐induced IBD model. B) Body weight changes over 10 days. C) Disease activity index score over 10 days. D) Spleen index. E) Colon length. F) Histopathological changes were scored based on HE staining analysis. G) Schematic illustration of DSS‐induced IBD model mice. H) Body weight gain over 10 days. I) Disease activity index score over 10 days. J) Spleen index. K) Colon length. L) Histopathological changes were scored by HE staining. Data are presented as mean ± SD (*n* = 6), and *P*‐values were determined using one‐way ANOVA. *P < 0.05, **P < 0.01, ns: not significant.

To assess whether the therapeutic effects of HNVs are mediated by alterations in the gut microbiota and associated metabolites, we established a fecal microbiota transplantation (FMT) model in wild‐type (WT) C57BL/6 mice (**Figure** **9**A). The native gut microbiota was depleted using a broad‐spectrum antibiotic cocktail consisting of neomycin (Neo), vancomycin (Van), metronidazole (Metro), and ampicillin (Amp; Abx).^[^ ^39^ ^]^ In microbiota‐depleted DSS‐induced mice, HNVs treatment did not result in significant improvements in body weight, DAI scores, or colon length compared with the DSS group (Figure 9B–E).

Furthermore, HNVs treatment failed to restore gut barrier integrity, as evidenced by severe crypt destruction, histological disorganization, extensive immune cell infiltration, and significant epithelial damage in the inflamed colon (Figure S10A, B, Supporting Information and Figure 9F). These findings suggest that the presence of fecal microbiota is essential for HNVs to exert their therapeutic effects.

Next, FMTs were prepared from mice subjected to different treatments. Specifically, fecal bacterial suspensions from healthy mice, HNVs‐treated mice, and DSS‐induced mice were administered via oral gavage to re‐establish the gut microbiota in microbiota‐depleted (Abx‐treated) mice (Figure 9G). As expected, weight loss, DAI scores, colon shortening, spleen index, and histopathological lesions were significantly ameliorated in the FMT‐HNVs group compared with the DSS group (Figure S10C,D, Supporting Information, and Figure 9H–L).

Intriguingly, the heat‐inactivated fecal bacterial suspension (FMT‐mHNVs) exhibited an even greater protective effect than the FMT‐HNVs group in Abx‐treated DSS‐induced mice. These results suggest that the fecal microbiota from HNVs‐treated mice may contain specific beneficial microbes or metabolites that confer protection against DSS‐induced colitis.

To explore this further, a full‐length microbial diversity analysis was conducted using third‐generation biological sequencing to determine the composition of live and heat‐inactivated fecal bacterial suspensions. Alpha diversity indices (ACE, Simpson, and Shannon) showed no statistically significant differences between the FMT‐HNVs and FMT‐mHNVs groups (Figure S11A–C, Supporting Information). However, principal coordinate analysis revealed that HNVs treatment resulted in significant changes in gut microbial community structure (Figure S11D–E, Supporting Information).

Next, the relative abundance of gut microbiota in the control and FMT‐HNVs groups was examined. At the phylum level, HNVs treatment increased the abundance of Bacillota and Campylobacter while decreasing the abundance of Bacteroidota and Verrucomicrobiota (Figure S11F, Supporting Information). At the species level, HNVs treatment significantly increased the abundance of the beneficial bacterium *Limoxilatobalacillus reuteri* (Figure S11G, Supporting Information). The LEfSe species hierarchy and bar chart analyses further confirmed the dominance of beneficial bacteria such as *Limosilatobacterus reuteri* and *Ligilatobacterus murinus* in the HNVs group (Figure S11H–I, Supporting Information).

In addition, nontargeted mass spectrometry was used to detect metabolites in the thermally inactivated and active fecal suspensions. PCA and PLS‐DA analyses showed clear separation between the FMT‐HNVs and FMT‐mHNVs groups (Figure S12A,B, Supporting Information). Venn diagram analysis revealed 2496 shared metabolites between the two groups, with 94 unique to FMT‐HNVs and 57 unique to FMT‐mHNVs (Figure S12C, Supporting Information). Furthermore, bar chart analysis showed that, compared to FMT‐mHNVs, the FMT‐HNVs group exhibited 214 upregulated and 186 downregulated differential metabolites (Figure S12D, Supporting Information). Heatmap analysis illustrated distinct patterns of upregulation and downregulation in metabolites in the FMT‐mHNVs group compared to the FMT‐HNVs group (Figure S12E, Supporting Information).

Importantly, FMT‐mHNVs treatment significantly increased the levels of beneficial metabolites such as 4‐coumaric acid, taurochenodeoxycholic acid, and lysophosphatidylethanolamines (LPEs) (Figure S12F, Supporting Information). These findings indicate that FMT using heat‐inactivated bacterial suspension (FMT‐mHNVs) exerts superior protective effects compared with live bacterial suspension (FMT‐HNVs), likely due to the presence of specific beneficial microbes or their metabolites in the inactivated form.

## 3 Discussion

Recent studies have demonstrated that HNVs are capable of entering mammalian cells, where they exert various beneficial biological effects, including the maintenance of intestinal immune homeostasis, attenuation of inflammatory responses, and facilitation of tissue regeneration.^[^ ^28^ ^]^ In the present study, we aimed to explore the anti‐inflammatory potential of HNVs using a DSS‐induced colitis model. Our findings indicate that HNVs significantly suppress the inflammatory response in mice with DSS‐induced colitis. These anti‐inflammatory effects contribute to the improvement of colitis symptoms through several mechanisms, including downregulation of pro‐inflammatory cytokines, reduction of immune cell infiltration, protection of the mucosal barrier, modulation of gut microbiota composition and BA metabolism, enhancement of SCFA production, and rebalancing of CD25⁺Foxp3⁺ Treg and IL‐17⁺CD4⁺ T cell responses.

Intestinal inflammation and mucosal tissue damage are hallmarks of IBD, although the exact etiology remains unclear.^[^ ^40^ ^]^ Abnormal immune responses are considered one of the key pathogenic mechanisms of IBD.^[^ ^41^ ^]^ T lymphocytes are central to antigen presentation in immune responses and act as important immunoregulatory cells involved in various pathways of mucosal inflammation.^[^ ^42^ ^]^ Localized inflammatory reactions can trigger systemic immune responses through multiple pathways, resulting in alterations in several immune parameters, including increased expression of CD3⁺ and CD8⁺ T lymphocytes and related inflammatory markers in the bloodstream.^[^ ^43^ ^]^ One study reported that damage to the intestinal mucosal barrier allows bacterial toxins such as lipopolysaccharide (LPS) to penetrate the mucosa and enter the bloodstream, leading to T cell activation and an increased T cell ratio.^[^ ^44^ ^]^ Moreover, the proportion of CD3⁺ and CD8⁺ T cells in the peripheral blood of IBD patients has been shown to be significantly higher than in healthy controls.^[^ ^45^ ^]^ This elevation may be due to the systemic release of bacteria and their toxins through the compromised mucosal barrier, leading to the generation of inflammatory mediators and cytokines. Additionally, the proportions of CD4⁺ and CD8⁺ T lymphocyte subsets are associated with pro‐inflammatory cytokine levels, and the activation of T lymphocytes and elevation of serum inflammatory markers are closely correlated with IBD severity.^[^ ^46^ ^]^

In our study, we found that treatment with HNVs significantly reduced DSS‐induced activation of CD3⁺ and CD8⁺ T cells and lowered pro‐inflammatory cytokine levels in the blood. MPO, which is predominantly expressed by neutrophils, promotes the formation of reactive molecules such as hypochlorous acid at sites of inflammation and plays a critical role in the pathogenesis of IBD.^[^ ^47^ ^]^ In this study, MPO levels were significantly elevated in the colonic tissues of DSS‐treated mice but were notably reduced following HNVs treatment, underscoring the anti‐inflammatory potential of HNVs in the context of IBD.

Macrophages also play a crucial role in both innate and adaptive immunity by clearing apoptotic cells and pathogens, thereby maintaining immune homeostasis. However, their excessive activation and infiltration can lead to pathological inflammation, particularly in immune‐mediated diseases like IBD.^[^ ^25^ ^]^ In IBD, hyperactivation of M1 macrophages and their increased secretion of pro‐inflammatory cytokines contribute to disease progression. In contrast, M2 macrophages are essential for tissue repair and resolution of inflammation, leading to symptom alleviation.^[^ ^48^ ^]^ Polarization of macrophages toward the M1 phenotype has been strongly implicated in IBD pathogenesis, while therapies targeting TNF‐α have been successful in reducing disease severity by shifting macrophage polarization toward the M2 phenotype.^[^ ^49^ ^]^ Thus, modulation of macrophage polarization is increasingly recognized as a promising therapeutic approach in IBD.

In this study, we observed that HNVs treatment in DSS‐induced colitis mice significantly enhanced M2 macrophage polarization and concurrently reduced levels of pro‐inflammatory cytokines. These findings support the notion that HNVs have robust mucosal repair properties and therapeutic potential for clinical application. Furthermore, treatment with HNVs or the standard anti‐inflammatory drug 5‐ASA led to a marked reduction in pro‐inflammatory cytokines, including MCP‐1, TNF‐α, IL‐6, and IFN‐γ, alongside significant upregulation of the anti‐inflammatory cytokine IL‐10 when compared with the DSS group.

Collectively, these results demonstrate that HNVs mitigate DSS‐induced colitis by restoring mucosal barrier integrity and suppressing inflammatory responses.

The translocation and colonization of bacteria within the mucosal layer have been implicated in the pathogenesis of both metabolic disorders and IBDs, including UC and Crohn's disease.^[^ ^50^, ^51^ ^]^ In UC, a predominant form of IBD, defects in the mucus barrier often precede disease onset and serve as a key contributing factor.^[^ ^52^ ^]^ Similarly, mice lacking occludin and ZO‐1, which are critical structural components of the colonic barrier, or deficient in the predominant O‐linked oligosaccharides that modify Muc2, develop severe colitis, thereby confirming a causal link between mucus dysfunction and intestinal inflammation.^[^ ^53^ ^]^ In our present study, the protein levels of occludin, ZO‐1, and Muc2 were significantly reduced in the colonic tissues of DSS‐treated mice, whereas treatment with HNVs effectively restored the expression of occludin, ZO‐1, and Muc2. Moreover, HNVs exerted protective effects on the colonic mucosal barrier, as evidenced by the increased presence of Ki67‐positive proliferating cells.

To further clarify the anti‐inflammatory effects of HNVs in DSS‐induced colitis, we examined their impact on gut microbial composition, SCFA production, and BA metabolism. Extensive research has demonstrated that inflammatory diseases are frequently accompanied by substantial changes in gut microbiota, which are strongly associated with dysbiosis and exacerbated inflammatory responses.^[^ ^54^ ^]^ This underscores the potential of inducing favorable shifts in gut microbial communities as a means to restore immune homeostasis.^[^ ^55^ ^]^ In our study, DSS‐treated mice exhibited a reduction in Firmicutes levels, along with an increase in Proteobacteria and Campylobacterota, which were successfully reversed by HNVs treatment. At the genus level, administration of HNVs notably increased the abundance of beneficial bacteria such as *Eubacterium* and *Turicibacter*, while significantly reducing pathogenic bacteria including *Escherichia‐Shigella* and *Bacteroides*. These findings highlight the critical role of HNVs in modulating gut microbiota composition, thereby contributing to the alleviation of colitis symptoms.

Proteobacteria, a dominant phylum of gram‐negative bacteria, encompasses important taxa such as Enterobacteriaceae, as well as a wide range of pathogens including Salmonella and Helicobacter, commonly found in both humans and animals. A hallmark feature of many Proteobacteria species is their LPS‐rich outer membrane, which acts as a potent activator of innate immunity and can trigger both localized and systemic immune responses.^[^ ^56^ ^]^ The aberrant expansion of Proteobacteria is widely recognized as an indicator of gut dysbiosis and is closely linked to an elevated risk of disease.^[^ ^57^ ^]^ Although Enterobacteriaceae account for less than 1% of the healthy human gut microbiota, the family includes both commensal and opportunistic pathogens. Under conditions of gut inflammation, these opportunistic species can proliferate, leading to significant microbial dysregulation. Escherichia‐Shigella, a pathogenic bacterium frequently associated with inflammatory diseases such as colitis, has been shown to activate the NLRP3 inflammasome.^[^ ^58^ ^]^ Thus, the inhibitory effect of HNVs on Escherichia‐Shigella, along with their ability to enhance the growth of beneficial bacteria such as Eubacterium and Turicibacter, likely plays a pivotal role in mitigating colonic inflammation and provides insight into the underlying therapeutic mechanisms of HNVs in IBDs.

In the field of nutrition, certain bacterial species, including members of the phylum Firmicutes and the genus *Bifidobacterium*, are involved in the biosynthesis of various compounds, including vitamins K and B.^[^ ^59^ ^]^ Furthermore, some of these bacteria, such as *Eubacterium* and *Turicibacter*, are capable of producing SCFAs through the fermentation of dietary fibers.^[^ ^60^ ^]^ SCFAs are low‐molecular‐weight fatty acids containing two to five carbon atoms, synthesized by anaerobic microorganisms. Once absorbed, SCFAs exert systemic immunoregulatory and anti‐inflammatory effects.^[^ ^61^ ^]^ The colon contains high concentrations of SCFAs, primarily acetate, propionate, and butyrate. These acids facilitate the growth of beneficial bacteria and stimulate Treg cells, thereby suppressing inflammatory mediators.^[^ ^62^ ^]^ Additionally, they promote increased colonic oxygen consumption by epithelial cells, enhance immune regulation, and strengthen gut barrier function.^[^ ^63^ ^]^ In this study, the cecal concentrations of acetate and butyrate were significantly higher in the DSS + HNVs group compared to the DSS group. Furthermore, HNVs markedly decreased the BA DBH while increasing NorDCA levels in DSS‐induced mice. Collectively, these results indicate that HNVs induce substantial remodeling of gut microbiota‐derived metabolite profiles, particularly by enhancing SCFA production.

The upregulation of chemokine signaling observed in DSS‐treated mice aligns with the well‐established role of chemokines in recruiting immune cells to inflamed intestinal sites. Chemokines such as CXCL1, CXCL2, and CCL20 are elevated in IBD patients and promote the infiltration of neutrophils and Th17 cells, thereby perpetuating mucosal damage.^[^ ^64^ ^]^ Our RNA‐seq data showing increased chemokine signaling suggest that DSS‐induced colitis recapitulates this chemokine‐driven leukocyte trafficking, further exacerbating disease severity. In addition, the observed activation of cAMP signaling may reflect a compensatory anti‐inflammatory response. cAMP inhibits NLRP3 inflammasome activation and suppresses NF‐κB signaling in macrophages, thereby reducing the production of IL‐1β and TNF‐α.^[^ ^65^ ^]^ In IECs, cAMP enhances barrier function through PKA‐mediated tightening of tight junctions.^[^ ^66^ ^]^ Paradoxically, DSS‐induced mucosal damage may transiently elevate cAMP levels in an attempt to counteract inflammation, although this response is insufficient to fully resolve the pathology. Notably, the therapeutic effects of HNVs included the downregulation of cAMP signaling.

With growing evidence linking gut microbiota to IBD, the intricate interplay between microbiota, metabolites, and the immune system has garnered significant attention.^[^ ^67^ ^]^ The digestive tract, being continuously exposed to trillions of microorganisms, inherently triggers immune responses to a broad spectrum of exogenous stimuli.^[^ ^68^ ^]^ The intestinal mucosa maintains homeostasis by regulating host immune responses to commensal microbes, primarily through the action of key metabolites such as butyrate.^[^ ^69^ ^]^ Butyrate, a key SCFA, exerts potent anti‐inflammatory effects by activating G protein‐coupled receptors GPR41 and GPR43.^[^ ^70^ ^]^ Additionally, butyrate promotes the differentiation of Treg cells through a Foxp3‐dependent pathway. These Foxp3⁺ Treg cells play a crucial role in suppressing excessive inflammation by secreting IL‐10.^[^ ^71^ ^]^ This mechanism highlights the link between reduced butyrate levels and increased intestinal inflammation, as butyrate plays a critical role in suppressing the release of pro‐inflammatory cytokines, such as TNF‐α, from intestinal lamina propria mononuclear cells. Moreover, the overactivation of Th1/Th17 responses and the damage to Foxp3⁺ Treg cells have been described as key pathogenic mechanisms of IBD.^[^ ^72^ ^]^ Treg‐mediated immune suppression through the secretion of inhibitory cytokines such as TGF‐β and IL‐10 is of great significance in controlling IBD. These cells also protect intestinal physiology by promoting epithelial barrier function and facilitating tissue repair.^[^ ^73^ ^]^ Our findings that HNVs relieve host mucosal inflammation by rebalancing Treg/Th17 responses are consistent with studies demonstrating that SCFAs regulate GPR43‐dependent immunosuppression by colonic Tregs under DSS or T cell‐transfer conditions.^[^ ^74^ ^]^

FMT is an emerging therapeutic intervention that involves the transfer of processed stool from a healthy donor to a recipient with the goal of restoring a balanced gut microbiome. This approach is particularly relevant in IBD, where dysbiosis, characterized by reduced microbial diversity, depletion of beneficial bacteria (e.g., *Faecalibacterium prausnitzii*), and overgrowth of pathobionts (e.g., *Escherichia coli*), plays a central role in disease pathogenesis.^[^ ^75^, ^76^ ^]^ Unlike conventional immunosuppressive therapies, FMT targets the underlying microbial imbalance, offering a novel mechanism to modulate intestinal inflammation. Our results demonstrate that the inactivated fecal microbiota suspension (sterilized to eliminate live bacteria while retaining microbial components and metabolites) exhibits significantly greater therapeutic efficacy than its active counterpart in ameliorating IBD symptoms. This unexpected finding suggests that the beneficial effects of FMT may not depend solely on viable microorganisms but rather on microbial structural components (e.g., bacterial cell wall fragments, extracellular vesicles), stable metabolites (e.g., SCFA–related metabolites such as 4‐coumaric acid and tryptophan derivatives, immunomodulatory and anti‐inflammatory metabolites such as taurodeoxycholic acid, and intestinal barrier repair metabolites from the LPE class), as well as viral and fungal elements that survive inactivation. This paradigm‐shifting discovery challenges the fundamental premise that live microbiota transplantation is necessary for therapeutic benefit. The superior performance of inactivated FMT suspensions opens new avenues for developing safer, more standardized microbiome‐based therapies for IBD.

Although the multifunctional roles of PNVs are established, key mechanistic questions persist. Critical knowledge gaps include: (i) the specific pathways governing PNVs interactions with recipient cells and subsequent downstream alterations; and (ii) the systematic characterization of endocytic mechanisms facilitating PNVs internalization. Our future investigations will prioritize these unresolved areas by identifying bioactive components with therapeutic effects in HNVs, elucidating their target cell specificity, internalization pathways, and downstream functional impacts.

## 4 Conclusions

This study demonstrated the significant potential of HNVs as a promising oral therapeutic option for the treatment of IBD. HNVs act as excellent protective agents against DSS‐induced colitis in mice, operating through several critical mechanisms: a) modulation of the intestinal microbiota, b) alteration of gut microbial metabolites, c) improvement of the unbalanced Treg/Th17 response, and d) protection of the intestinal barrier, accompanied by a reduction in inflammation within both the colon and systemic circulation through microbe–host interactions. With their natural origin, remarkable therapeutic efficacy, and excellent biocompatibility, HNVs emerge as a highly promising candidate for clinical application in the management of IBD.

## 5 Experimental Section

### Honeysuckle

Honeysuckle was purchased from Tongrentang, a traditional Chinese medicine supplier. HD was prepared by boiling 10 g of dried honeysuckle in 500 mL of water for 30 min, followed by concentration to ≈ 50 mL of decoction. To obtain HJ, 100 g of dried honeysuckle were soaked in 1000 mL of sterile PBS for 2 h, yielding 500 mL of HJ, with 1 mL corresponding to 0.2 g of dried honeysuckle. Each mouse (weighing ≈ 20 g) was orally administered 200 µL of HD or HJ, equating to a daily dose of 2 g kg^−1^ of honeysuckle.

### Isolation and Characterization of HNVs

A total of 100 g of dried honeysuckle were soaked in 1000 mL of sterile PBS for 2 h. The flowers were then processed into juice using a high‐speed blender and stored at 4 °C overnight to optimize extraction. The resulting juice was filtered to remove large debris and sequentially centrifuged at 1000 × *g* for 10 min, 3000 × *g* for 30 min, and 10000 × *g* for 60 min to remove smaller particulates. The supernatant was subjected to ultracentrifugation at 150000 × *g* for 2 h. The resulting pellet was resuspended in sterile phosphate‐buffered saline, and HNVs were isolated using an ÄKTA tangential flow filtration system to yield 50 mL of HNVs. For treatment, the high‐dose (HNVs‐H) or low‐dose (HNVs‐L) groups received a daily gavage dose of 200 µL per 20 g mouse. The corresponding therapeutic doses of honeysuckle were 20 and 10 g kg^−1^ for the HNVs‐H and HNVs‐L groups, respectively. The protein concentration of the HNVs was determined using a bicinchoninic acid protein assay kit (Thermo Fisher Scientific, USA), with bovine serum albumin as the standard. For TEM, HNVs were fixed in 2% paraformaldehyde and stained with a solution containing 1.9% methylcellulose and 0.3% uranyl acetate for negative contrast. Imaging was performed using a Hitachi HT‐7700 transmission electron microscope. DLS analysis was conducted using a Zetasizer Nano ZSE (Malvern Instruments, UK) to assess particle size distribution.

### In Vivo Imaging of Mice

Mice were orally administered Cy5.5‐labeled HNVs. Imaging was conducted at 3, 6, 12, and 24 h post‐administration. At each time point, mice were placed into the IVIS imaging system for fluorescence detection. After imaging, the mice were euthanized, and major organs, including the heart, kidneys, lungs, spleen, liver, stomach, small intestine, and colon, were harvested. Imaging was performed using the IVIS Spectrum system (PerkinElmer, Waltham, MA, USA) with a DIR filter channel and a 5 s exposure time. Fluorescence intensity was quantified for each organ.

### Detection of the Stability of HNVs

To evaluate the stability of HNVs under in vitro conditions simulating the gastrointestinal environment, 100 µL of HNVs were incubated with 300 µL of simulated gastric and intestinal fluids at 37 °C for 0, 2, 4, 6, and 8 h. After each time point, the size distribution of the HNVs was assessed using DLS (Zetasizer Nano ZSE; Malvern Instruments, UK) to detect potential changes in particle size.

### Coomassie Brilliant Blue Staining

For visualization of proteins, polyacrylamide gels were stained with Coomassie Brilliant Blue (P0017B; Beyotime Biotechnology) under gentle shaking for 2 h at room temperature, followed by destaining (P0017C; Beyotime) for 30 min. The stained gels were imaged using a Bio‐Rad ChemiDOC MP Imaging System (Hercules, CA, USA).

### Nucleic Acid and Protein Analysis

Total RNA was extracted from FAELNs using TRIzol reagent (Thermo Fisher Scientific, Waltham, MA, USA). RNA concentration was measured using a NanoDrop spectrophotometer, and RNA integrity was assessed with a Bio‐Fragment Analyzer to determine the RNA quality number.

### miRNA‐Seq Analysis

miRNA‐Seq was performed by LC‐Bio Technologies (China) using Illumina protocols. Small RNA libraries were prepared using TruSeq kits and sequenced on HiSeq 2000/2500 platforms (Illumina, USA), generating 50 bp single‐end reads.

### Liposome Sequencing Analysis

HNVs were processed in microcentrifuge tubes containing glass beads and a chloroform:methanol mixture (2:1, v/v). Samples were subjected to vortex mixing, freeze–thaw cycles, and agitation. After incubation on ice, water was added to enable phase separation via centrifugation. The extraction was repeated with fresh solvent, and the combined organic phases were concentrated, redissolved in isopropanol, filtered through 0.22 µm membranes, and analyzed by liquid chromatography–mass spectrometry (LC‐MS).

### Metabonomic Sequencing Analysis

Exosome preparations were extracted in 2 mL tubes containing glass beads and 1000 µL of a 2:2:1 acetonitrile:methanol:water mixture. Samples were subjected to vortexing, freeze–thaw cycles, and two rounds of grinding for 2 min each at 60 Hz. Following centrifugation (12000 rpm, 4 °C, 10 min), the supernatant was collected, dried, and redissolved in 300 µL of 1:9 acetonitrile:0.1% formic acid containing 4 ppm 2‐chloro‐L‐phenylalanine. The solution was then filtered and subjected to LC‐MS analysis.

### Mouse Model of DSS‐Induced Colitis

Male C57BL/6 mice, aged 6–8 weeks and weighing ≈ 20 g, were obtained from Gem Pharmatech (Shanghai, China). Mice were housed under specific pathogen‐free conditions, with a 12 h light/dark cycle, controlled temperature (24 °C ± 1 °C), and relative humidity (50–60%). Food and water were provided ad libitum. All experimental procedures were approved by the Ethics Committee of Zhejiang Provincial People's Hospital (Approval No. 202312250713117239317).

Mice were housed six per cage and acclimatized for one week before the experiment. Animals were randomly assigned to five groups: a healthy control group (blank control), a DSS‐induced colitis group (negative control), a 5‐ASA treatment group (positive control, as 5‐ASA is a standard first‐line therapy for IBD), and two HNVs treatment groups, one receiving low‐dose HNVs (10 g kg^−1^, DSS + HNVs‐L) and the other high‐dose HNVs (20 g kg^−1^, DSS + HNVs‐H). Colitis was induced by administering 2.5% (*w*/*v*) DSS (molecular weight 36000–50000; MP Biomedicals, USA) in the drinking water for 7 days, followed by a 3‐day treatment period. During these 10 days, 200 µL of PBS, 5‐ASA (0.1 g kg^−1^), or HNVs (10 or 20 g kg^−1^) were administered daily via oral gavage. Mice were monitored daily for changes in body weight, stool consistency, and presence of fecal blood to calculate the DAI. On day 11, all mice were euthanized, and samples from the intestine, spleen, liver, and cecum were collected for further analysis. Comprehensive hematological analyses were also performed on collected blood samples.

### Antibiotic Administration, FMT, and Bacterial Colonization

Mice received daily oral gavage of a freshly prepared antibiotic cocktail (Abx), consisting of Metro (1 g L^−1^), Amp (1 g L^−1^), Van (0.5 g L^−1^), and Neo (1 g L^−1^), for 5 consecutive days. On the fifth day after the final treatment, fecal samples were collected from microbiota‐depleted mice, homogenized, and cultured on Brain Heart Infusion agar plates supplemented with 10% sheep blood for microbial growth analysis. The plates were initially incubated under anaerobic conditions at 37 °C for 2 days, followed by an additional 1‐day incubation under aerobic conditions at the same temperature to confirm the effective depletion of gut microbiota. For the FMT experiment, fecal pellets (200 mg) collected from WT mice that had received different treatments (oral administration of PBS or HNVs) were homogenized in 1.5 mL of PBS using sterile silicone beads at a frequency of 45 Hz for 1 min, and then filtered through a 70 µm sieve. WT mice pre‐treated with antibiotics were subsequently administered 200 µL of the filtered fecal homogenate via oral gavage to perform the FMT procedure.

### Evaluation of Colitis Severity

The DAI, a widely accepted scoring method, was used to assess colitis severity based on three main parameters: body weight loss, fecal blood presence, and stool consistency. Each parameter was scored on a scale from 0 to 4 to reflect the severity of symptoms.^[^ ^77^ ^]^ In addition, colon length, measured after euthanasia, was used as a supplementary indicator of colitis severity.

### Histological Analysis, Immunohistochemical Staining, and In Vivo Imaging

Colon tissues were fixed in 4% paraformaldehyde, dehydrated, embedded in paraffin, and sectioned into 4 µm slices. The tissue sections were stained with HE following standard protocols,^[^ ^78^ ^]^ Goblet cells were visualized by periodic acid‐Schiff staining for 10–15 min, followed by dehydration with 100% ethanol and xylene. For immunofluorescence analysis, tissue sections were blocked with 10% normal goat serum for 30 min and incubated with primary antibodies at 4 °C for 12 h. The following primary antibodies were used: anti‐CD3 (ABclonal), CD8 (CST), anti‐MPO (ABclonal), anti‐Ki67 (CST), anti‐CD68 (CST), anti‐CD163 (ABclonal), anti‐occludin (ABclonal), anti‐ZO‐1 (ABclonal), and anti‐mucin 2 (Muc2) (GTX). Slides were subsequently incubated with fluorescently labeled secondary antibodies (Jackson ImmunoResearch Company), and nuclei were stained with DAPI (Roche, Switzerland). All imaging and quantification analyses were conducted using ImageJ software.

### Cell Culture

RAW264.7 cells were cultured in tissue culture plates using Dulbecco's modified Eagle's medium, supplemented as described previously. Cells were maintained at 37 °C in a humidified atmosphere containing 5% CO_2_.

### Cellular Uptake

To assess HNVs uptake in RAW264.7 cells, cells were seeded in confocal dishes at a density of 1 × 10⁵ cells per dish and incubated for 24 h. After incubation, the culture medium was replaced with fresh medium containing HNVs, and cells were incubated for 0, 3, 6, 12, and 24 h. Hoechst staining solution was added for nuclear staining and incubated for 15 min, followed by three washes with PBS. CLSM was used for imaging.

### Analysis by Enzyme‐Linked Immunosorbent Assay Analysis (ELISA)

Serum levels of MCP‐1, TNF‐α, IL‐6, IFN‐γ, and IL‐10 were quantified using commercial ELISA kits, following the manufacturer's instructions.

### Fluorescein Isothiocyanate‐Conjugated (FITC)‐Dextran Permeability Test

Mice were fasted overnight prior to oral gavage with FITC‐dextran (3–5 kDa; Sigma‐Aldrich) at a dose of 60 mg per 100 g of body weight. Four hours after administration, mice were euthanized, and blood samples were collected. Serum concentrations of FITC‐dextran were measured using a fluorescence microplate reader with excitation at 490 nm and emission at 525 nm. FITC‐dextran concentration and intestinal permeability were calculated using a standard curve.

### 16S rRNA‐seq

Total fecal DNA was extracted using the CTAB method to ensure comprehensive isolation of microbial genetic material. The V3–V4 regions of 16S rDNA were amplified via PCR. The PCR products were purified, quantified, and evaluated to ensure a final library concentration exceeding 2 × 10^− 9^ m. After sequencing, raw reads were processed and denoised using the DADA2 algorithm to obtain amplicon sequence variants (ASVs). ASV sequences and abundance tables were used for alpha and beta diversity analysis. Statistical analysis of species abundance across samples was performed using ASV tables, and intergroup differences were assessed based on relative species abundance. All sequencing and analysis procedures were conducted by LC‐Bio Technology Co., Ltd. (Hangzhou, China).

### RNA‐Seq

Total RNA was first extracted from the samples, and the quantity, purity, and integrity of the RNA were assessed to ensure a RIN value greater than 7.0. The extracted RNA was then fragmented and reverse transcribed into complementary DNA (cDNA), followed by second‐strand cDNA synthesis. An adenine nucleotide was incorporated into the cDNA fragments, after which size selection was performed and uracil residues were removed. Subsequently, the ligated DNA fragments underwent PCR amplification. Following sequencing, the raw data were processed using Fastp software for quality control and preprocessing. Transcriptomes from all samples were integrated using gffcompare to construct a comprehensive transcriptome profile. Expression levels were quantified by StringTie based on FPKM values. Differentially expressed mRNAs were identified using the edgeR package, with thresholds set at a fold change greater than 2 or less than 0.5, and a *P*‐value below 0.05.

### Detection and Analysis of Metabolites in Cecum Contents

For the analysis of SCFAs and BAs, 50 mg of fecal material was first homogenized using a KZ‐II grinding machine. After homogenization, the samples were centrifuged at 12000 × *g* for 15 min at 4 °C. The resulting supernatants were filtered through 0.22 µm membranes and subsequently analyzed using gas chromatography–mass spectrometry or LC‐MS.

### 16S Third‐Generation Sequencing

Microbial community profiling was performed using third‐generation 16S rRNA‐seq (Majorbio , Shanghai, China) on the PacBio Sequel II platform. This technique employed single‐molecule real‐time technology to generate full‐length 16S amplicons (∼1.5 kb), enabling high‐resolution taxonomic classification. Circular consensus sequencing was applied to achieve > 99% accuracy through multiple passes of each DNA molecule.

### Nontargeted Mass Spectrometry

Global metabolite analysis was conducted using the Majorbio's nontargeted LC‐MS/MS workflow, which employed high‐resolution accurate mass spectrometry with electrospray ionization. Chromatographic separation was performed on a C18 column (2.1 × 100 mm, 1.7 µm) using a 0.1% formic acid/acetonitrile gradient over 18 min.

### Flow Cytometry

To perform cell surface staining, single‐cell suspensions were incubated on ice with the following antibodies for 30 min: FITC‐conjugated anti‐CD3 (BD Pharmingen), APC‐conjugated anti‐CD4 (BD Pharmingen), and BV421‐conjugated anti‐CD25 (BD Pharmingen). For Treg cell analysis, after staining cells with CD3, CD4, and CD25 antibodies, the lymphocyte suspension was fixed and permeabilized using the transcription factor buffer set (BD Pharmingen), and stained with anti‐Foxp3 PE (ThermoFisher), according to the manufacturer's instructions. To analyze Th1 and Th17 cells, isolated tissue lymphocytes were stimulated with a cell stimulation cocktail and a protein transport inhibitor (eBioscience). Cells were then fixed in a fixation buffer (eBioscience), permeabilized with intracellular staining permeabilization wash buffer (eBioscience), and stained with anti‐IL‐17A (BD Pharmingen) conjugated to PerCP‐Cyanine5.5 and anti‐IFN‐γ (BD Pharmingen) conjugated to PE‐CY7.

### Statistical Analysis

Statistical analyses were performed using GraphPad Prism software, version 8.0. In all figures, error bars represent standard deviation (SD), and P‐values were determined using one‐way ANOVA or t‐tests. A P‐value of less than 0.05 was considered statistically significant.

## Conflict of Interest

The authors have declared that no competing interests exist.

## Author Contributions

Y. Wang and Y. Zhou are the co‐first authors and contributed equally. Yuanyuan Wang, Xiaozhou Mou, and Huiyu Liu designed the study; Yuanyuan Wang, Yuanhao Zhou, and Hai Zou performed and analyzed the biological experiments; Yuanyuan Wang and Zhenye Lv conducted the literature review and drew the chemical structures; Yishu Li, Yilin Huang, Kexin Yu, Haotian Liu, and Ping Li assisted with the animal experiments; Yuanyuan Wang and Huiyu Liu wrote the manuscript; Qingyuan Wu, Bingxian Yang, and Hai Zou supervised and guided the chemical analyses.

## Supporting information



Supporting Information

Supporting Information

## Data Availability

The data that support the findings of this study are available from the corresponding author upon reasonable request.
