# Supplementary material for: Honeysuckle‐Derived Nanovesicles Regulate Gut Microbiota for the Treatment of Inflammatory Bowel Disease
Source: Adv Sci (Weinh). 2025 Sep 19;12(45):e05208. doi: 10.1002/advs.202505208 (PMC12677632; doi:10.1002/advs.202505208)
Supplement: Supplementary file 1 — Supporting Information [file ADVS-12-e05208-s002.docx]

Supporting Information

**Honeysuckle-Derived Nanovesicles Regulate Gut Microbiota for the Treatment of Inflammatory Bowel Disease**

*Yuanyuan Wang^†^,* *Yuanhao Zhou^†^,* *Qingyuan Wu,* *Yishu Li,* *Yilin Huang, Kexin Yu, Ping Li,* *Zhenye Lv, Haotian Liu**, Hai Zou*,* *Huiyu Liu*,* *Xiaozhou Mou**

**
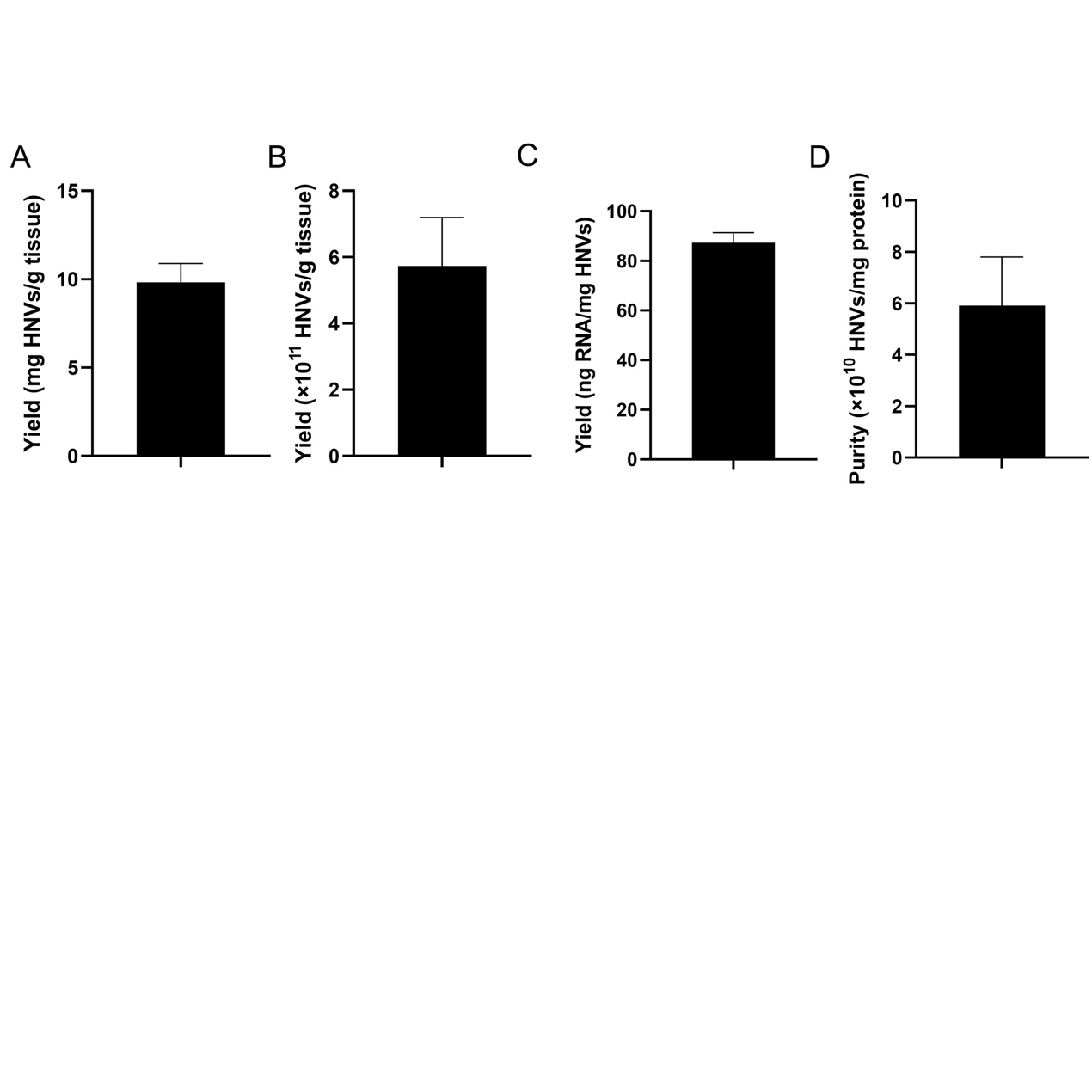
**

**Figure S1.** Purification and characterization of honeysuckle-derived nanovesicles (HNVs). Quantification of HNV yield by weight (A), and number (B). (C) Quantification of RNA yield from HNVs. (D) Quantification of HNV purification from HNVs. Data are presented as mean ± SD (n = 3).


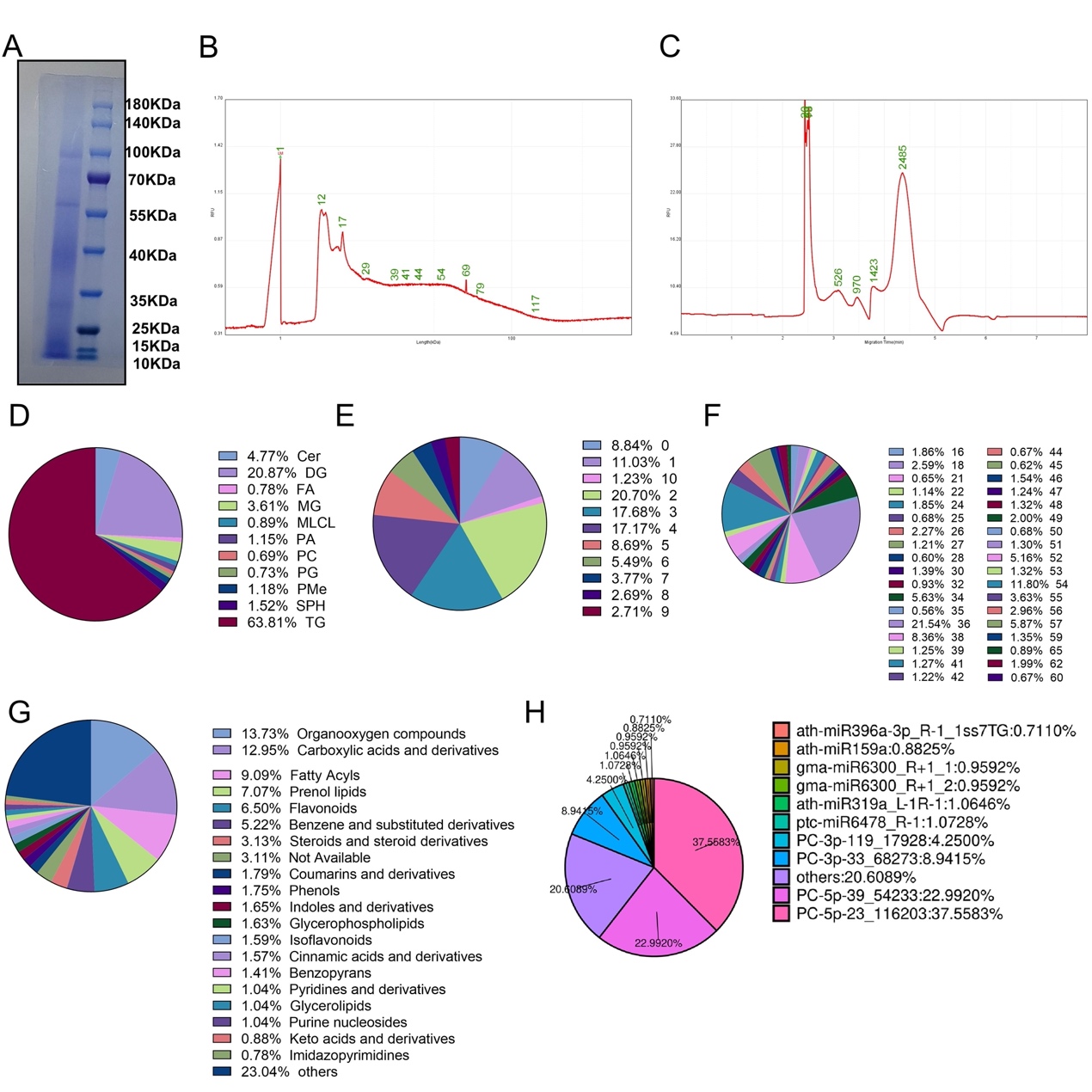


**Figure S2.** Characterization of honeysuckle-derived nanovesicles (HNVs). (A) Proteins of HNVs were separated with 10% sodium dodecyl sulfate polyacrylamide gel electrophoresis (SDS-PAGE) and stained with Coomassie blue. Proteins (B) and RNAs (C) of HNVs were separated according to size using the Bio-Fragment Analyzer. Lipidomic analysis of HNVs: (D) Proportion of each lipid component, (E) Proportion of carbon double bonds in each lipid, (F) Proportion of carbon chain length of each lipid. (G) Metabolomic analysis of HNVs. (H) miRNA sequencing analysis.
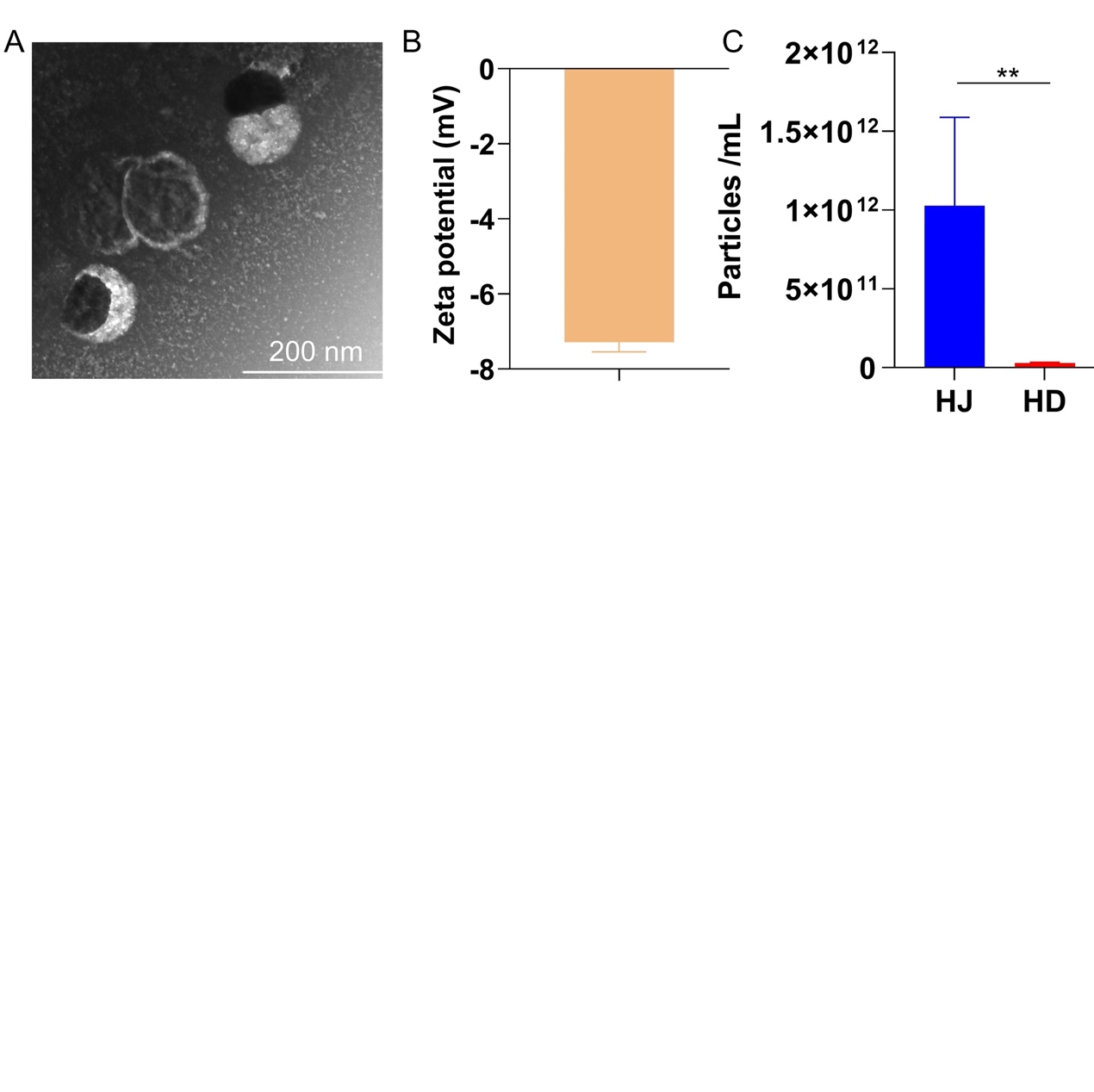


**Figure S3.** Identification and characterization of honeysuckle decoction exosome-like vesicles. (A) TEM imaging of exosome-like vesicles from honeysuckle decoction. (B) Zeta potential of exosome-like vesicles from honeysuckle decoction. (C) Counting of particles by nano tracking analysis (NTA). HJ, Honeysuckle juicer; HD, Honeysuckle decoction. Scale bar = 200 nm.


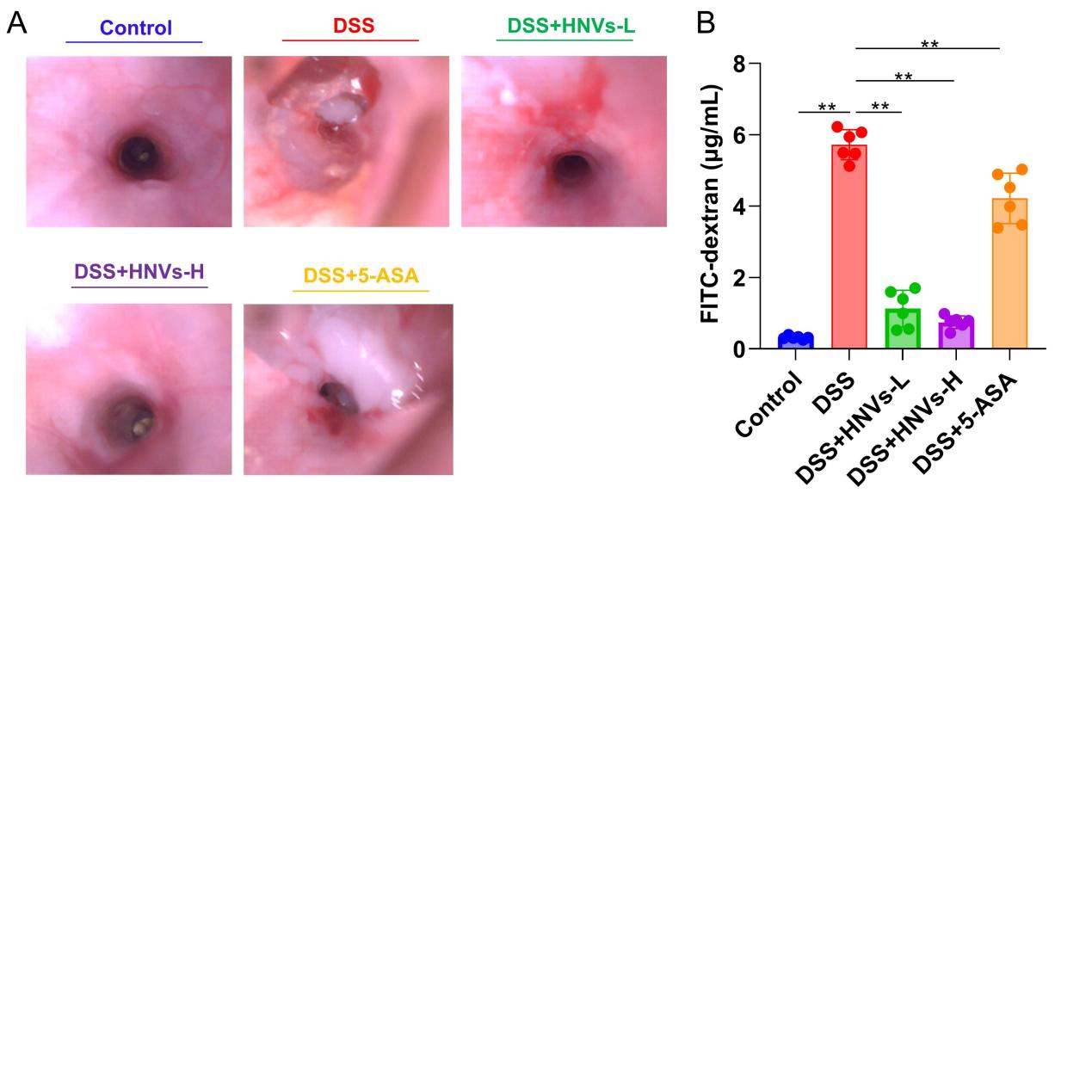


**Figure S4.** (A) Colonoscopy images. (B) Detecting intestinal permeability in mice using FITC-dextran. Data are presented as mean ± SD (n = 6), and P-values were determined using one-way ANOVA. *P < 0.05, **P < 0.01. ns, not significant.


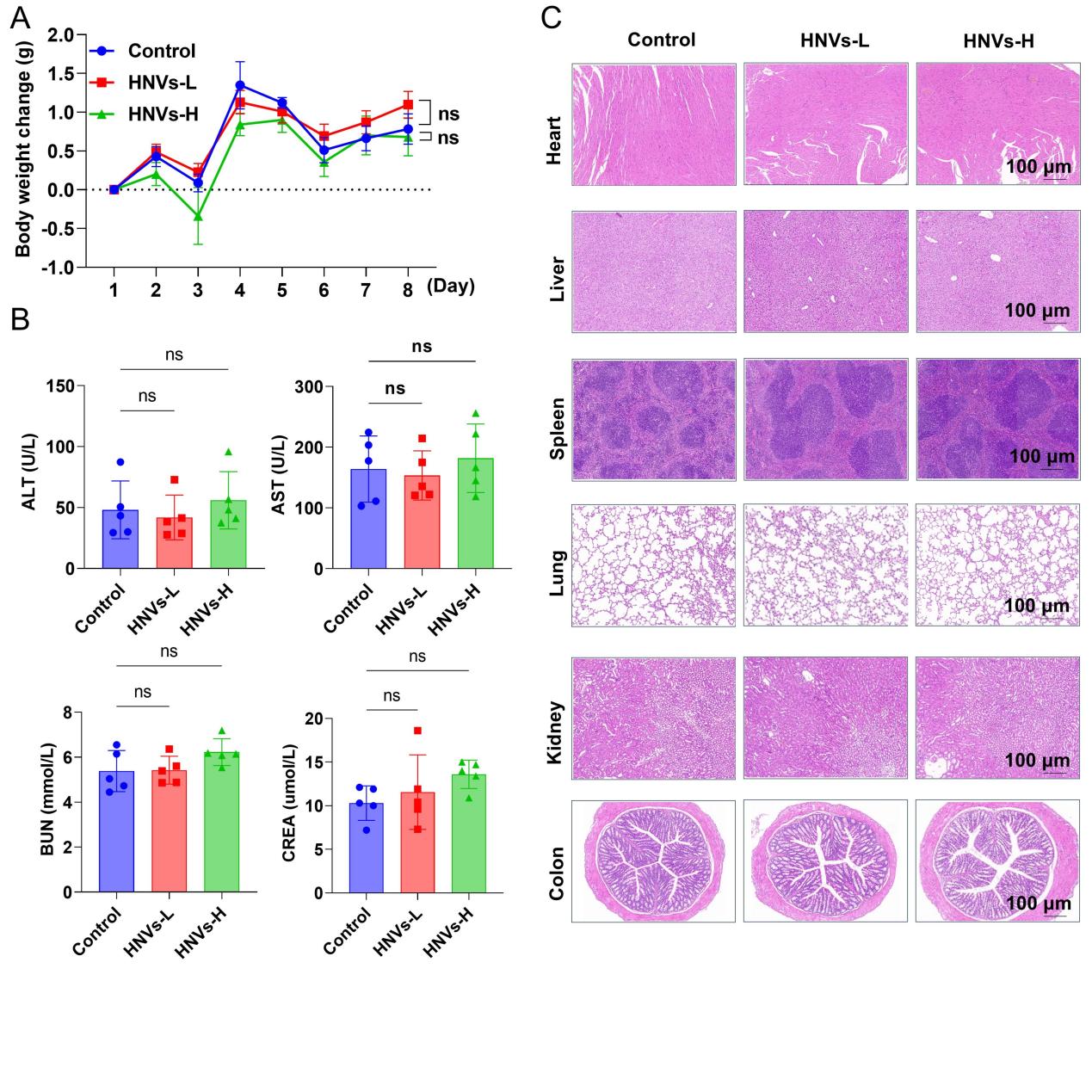


**Figure S5.** HNVs biomedical applications in mice. (A) Body weight gain over 8 days. (B) ALT, AST, CRAE, and BUN levels were determined in each group (n = 5). (C) Representative hematoxylin & eosin (HE) staining of the heart, liver, spleen, lungs, kidneys, and colon. Data are presented as mean ± SD (n = 5). ns, not significant. Scale bar = 100 μm.


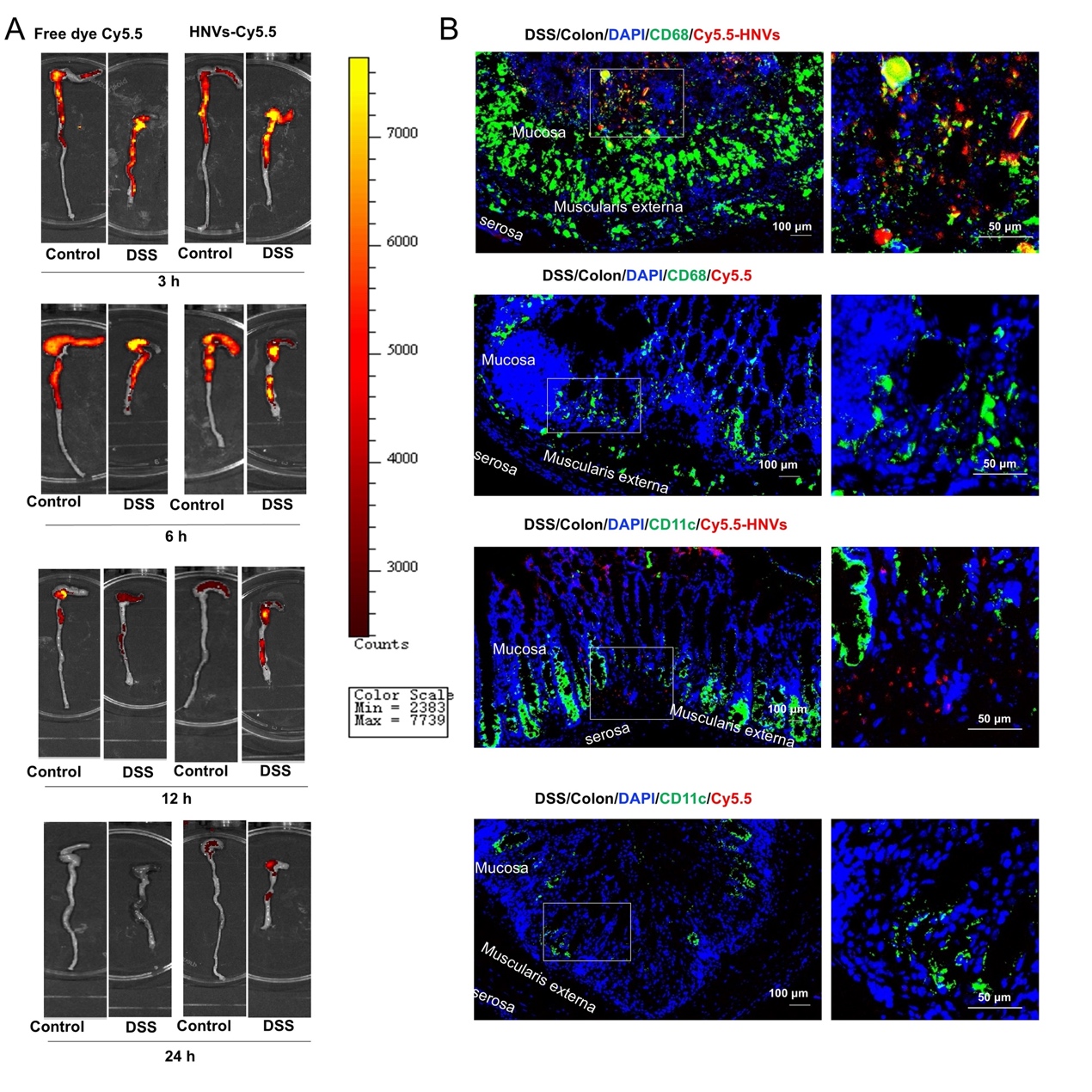


**Figure S6.** Biodistribution of HNVs in inflammatory bowel disease mice. The distribution of HNVs across various organs following oral administration was evaluated. (A) Fluorescent signals in the colon confirmed the accumulation of HNVs in the intestinal tract. (B) Fluorescence microscopy of colon cryosections showed Cy5.5-labeled HNVs (red) in close proximity to DC2.4 dendritic cells, stained with CD11c+ (green, upper panel), and macrophages, stained with CD68+ (green, lower panel). Colonic tissues were obtained from DSS-induced mice for analysis. Scale bar = 50 or 100 μm.


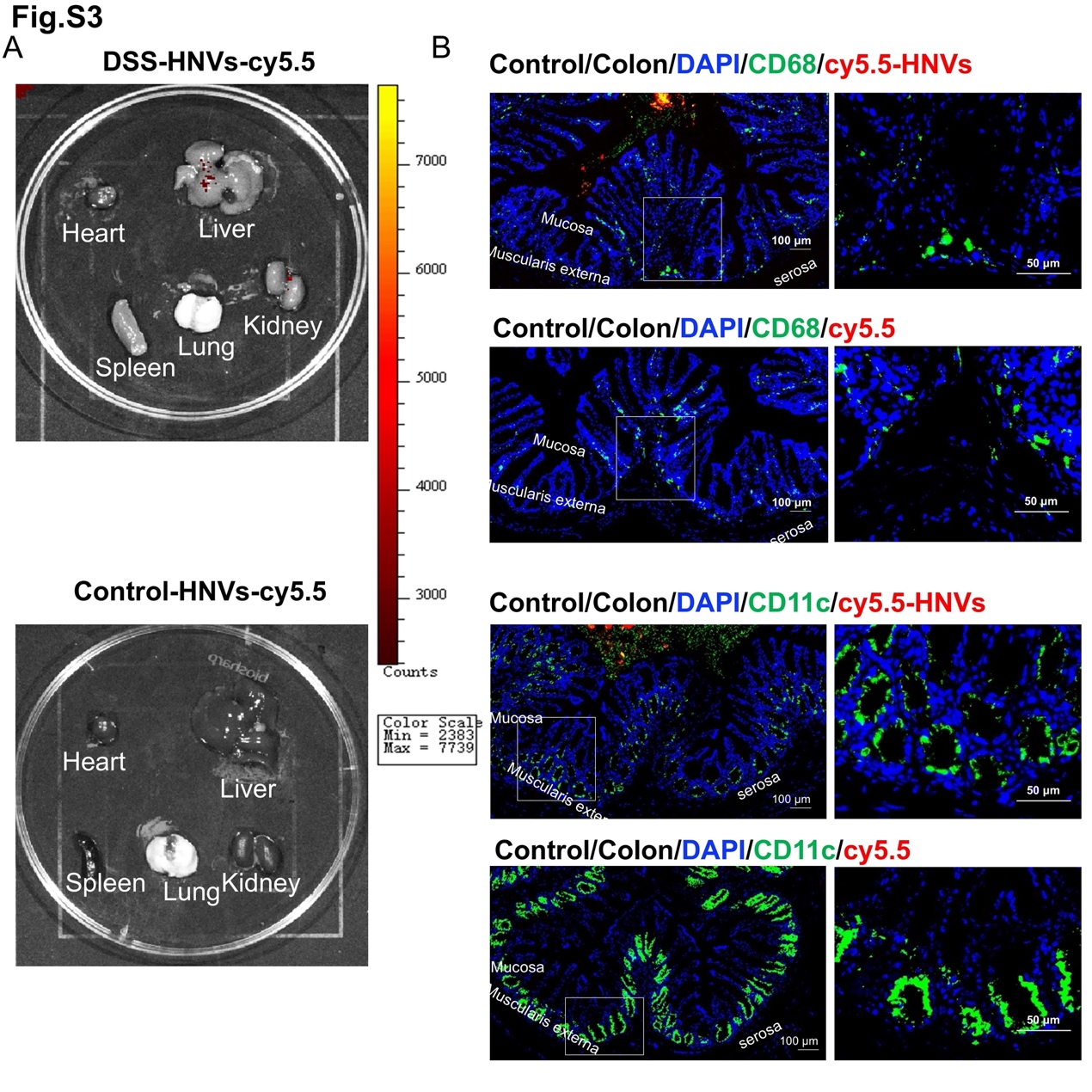


**Figure S7.** Biodistribution of HNVs in healthy mice. The distribution of HNVs across various organs following oral administration was evaluated. (A) Fluorescent signals were observed in the heart, liver, spleen, lungs, and kidneys, indicating the systemic distribution of HNVs. (B) Fluorescence microscopy of colon cryosections showed Cy5.5-labeled HNVs (red) in close proximity to DC2.4 dendritic cells, stained with CD11c+ (green, upper panel), and macrophages, stained with CD68+ (green, lower panel). Scale bar = 50 or 100 μm.


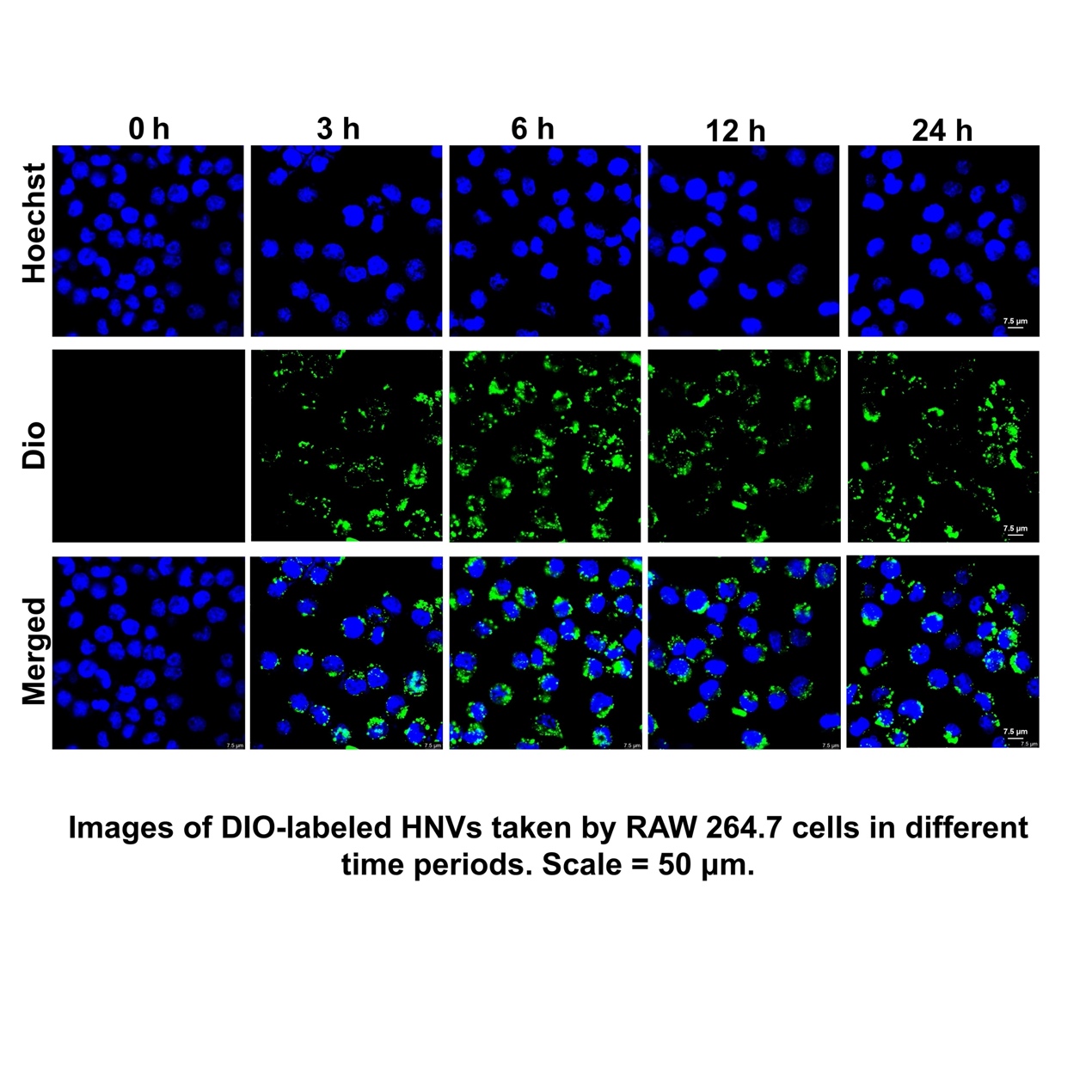


**Figure S8.** Images of Dio-labeled HNVs taken by RAW264.7 cells in different time periods. Scale bar = 7.5 μm.

**
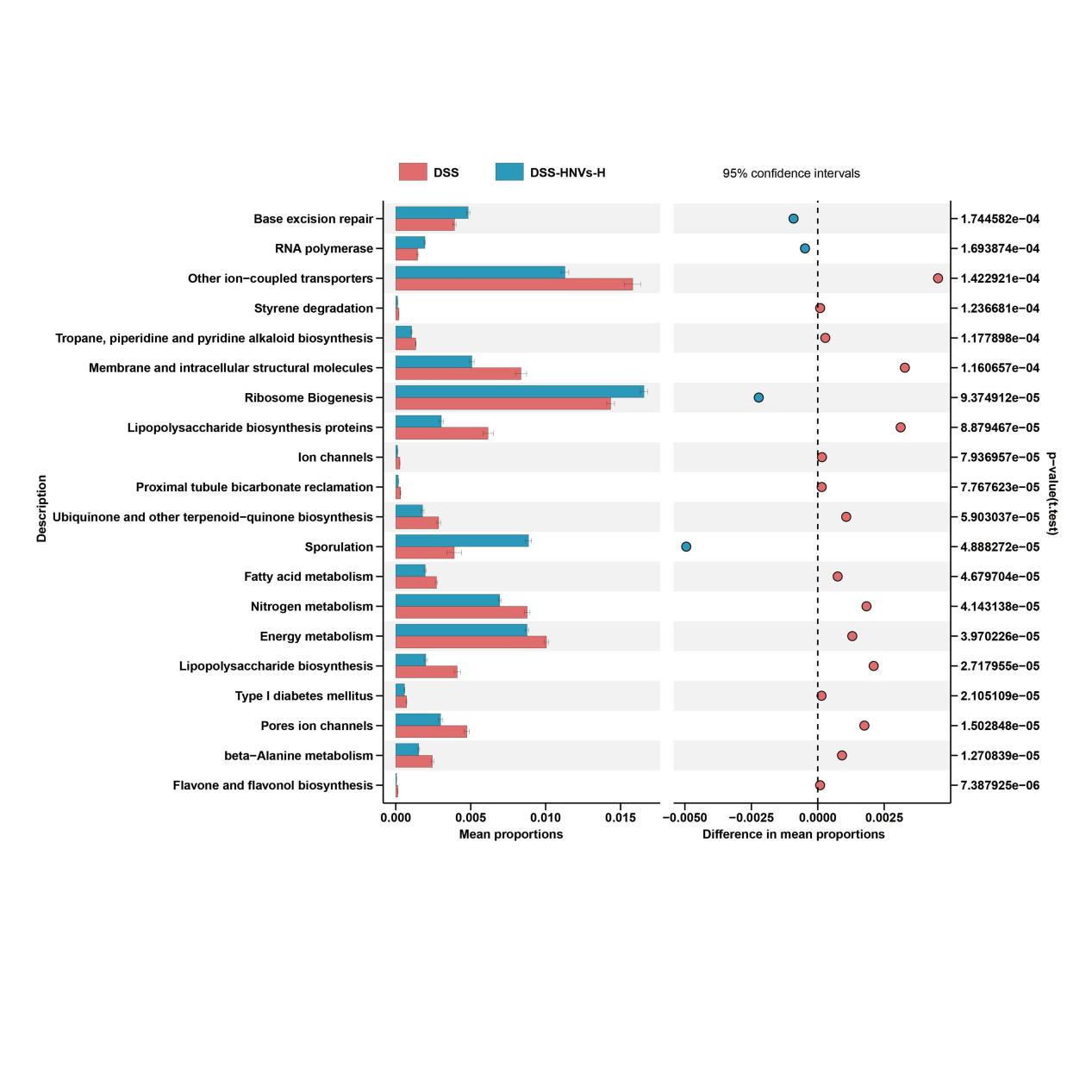
**

**Figure S9.** Effects of HNVs treatment on the gut microbiota structure in DSS-induced experimental IBD in C57BL/6 mice. PICRUSt2 functional prediction: STAMP analysis results presented in the upper panel focus on the top 20 functions with statistically significant t-test differences (P < 0.05). The figure displays functionally significant differences (with a 95% confidence interval), allowing initial speculation on the association between microbiota and the relevant functions.


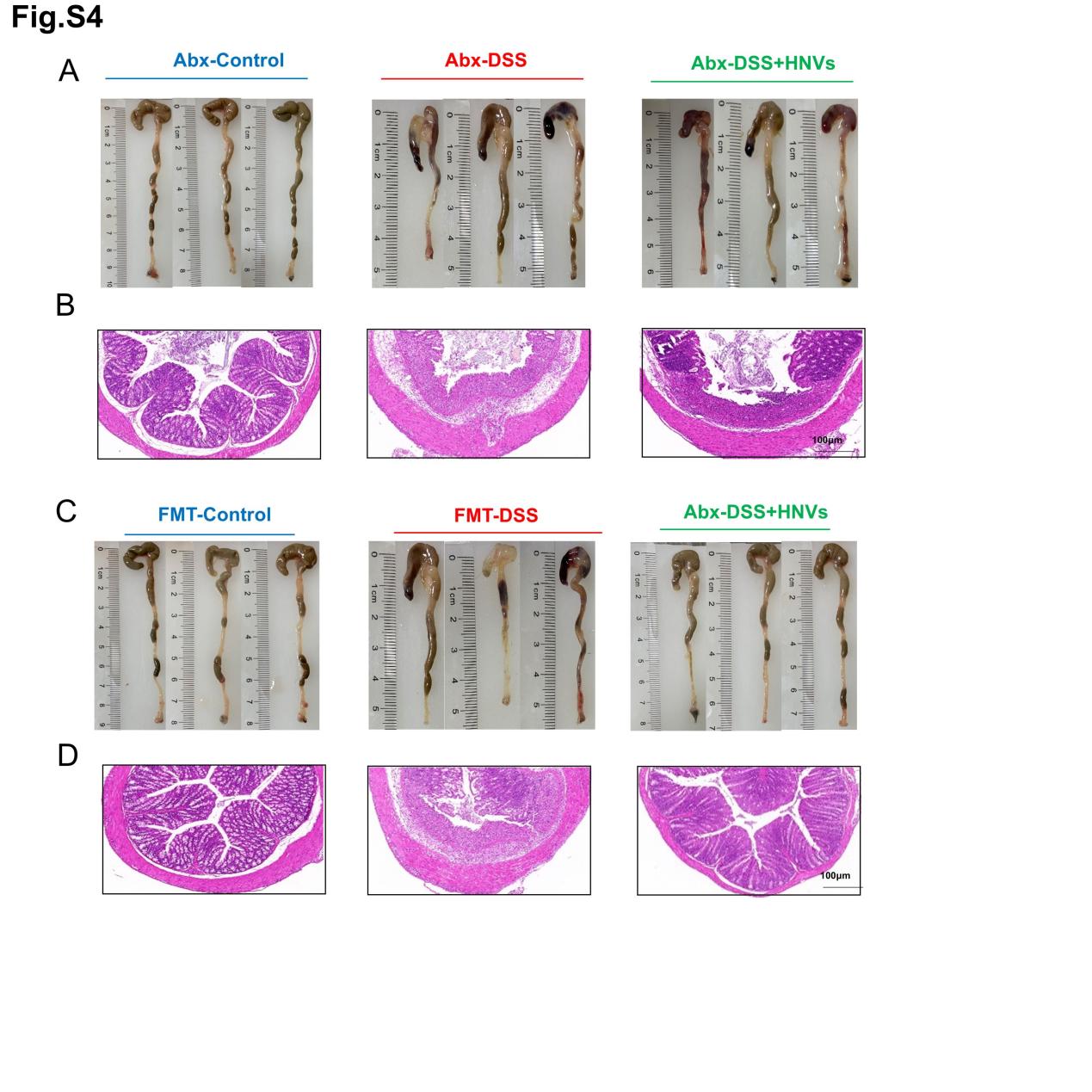


**Figure S10.** Effects of HNVs treatment on DSS-induced experimental IBD symptoms in C57BL/6 mice with depleted gut microbiota. Colon photograph and colonic tissues by HE staining in C57BL/6 mice with depleted gut microbiota and fecal microbiota transplantation mice, respectively. Scale bar = 100 μm.


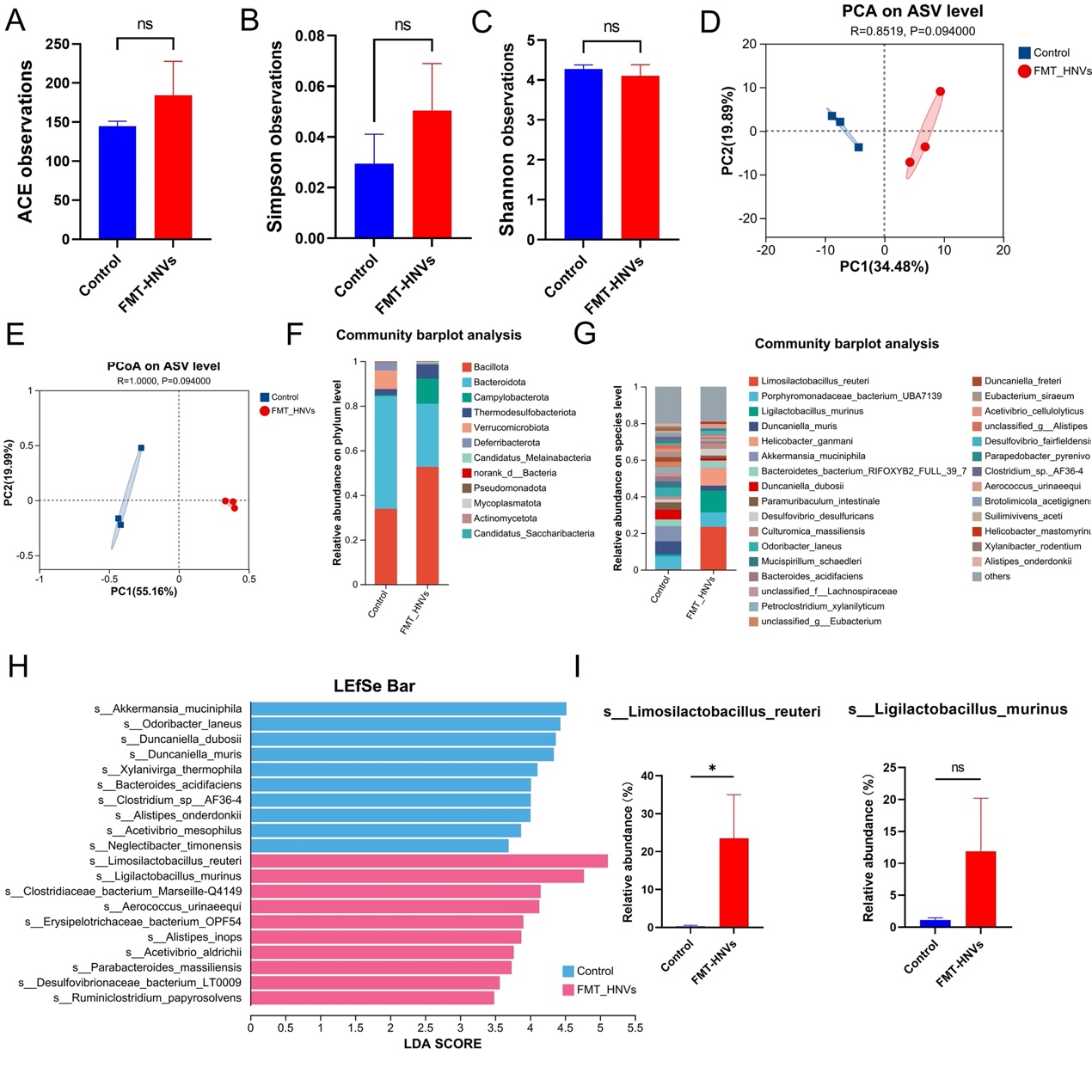


**Figure S11.** The effect of HNVs on the gut microbiota structure of fecal microbiota transplantation donor mice. Based on the observed operational classification units, evaluate microbial community alpha diversity using (A) ACE index, (B) Simpson index, and (C) Shannon index. (D) Principal component and (D) principal coordinate analysis demonstrate the beta diversity analysis of gut microbiota composition. (E) The relative abundance of microbial composition at the phylum level (F) and species level (G). (H) LEfSe multi-level species hierarchy bar chart. (G) Bar chart of relative abundance of different microorganisms in fecal microbiota transplantation suspension. The data are presented as mean ± standard deviation (n = 3), and the P-value was determined using the T-test of variance. *P < 0.05.
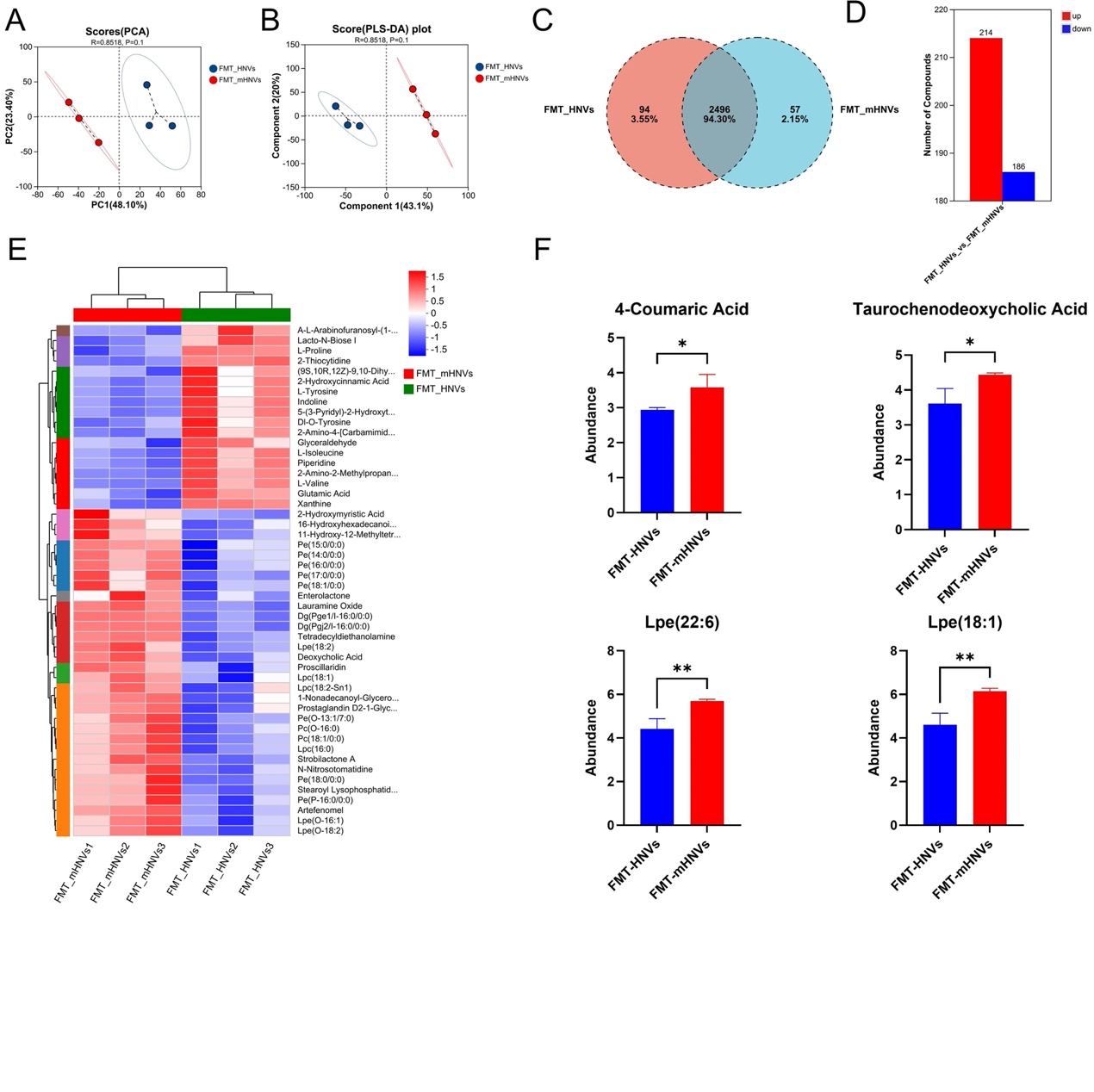


**Figure S12.** Non-targeted metabolic detection of fecal microbiota transplantation suspension. (A) Principal component analysis (PCA). (B) Partial least squares discriminant analysis (PLS-DA). (C) Statistical analysis of differentially expressed genes with (C) Venn diagrams and (D) bar charts. (E) Cluster analysis of differentially expressed genes. (F) Bar chart of differential metabolites in fecal microbiota transplantation suspension. Data are presented as mean ± SD (n = 3), and P-values were determined using the T-test. *P < 0.05, **P < 0.01.
