# Supplementary material for: Honeysuckle‐Derived Nanovesicles Regulate Gut Microbiota for the Treatment of Inflammatory Bowel Disease
Source: Adv Sci (Weinh). 2025 Sep 19;12(45):e05208. doi: 10.1002/advs.202505208 (PMC12677632; doi:10.1002/advs.202505208)
Supplement: Supplementary file 2 — Supporting Information [file ADVS-12-e05208-s001.zip › Supplementary_Images.docx]

Figure 2 H

Control：


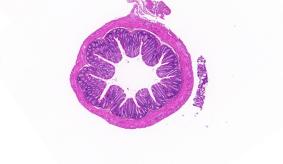

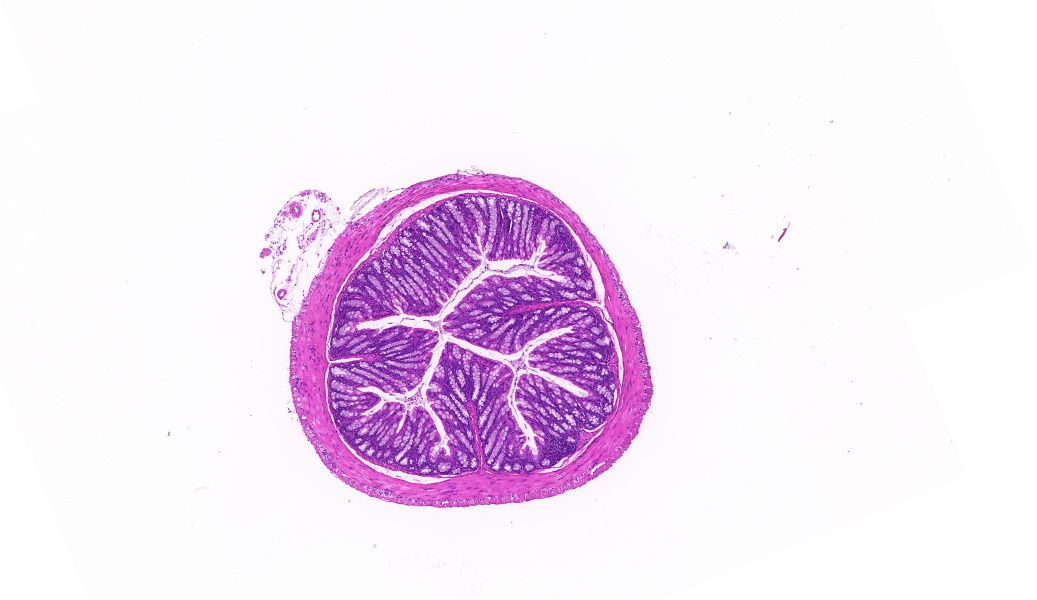

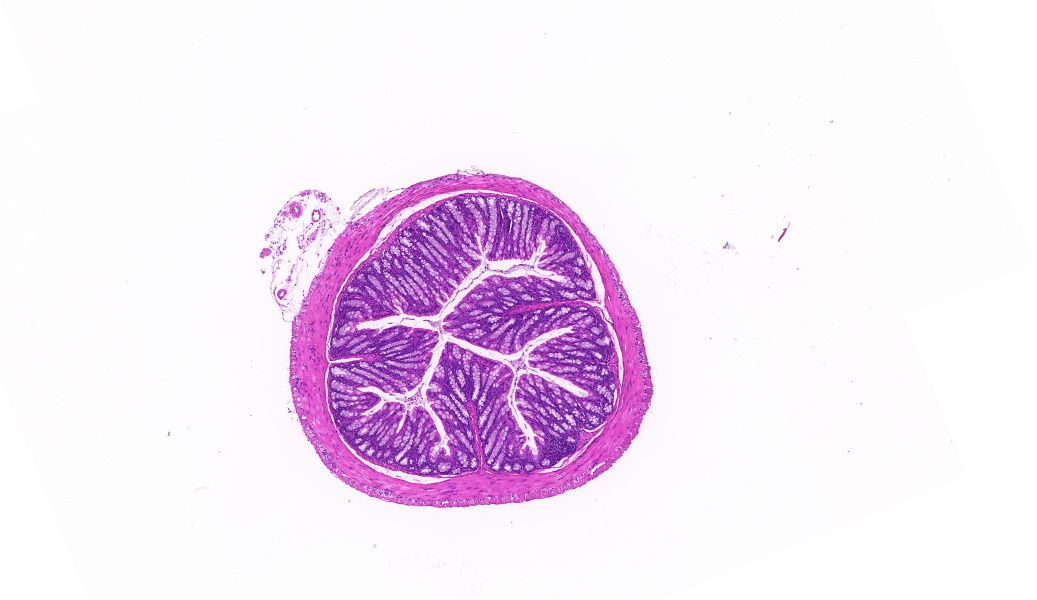

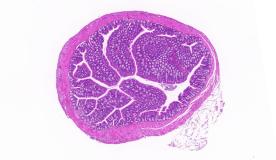

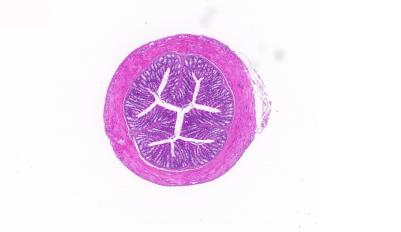

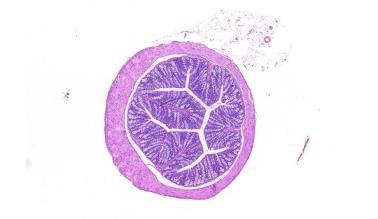


DSS:


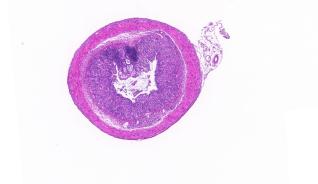

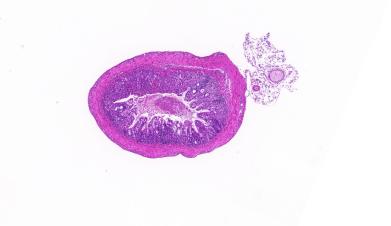

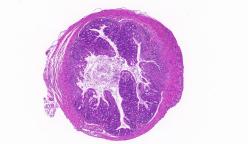

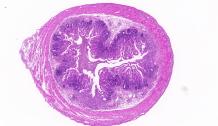

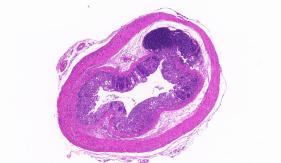

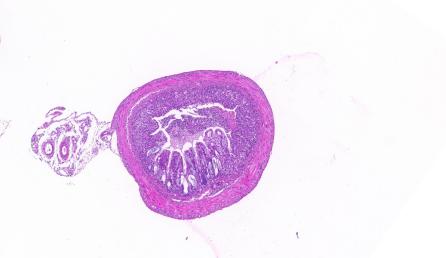


DSS+HNVs-L:


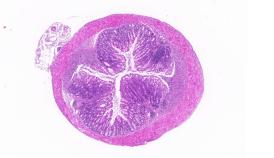

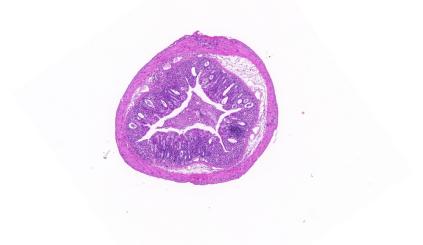

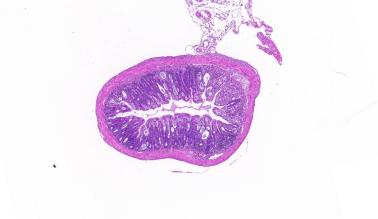

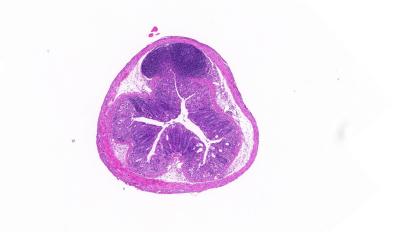

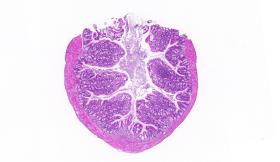

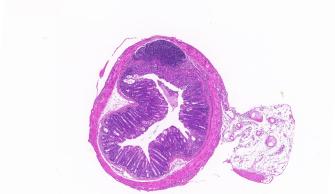


DSS+HNVs-H:


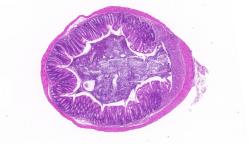

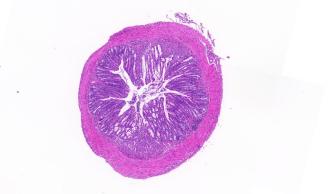

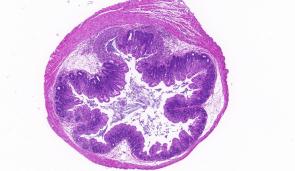

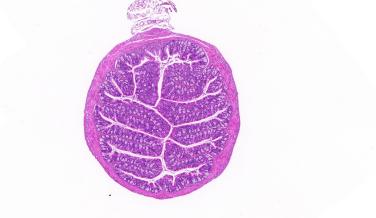

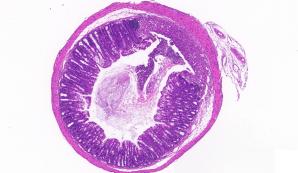

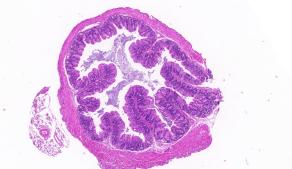


DSS+5-ASA:


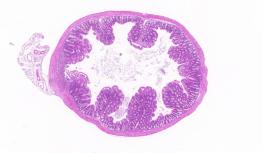

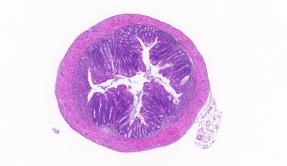

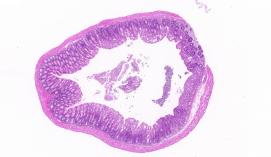

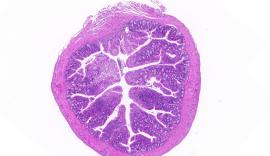

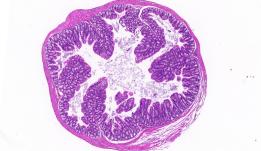

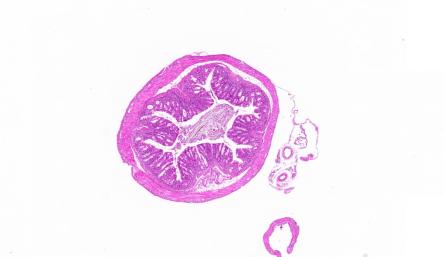


DSS+HD:


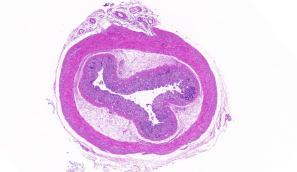

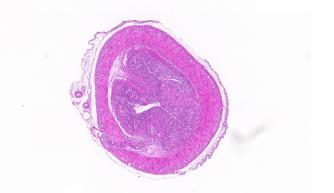

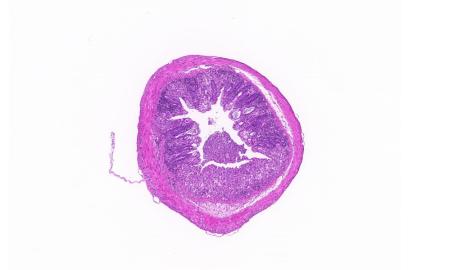

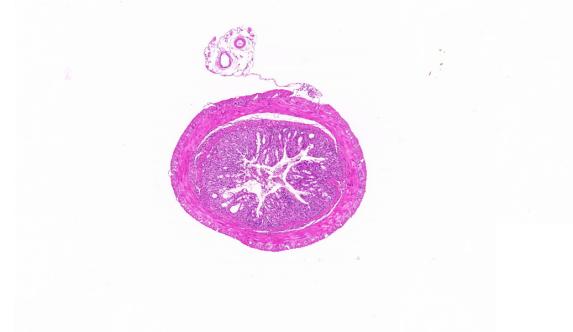

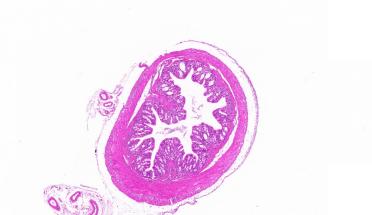

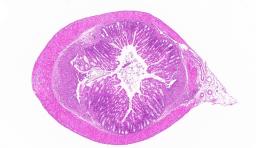


DSS+HJ:


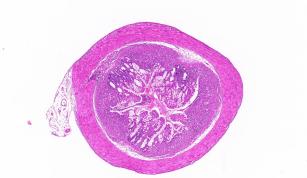

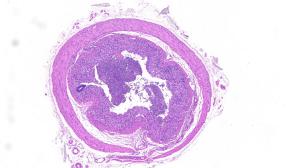

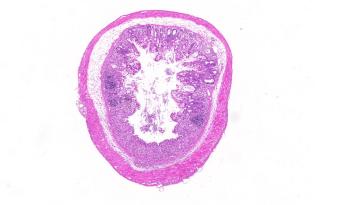

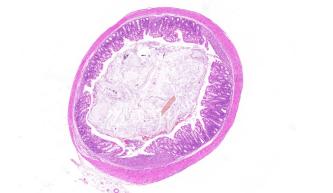

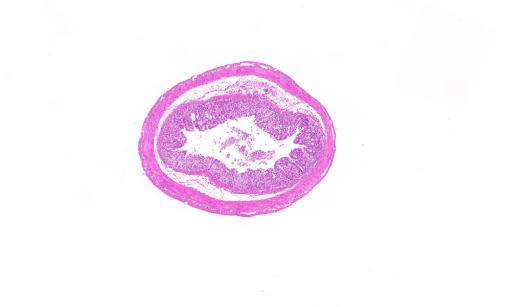

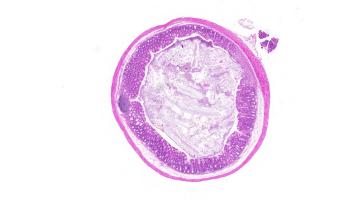


Figure 3A

Control -CD3^+^:


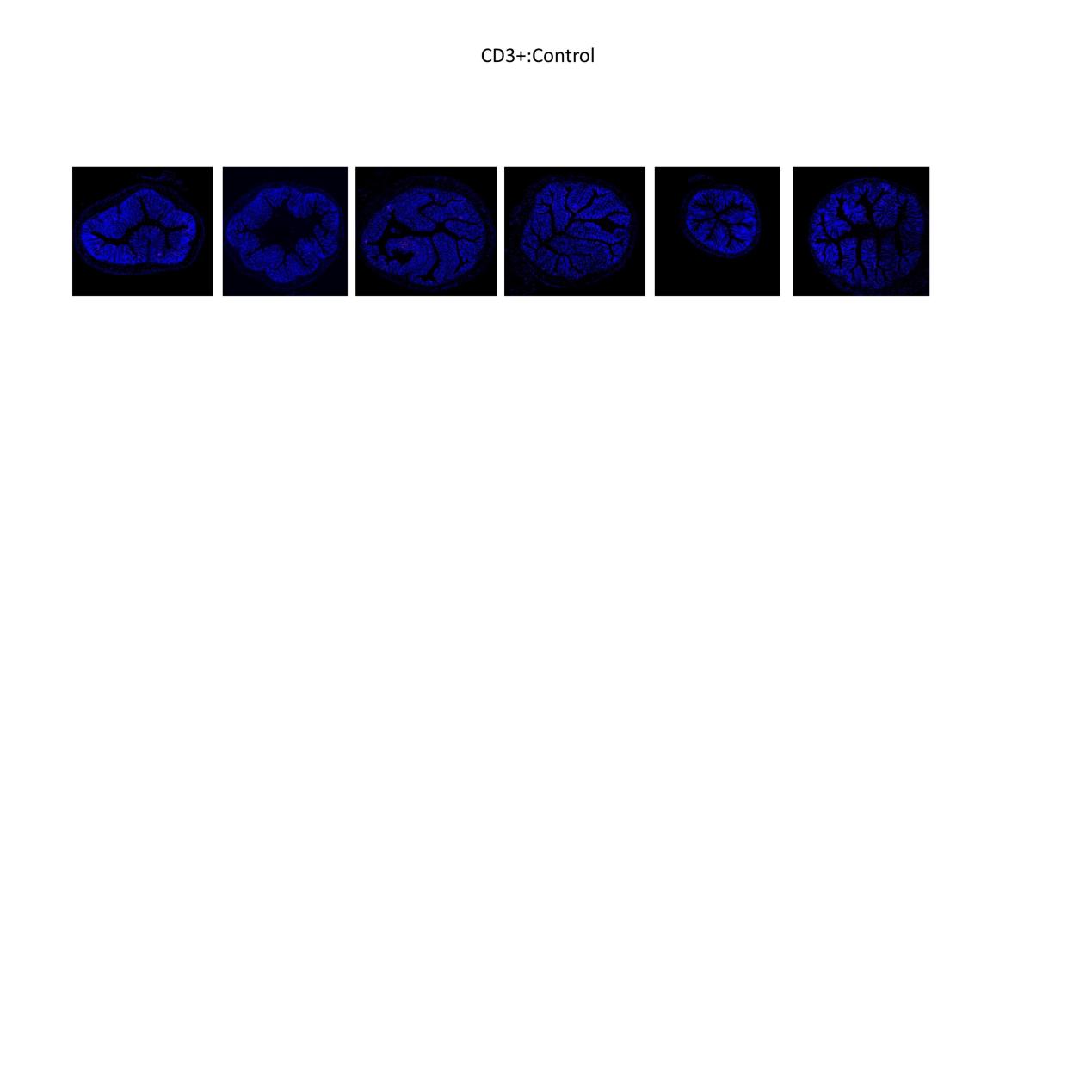


DSS -CD3^+^:


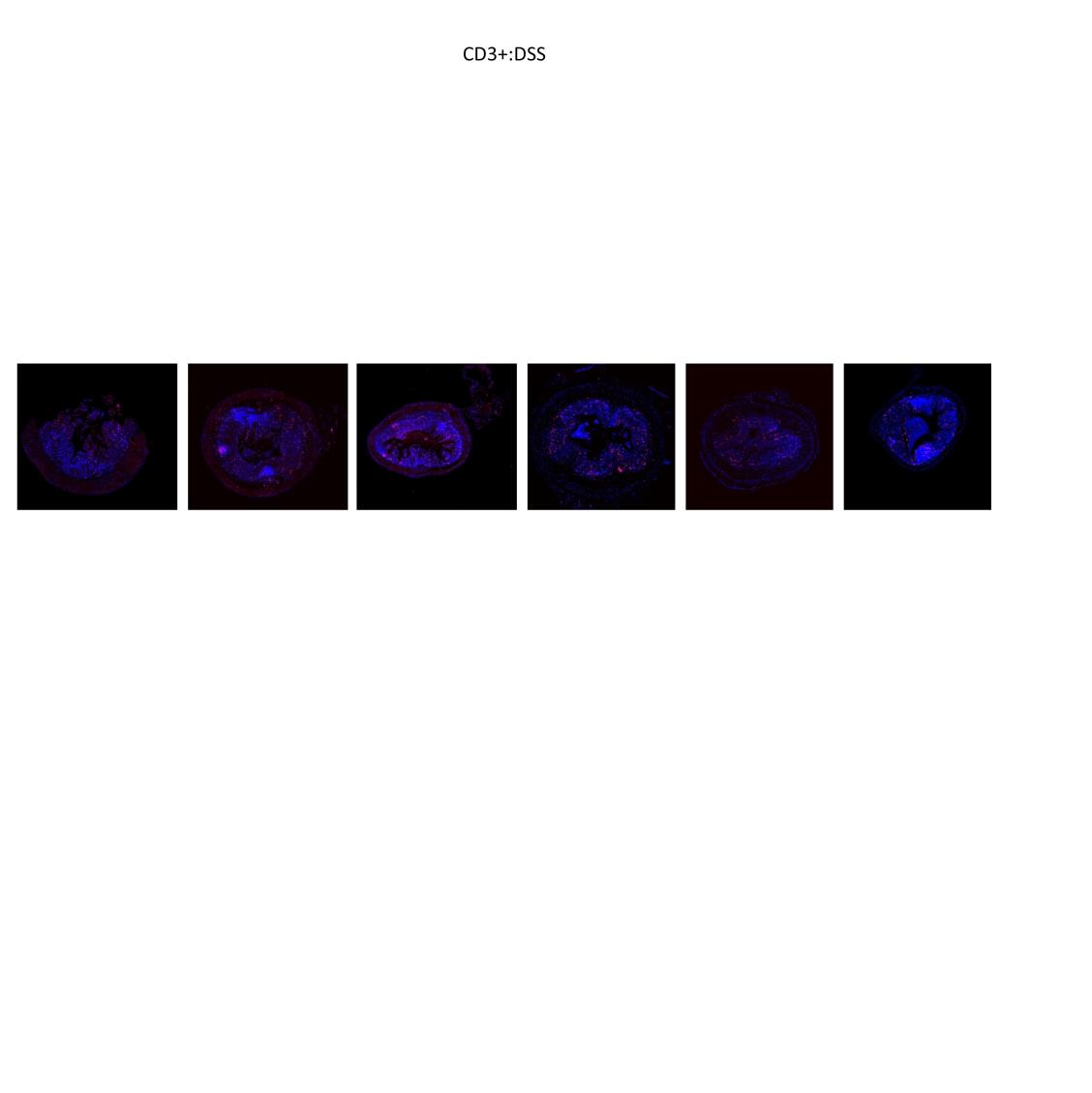


DSS+HNVs-L-CD3^+^:


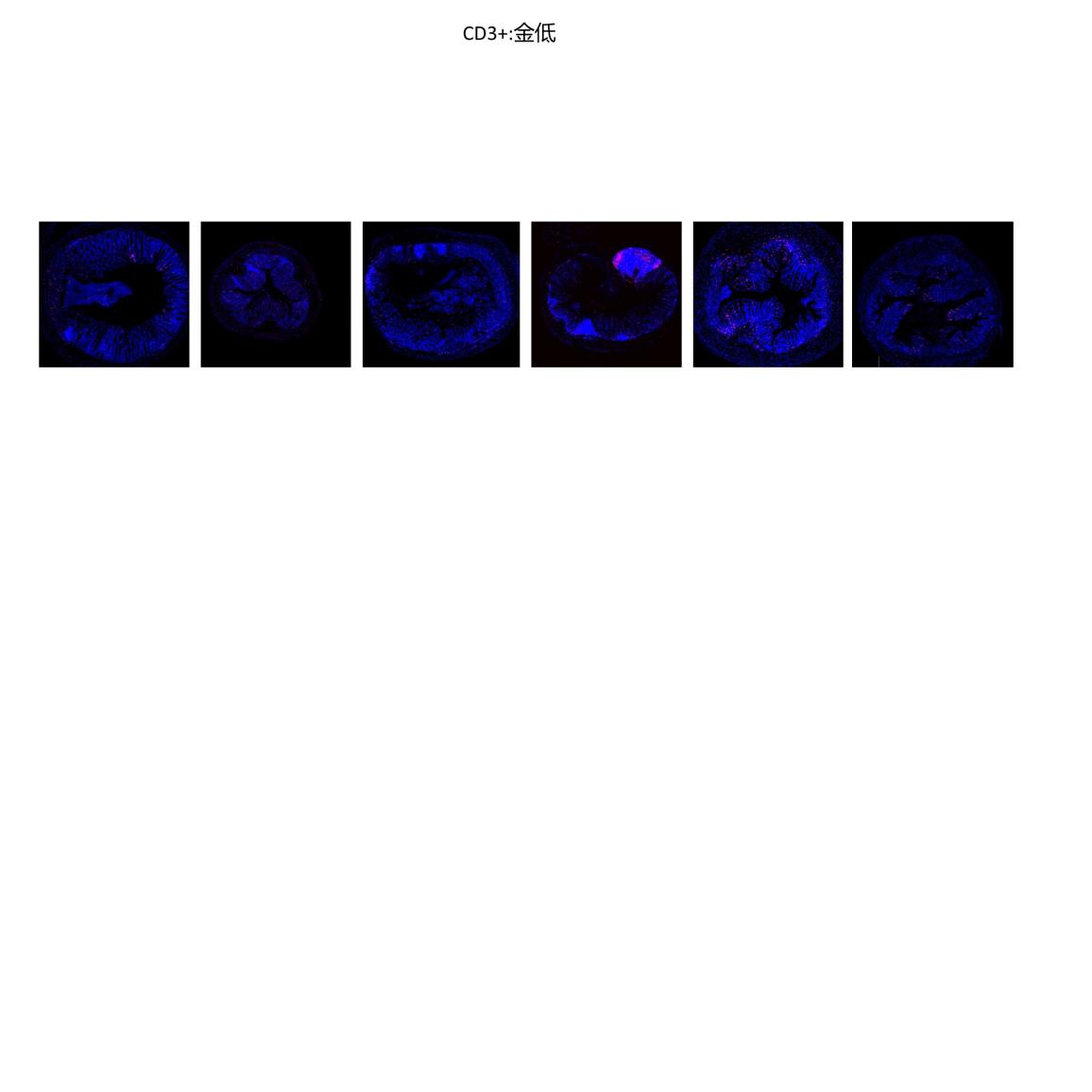


DSS+HNVs-H-CD3^+^:


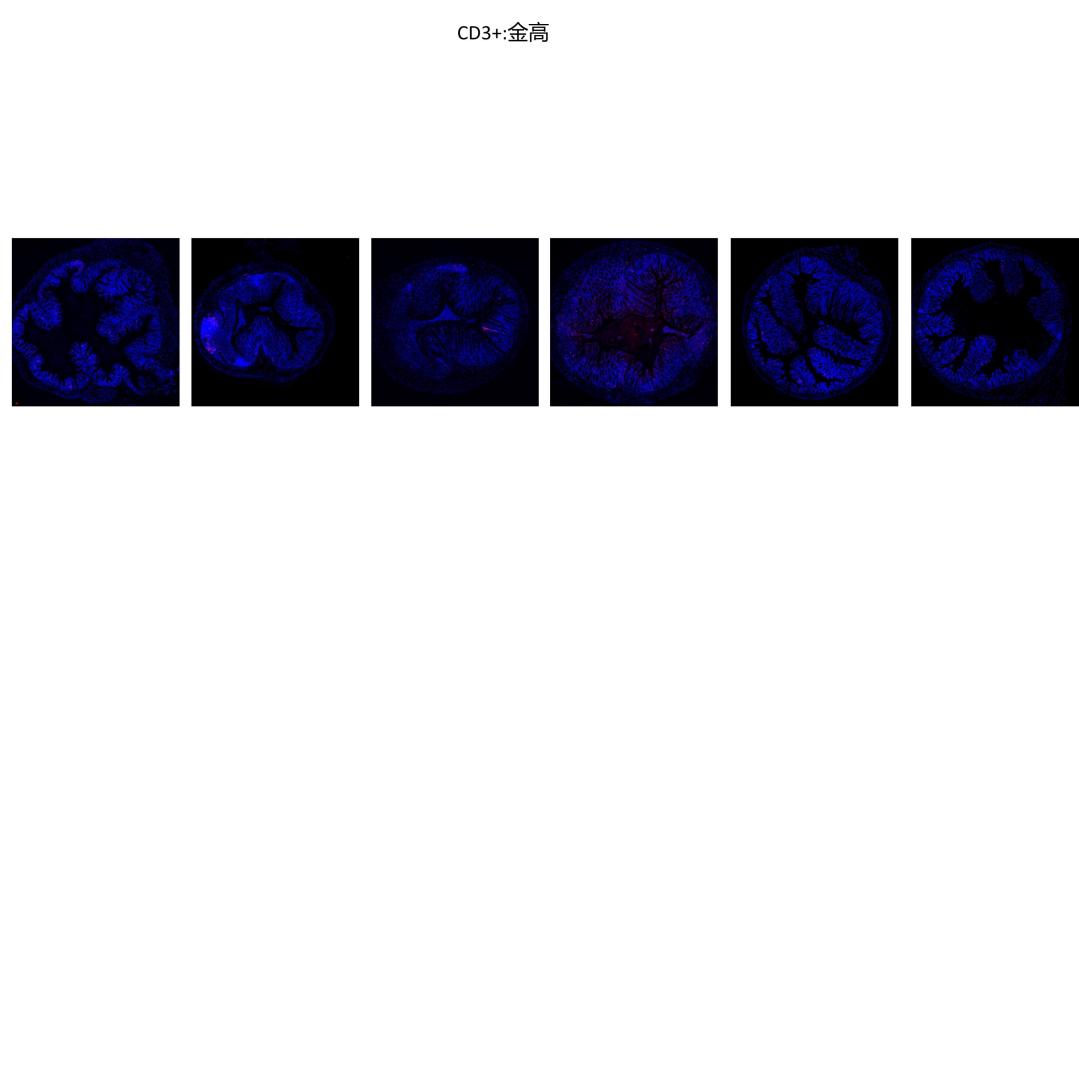


DSS+5-ASA-CD3^+^:


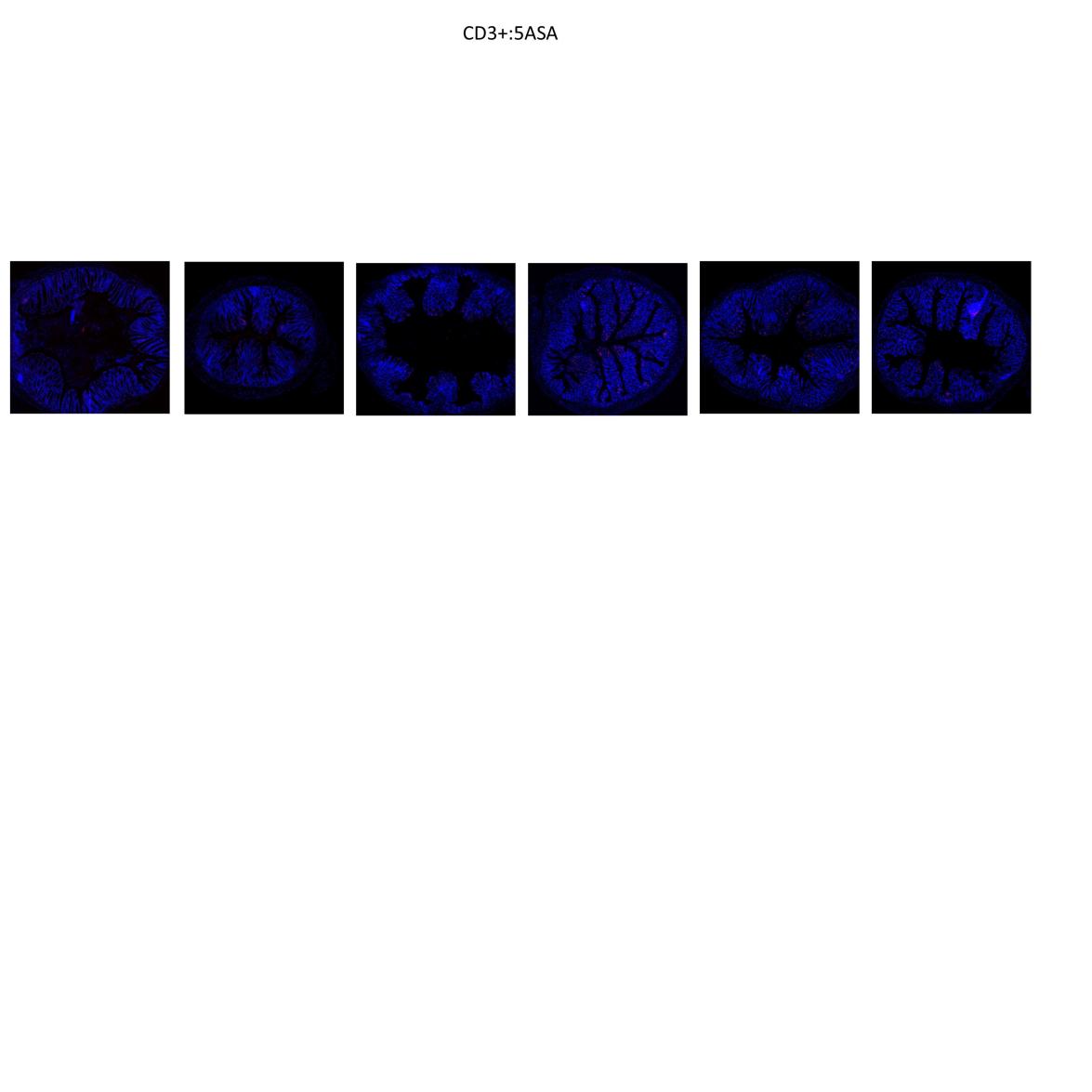


Control -CD8^+^:


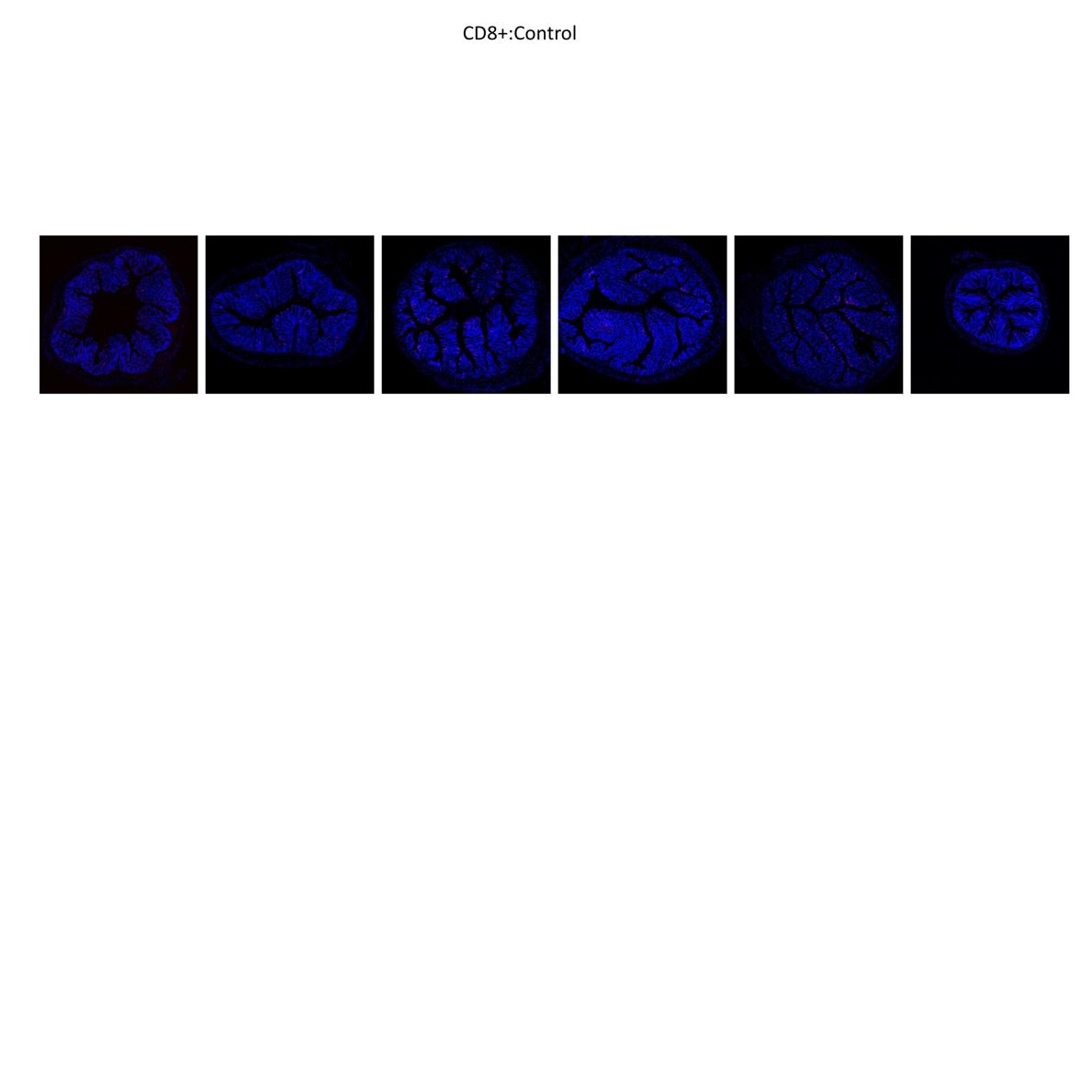


DSS -CD8^+^:


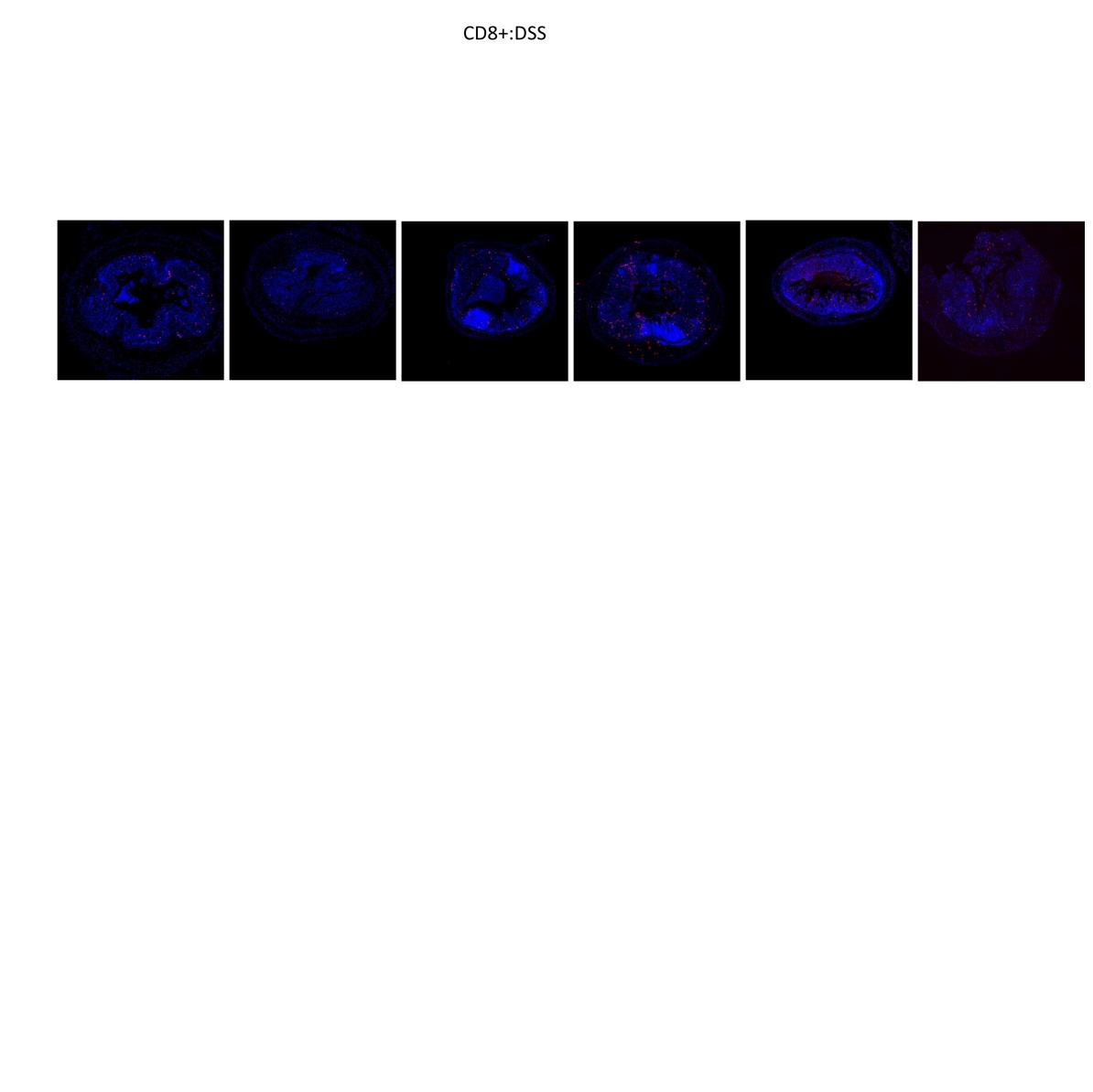


DSS+HNVs-L -CD8^+^:


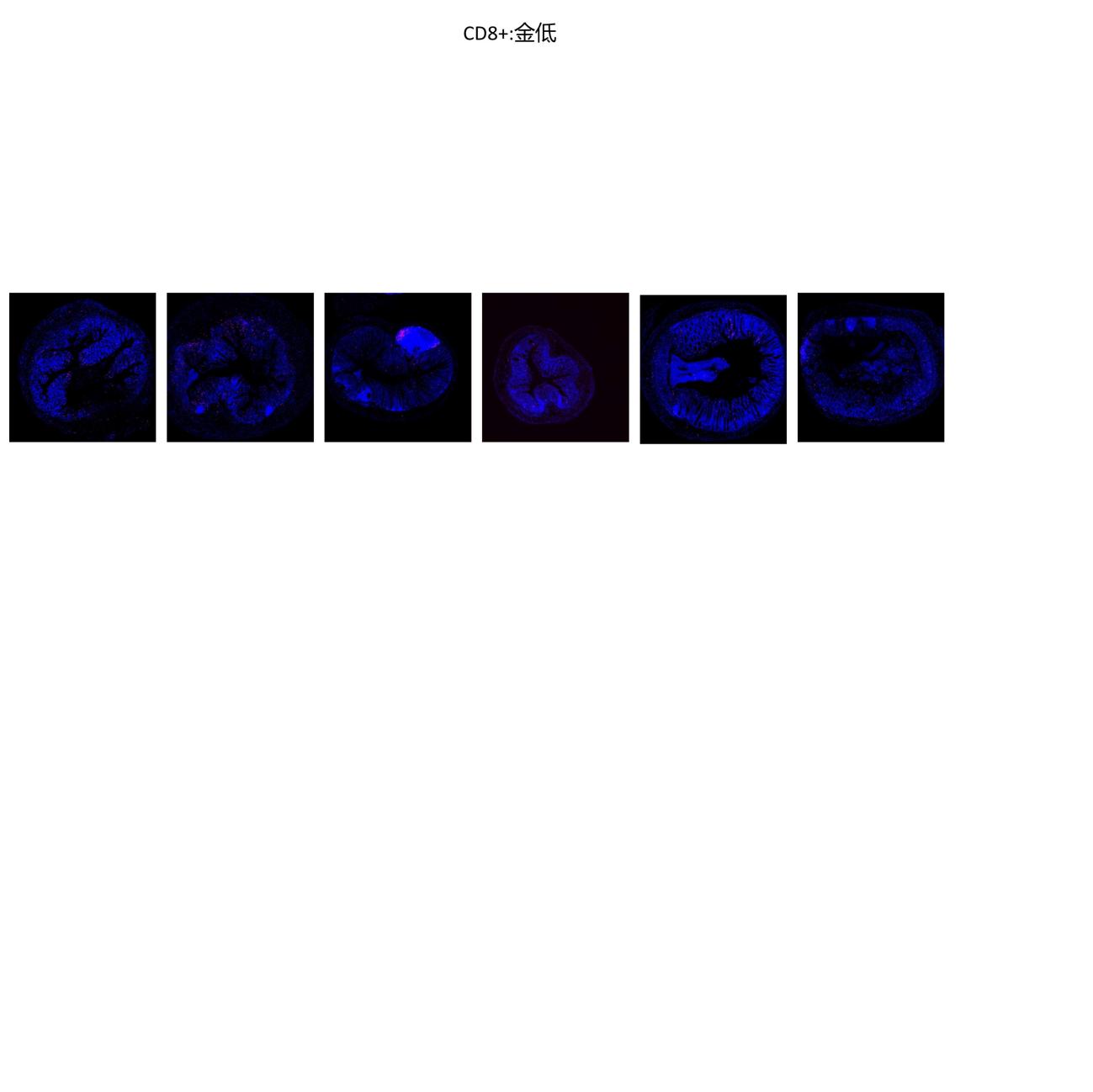


DSS+HNVs-H -CD8^+^:


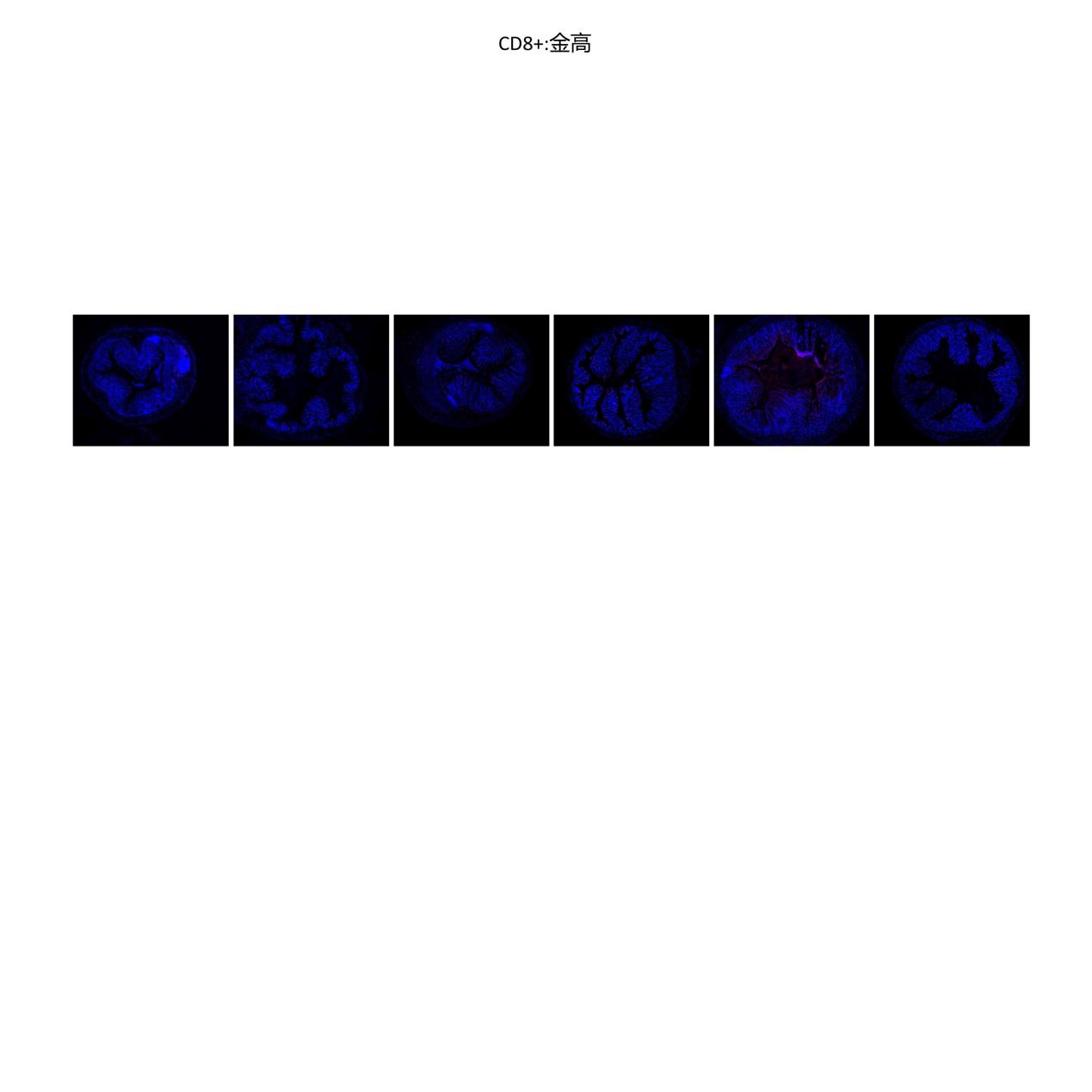


DSS+5-ASA -CD8^+^:


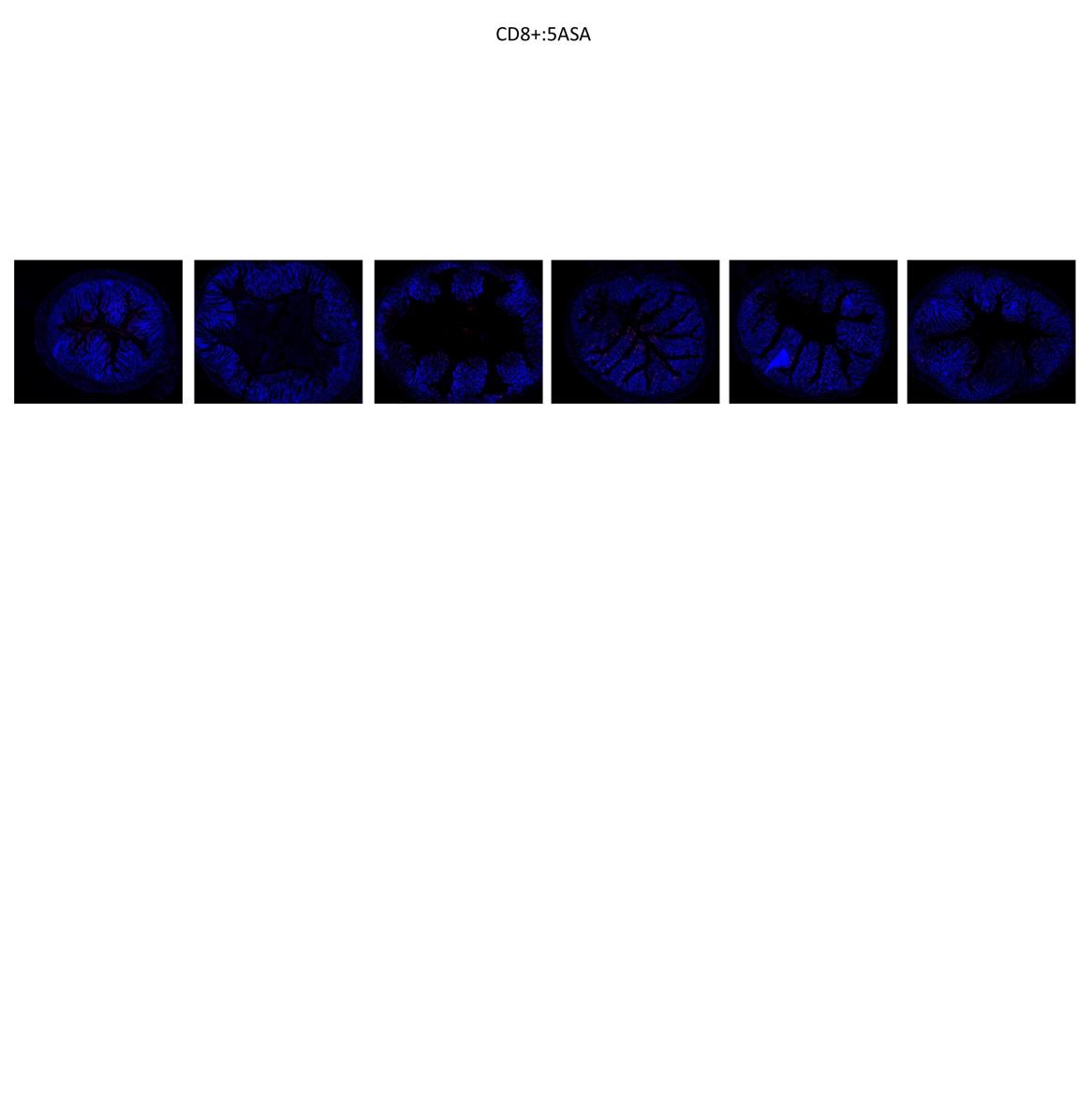


Control-M_2_Mø :


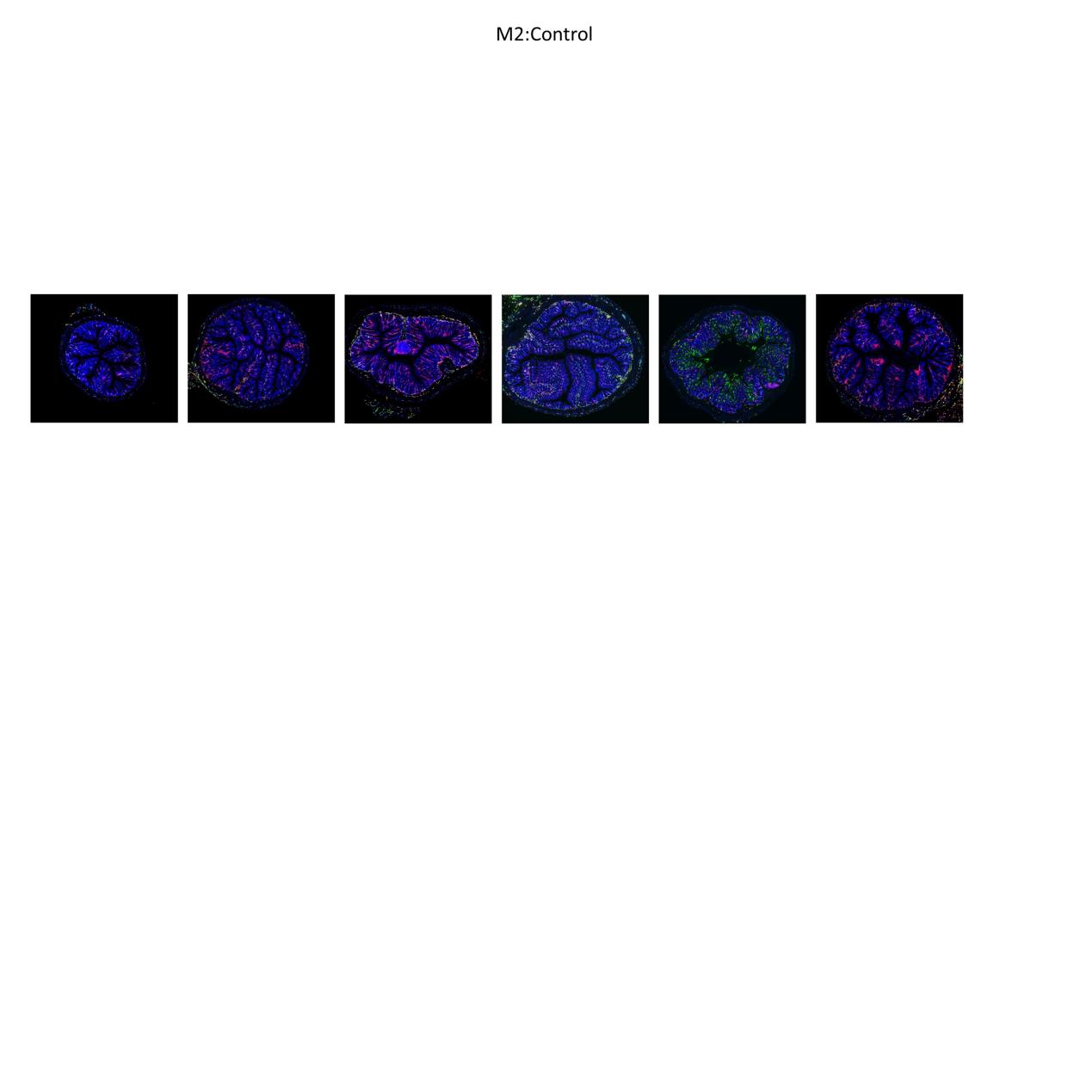


DSS-M_2_Mø :


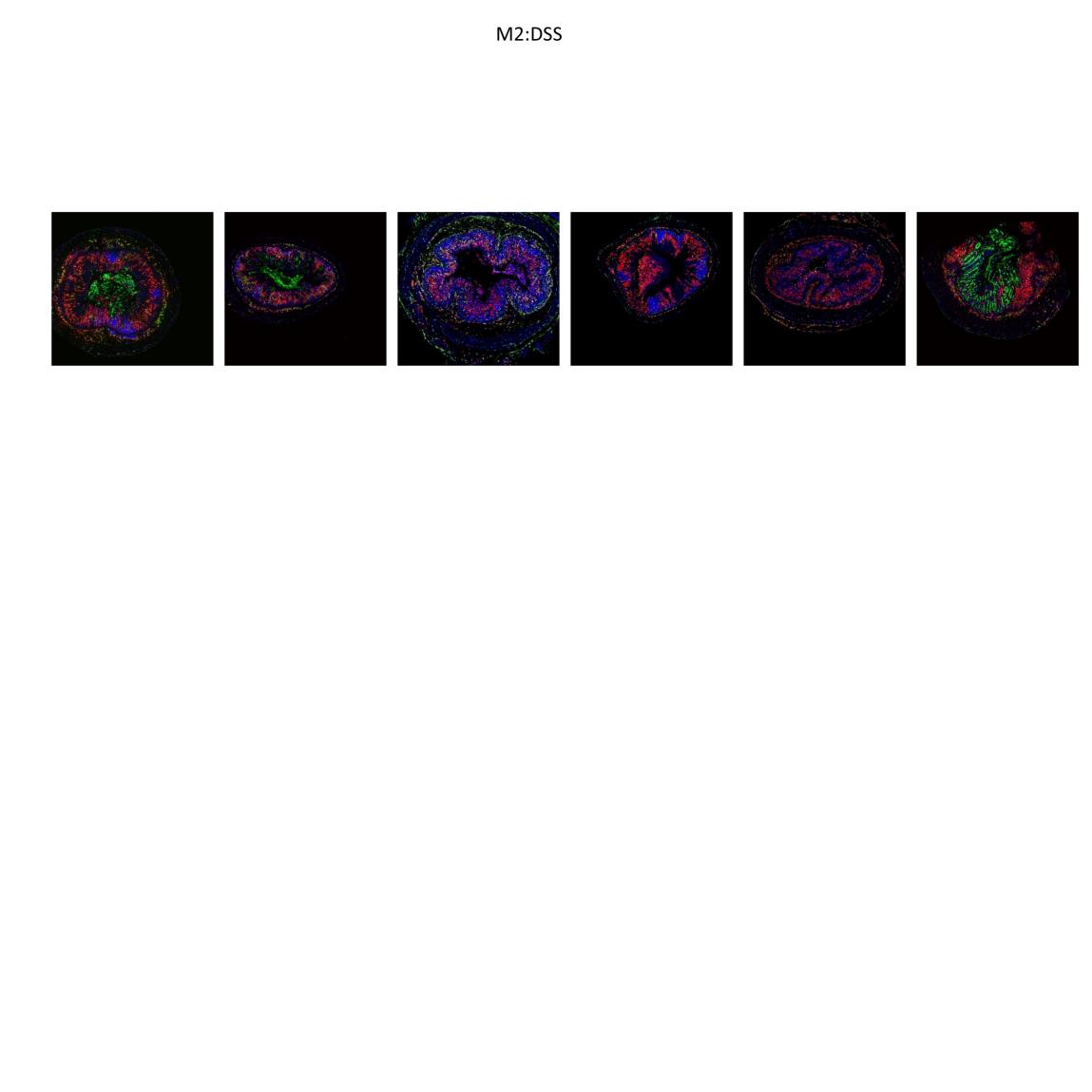


DSS+HNVs-L-M_2_Mø :


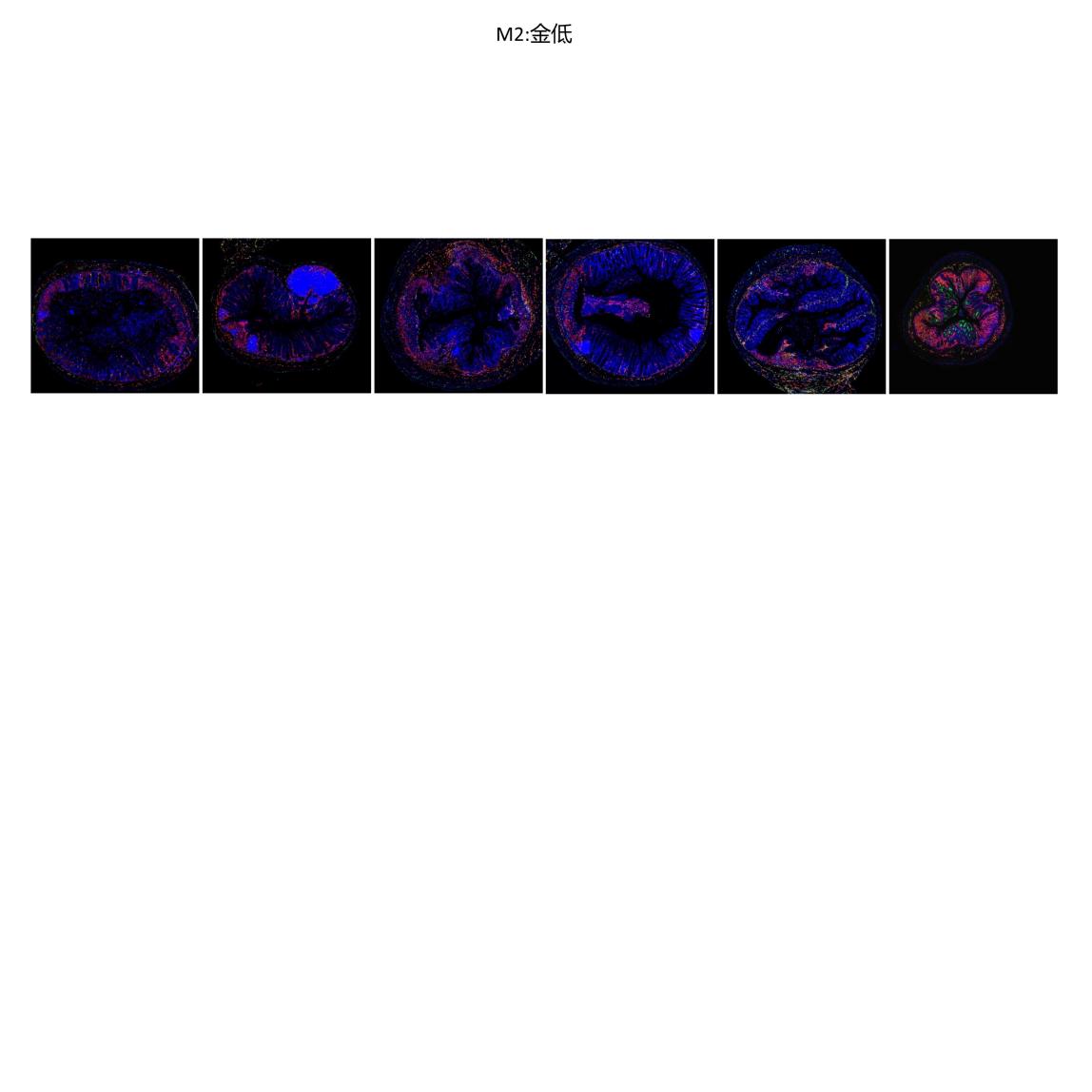


DSS+HNVs-H-M_2_Mø :


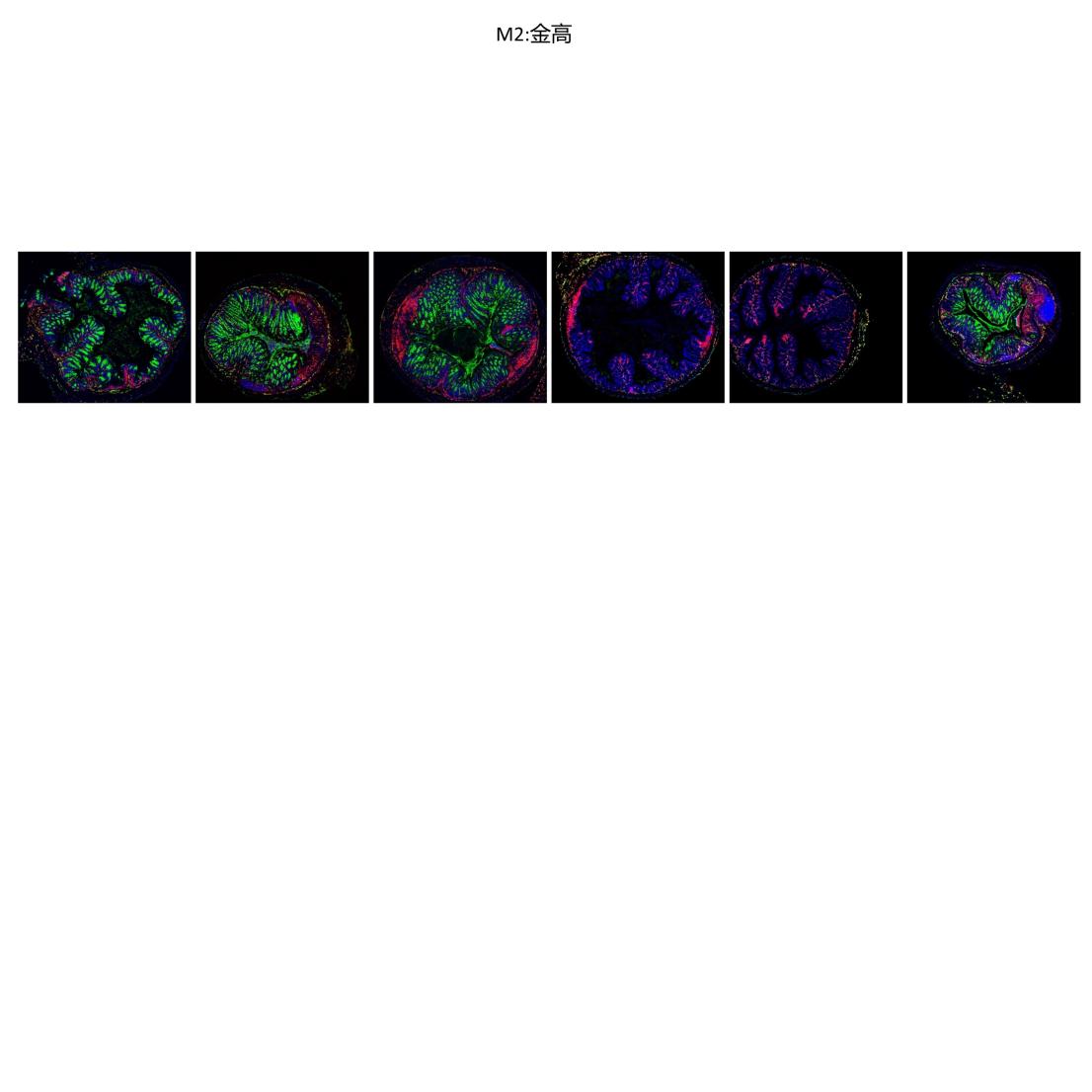


DSS+5-ASA-M_2_Mø :


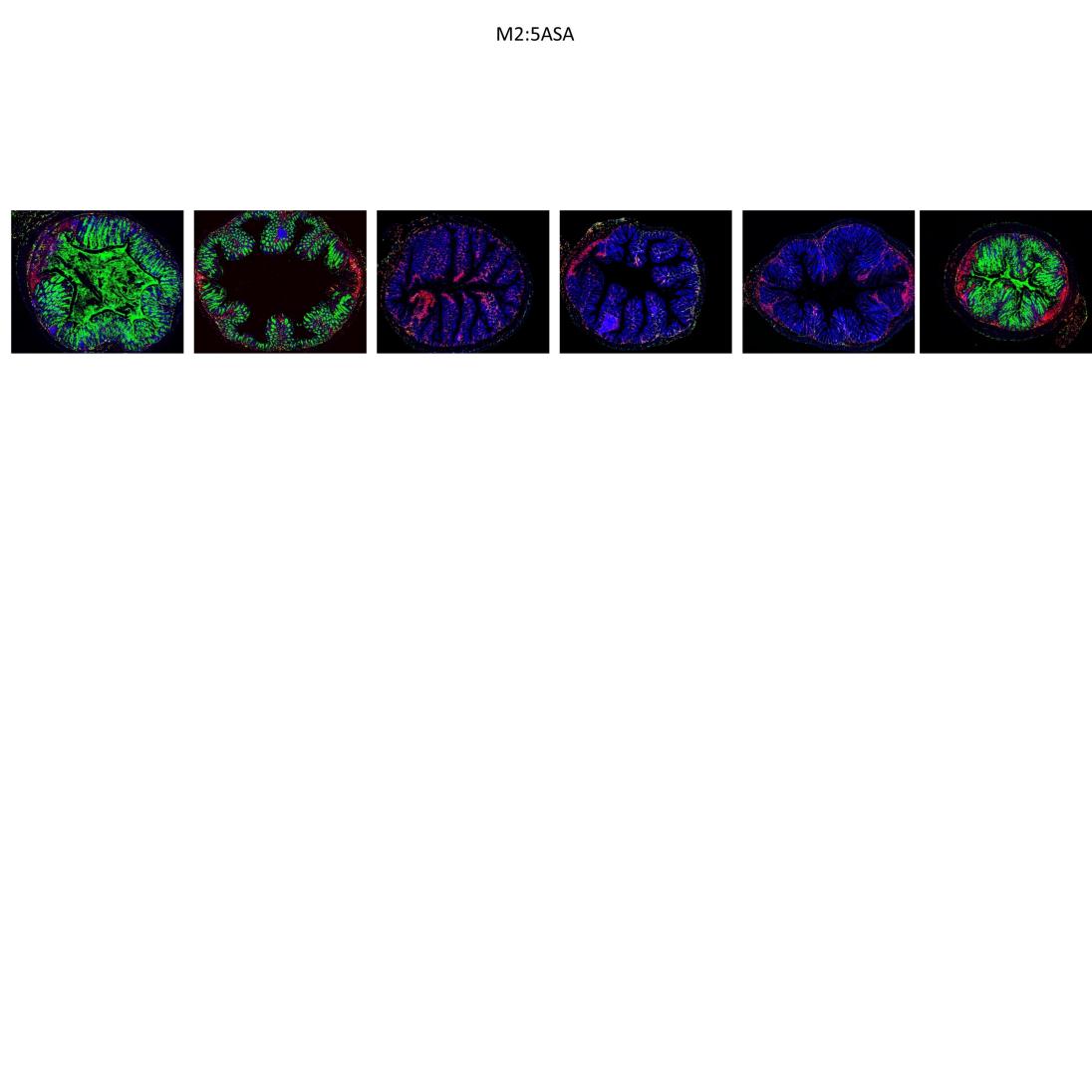


Control-MPO :


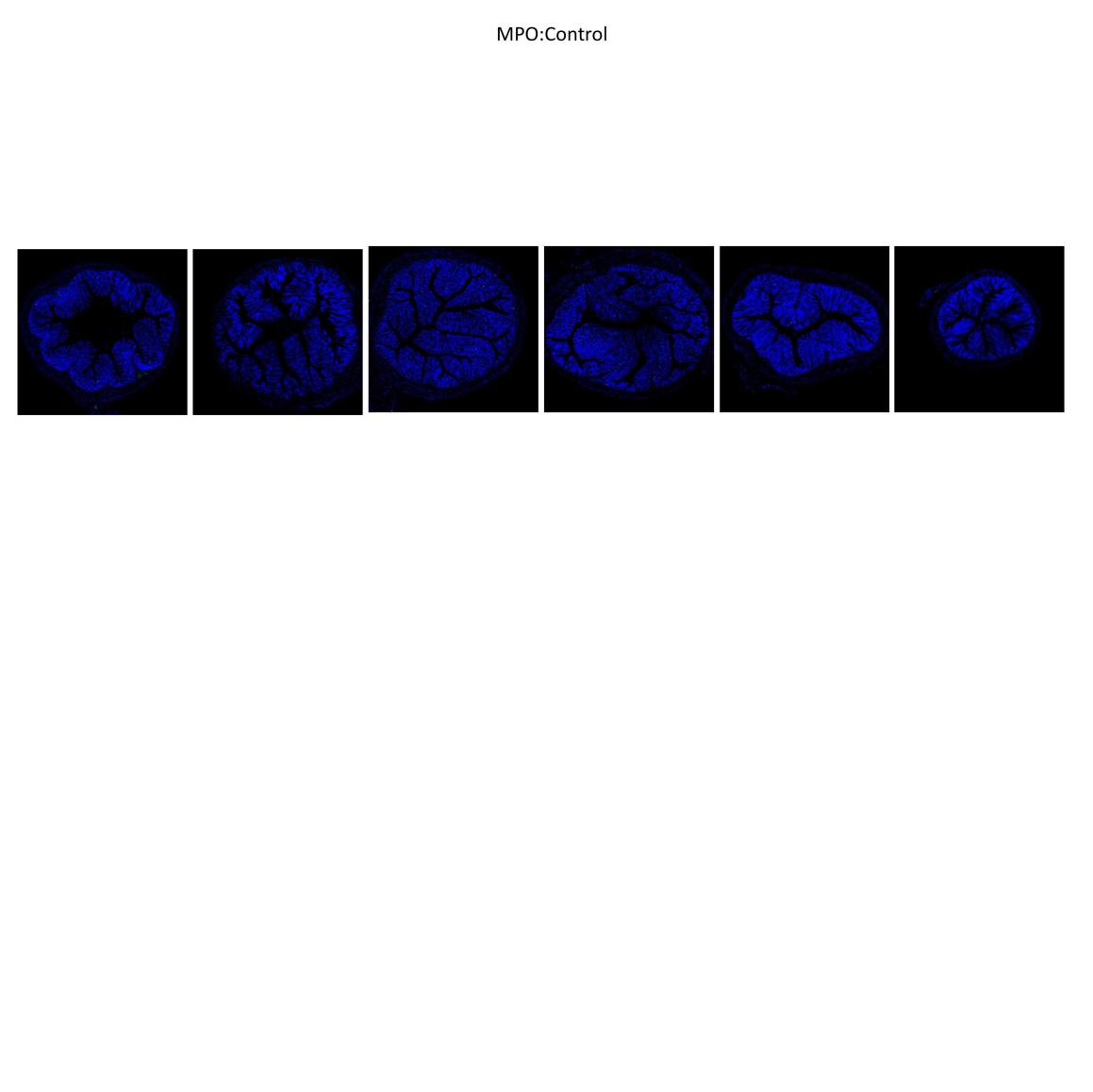


DSS-MPO :


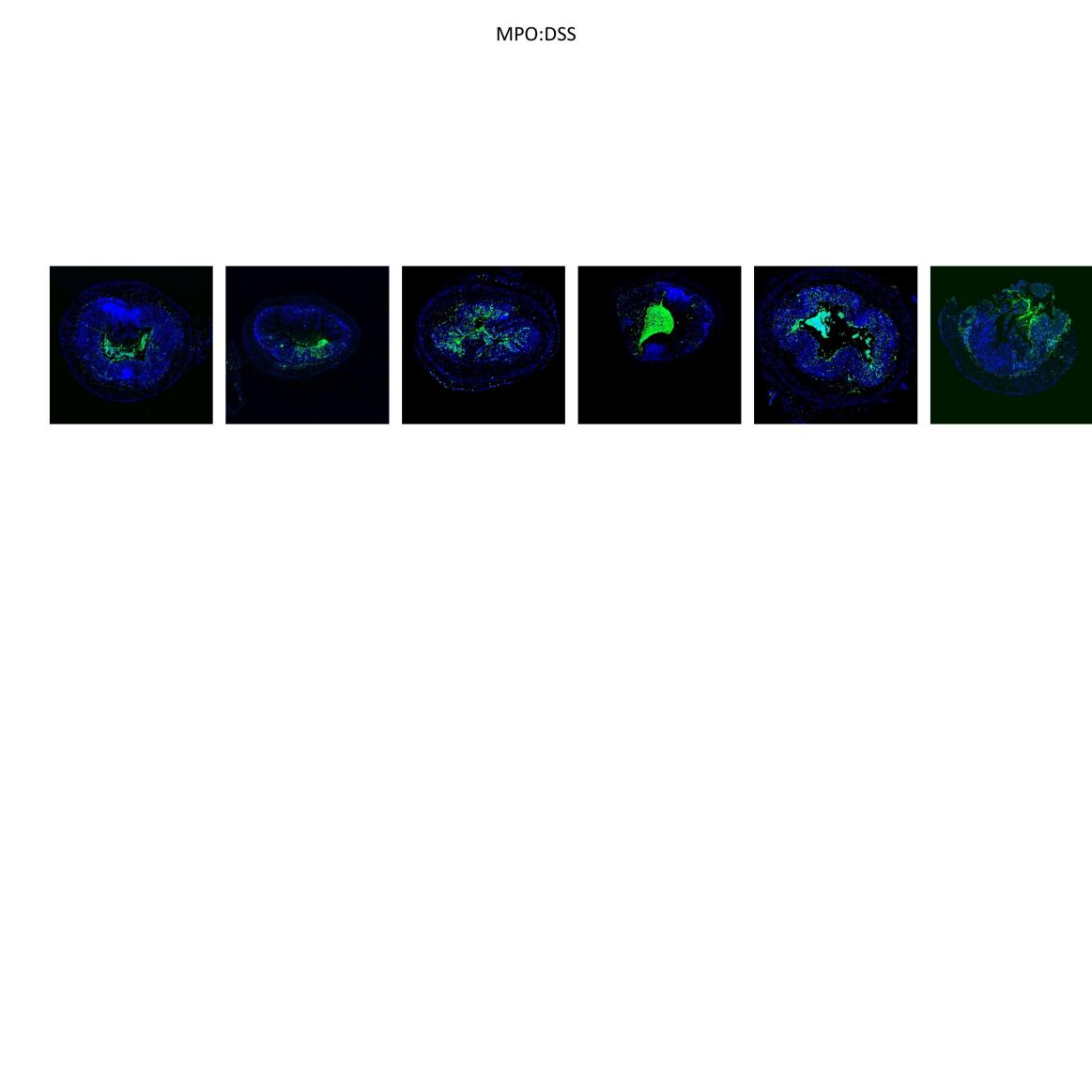


DSS+HNVs-L-MPO :


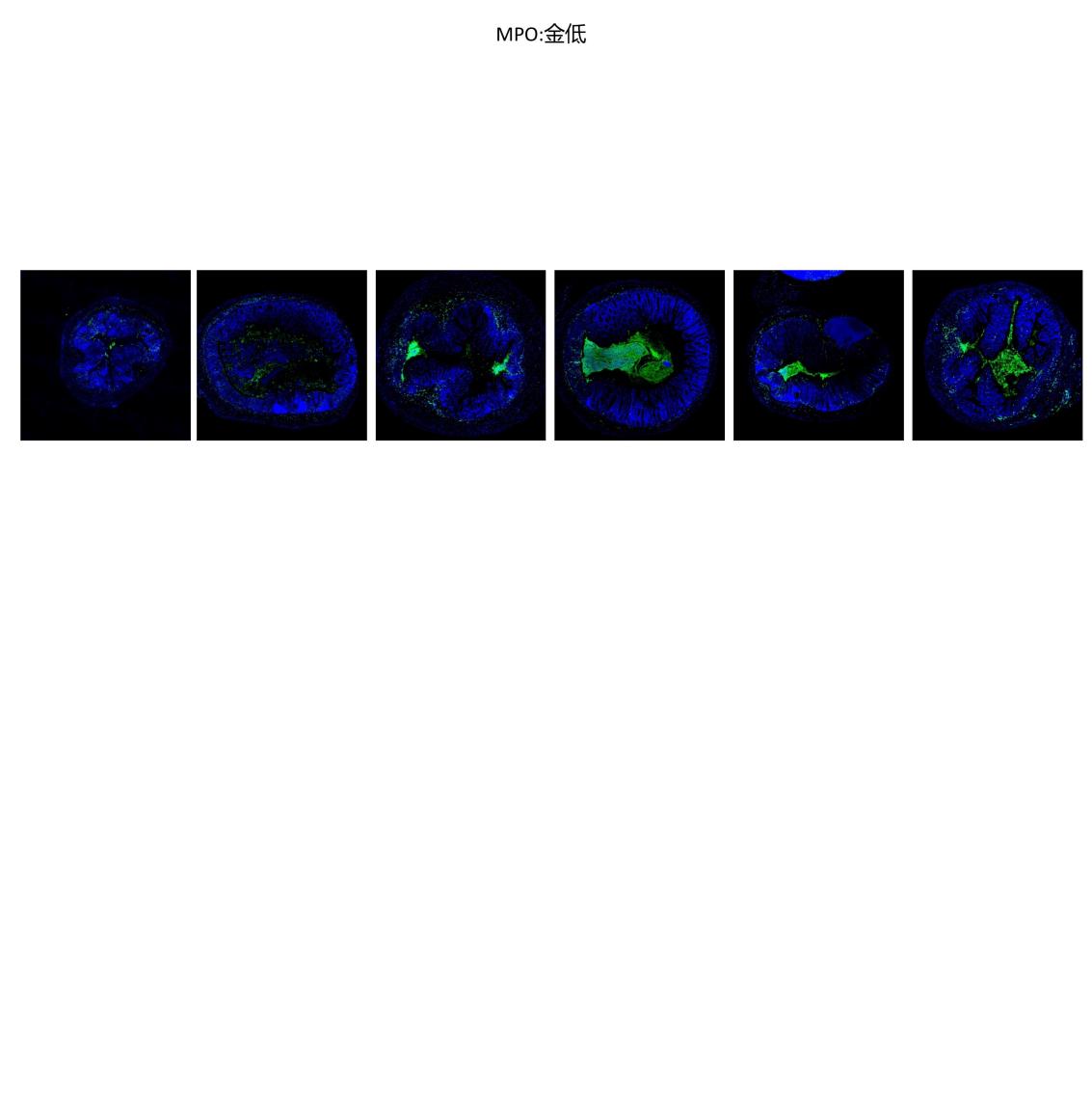


DSS+HNVs-H-MPO :


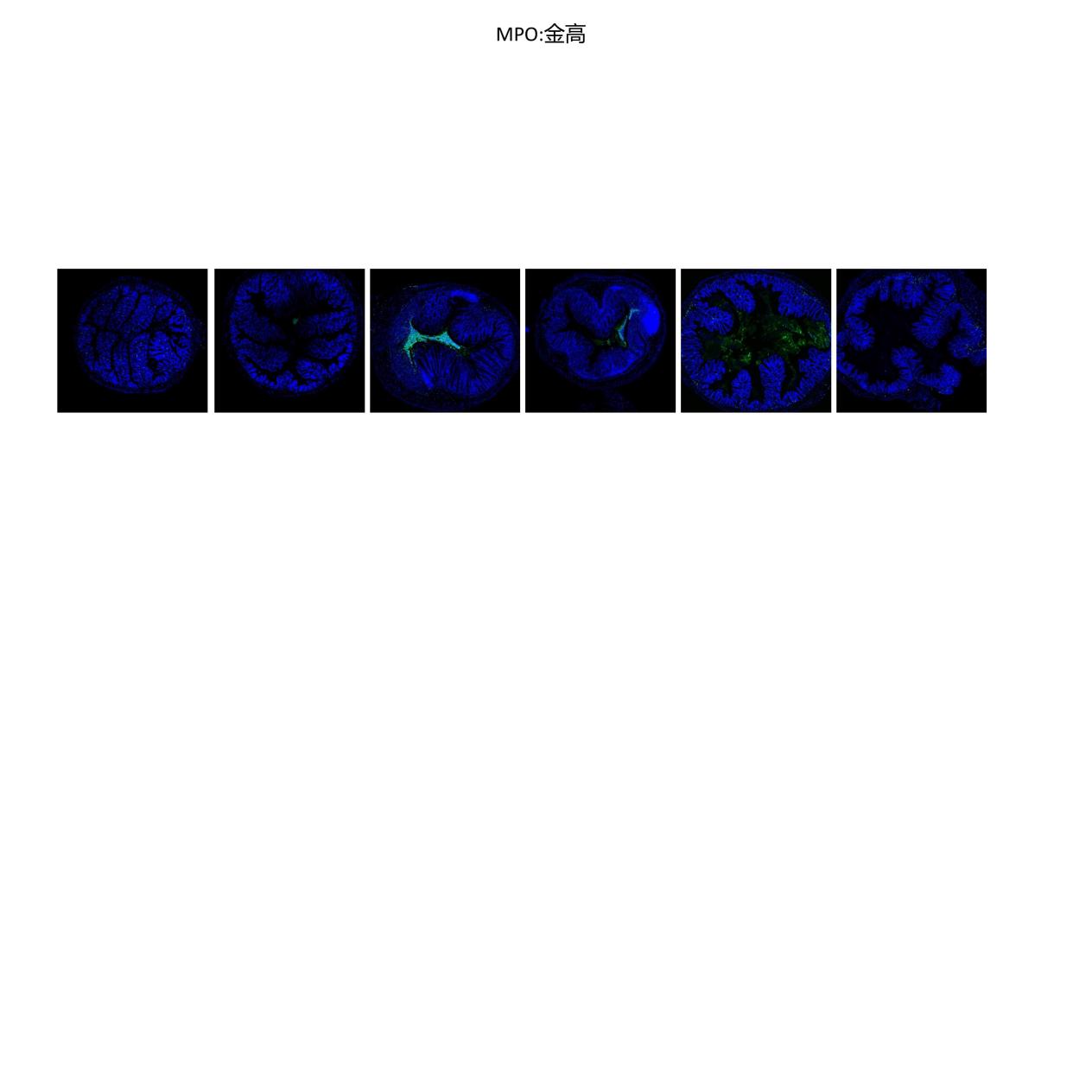


DSS+5-ASA-MPO :


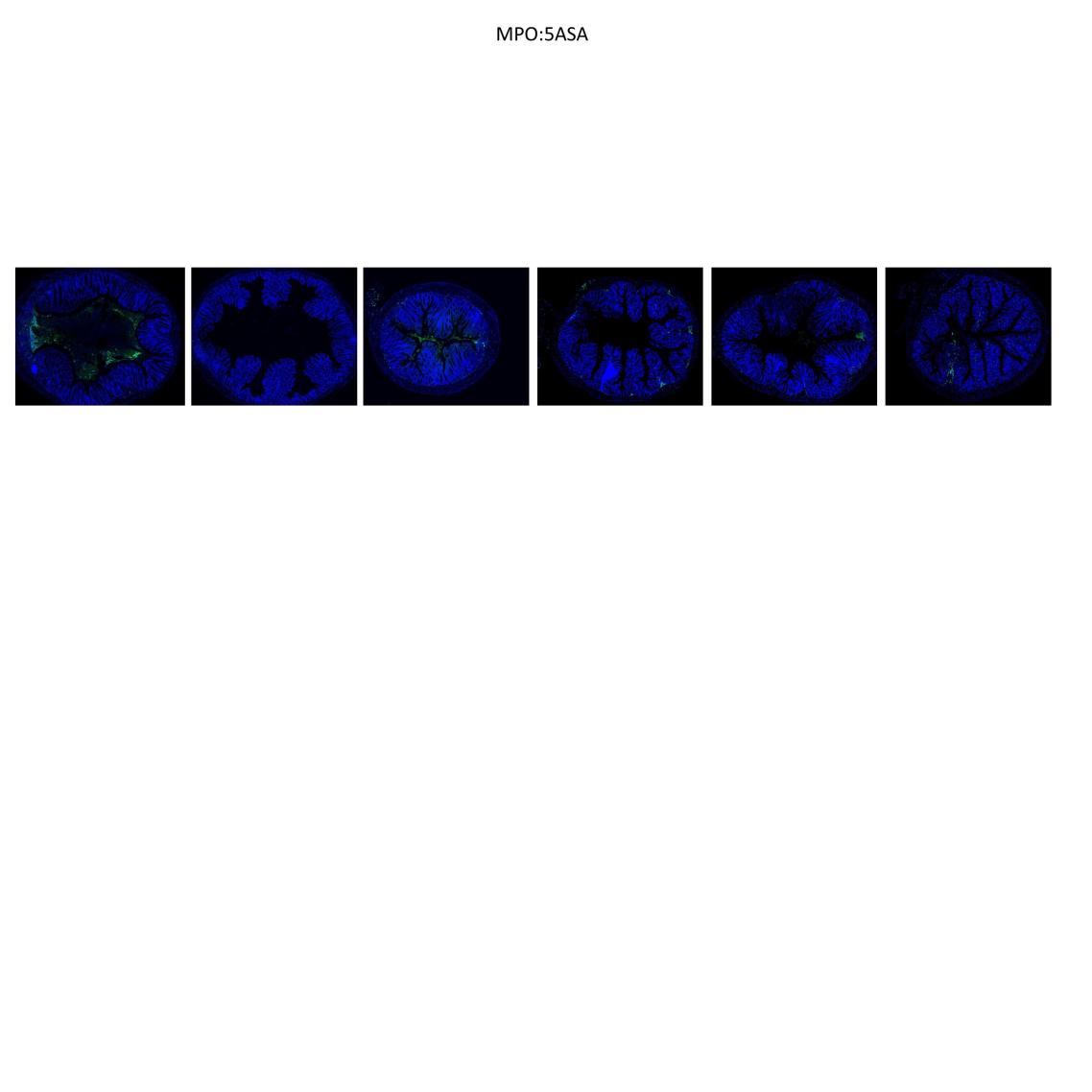


Figure 4A

Control-Occludin :


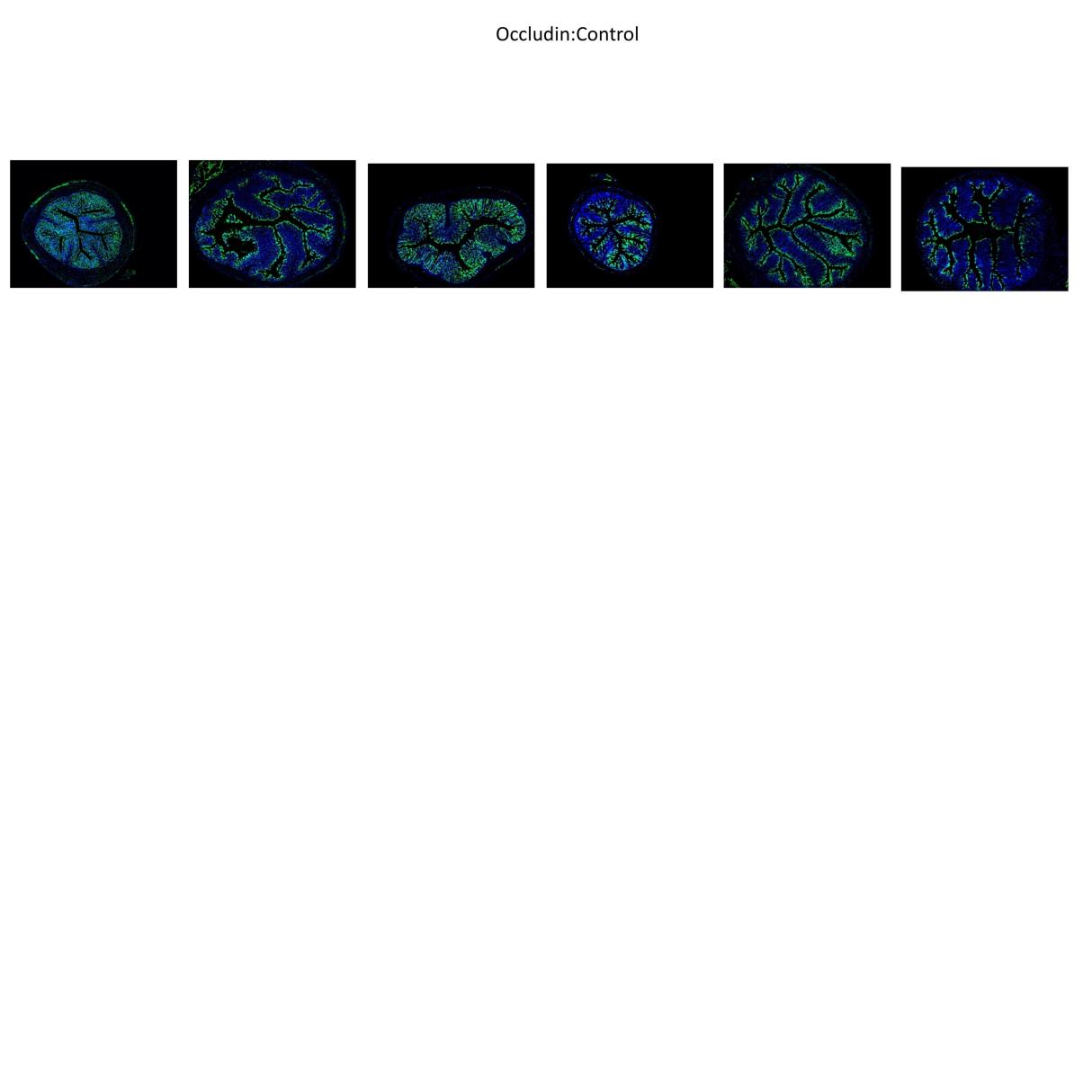


DSS-Occludin :


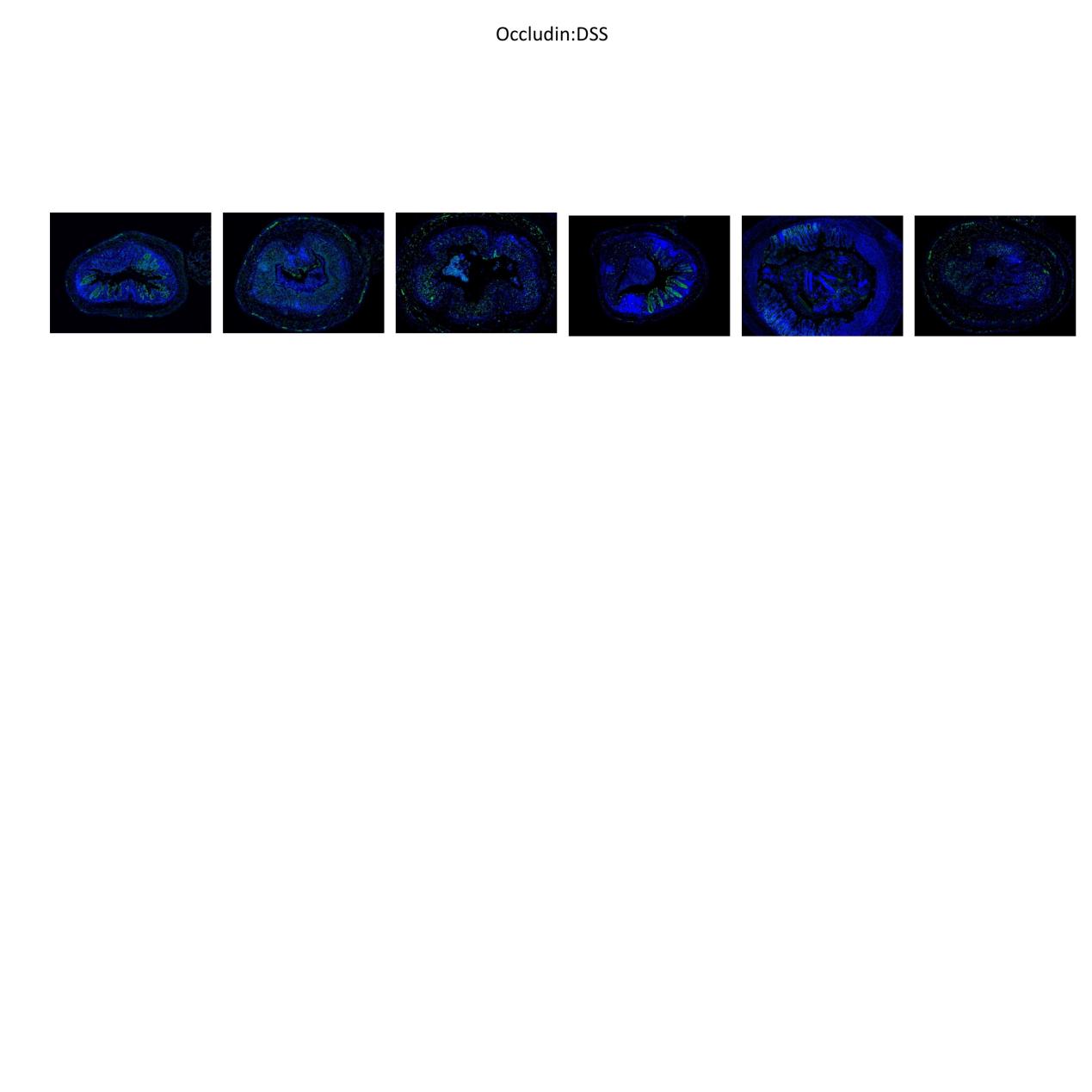


DSS+HNVs-L-Occludin :


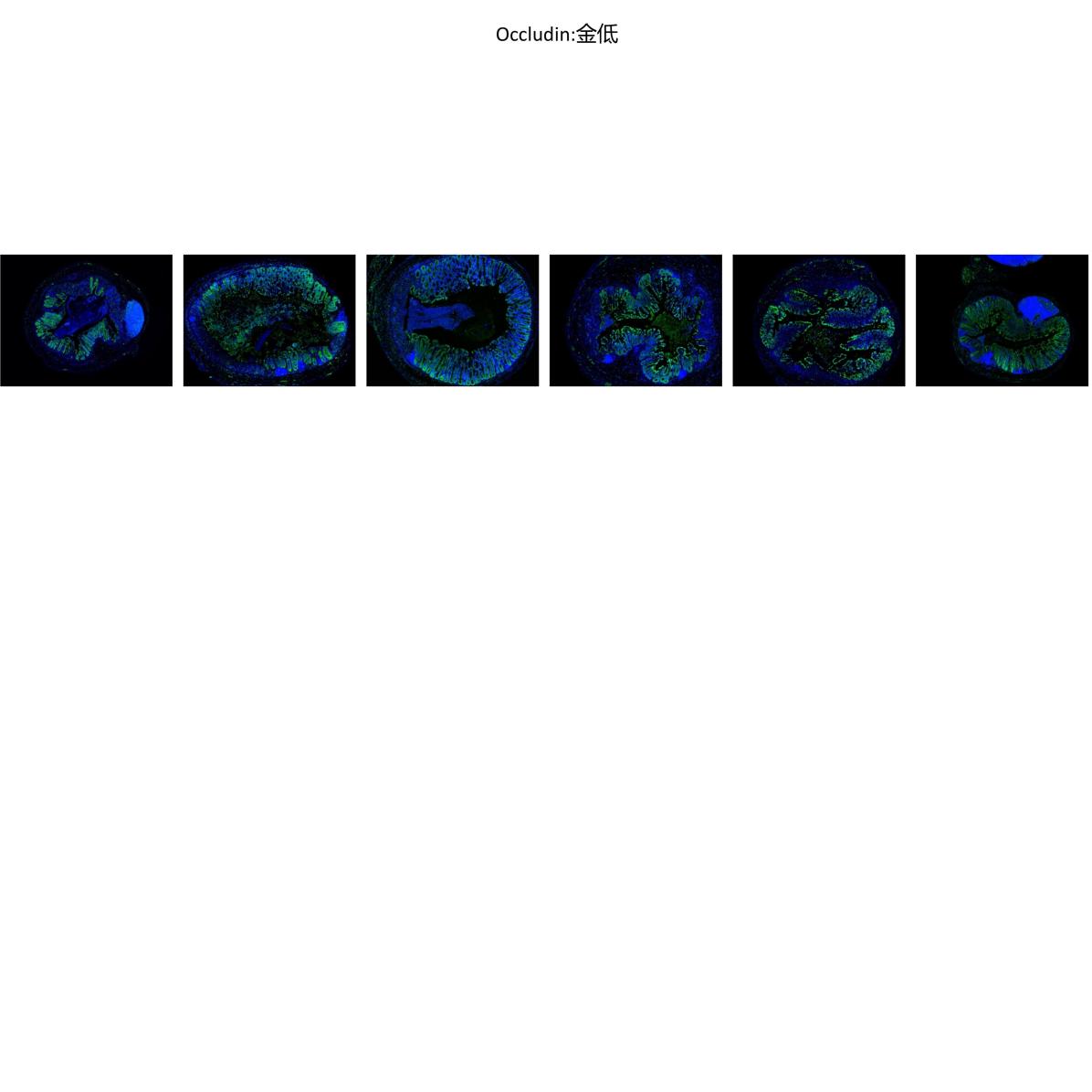


DSS+HNVs-H-Occludin :


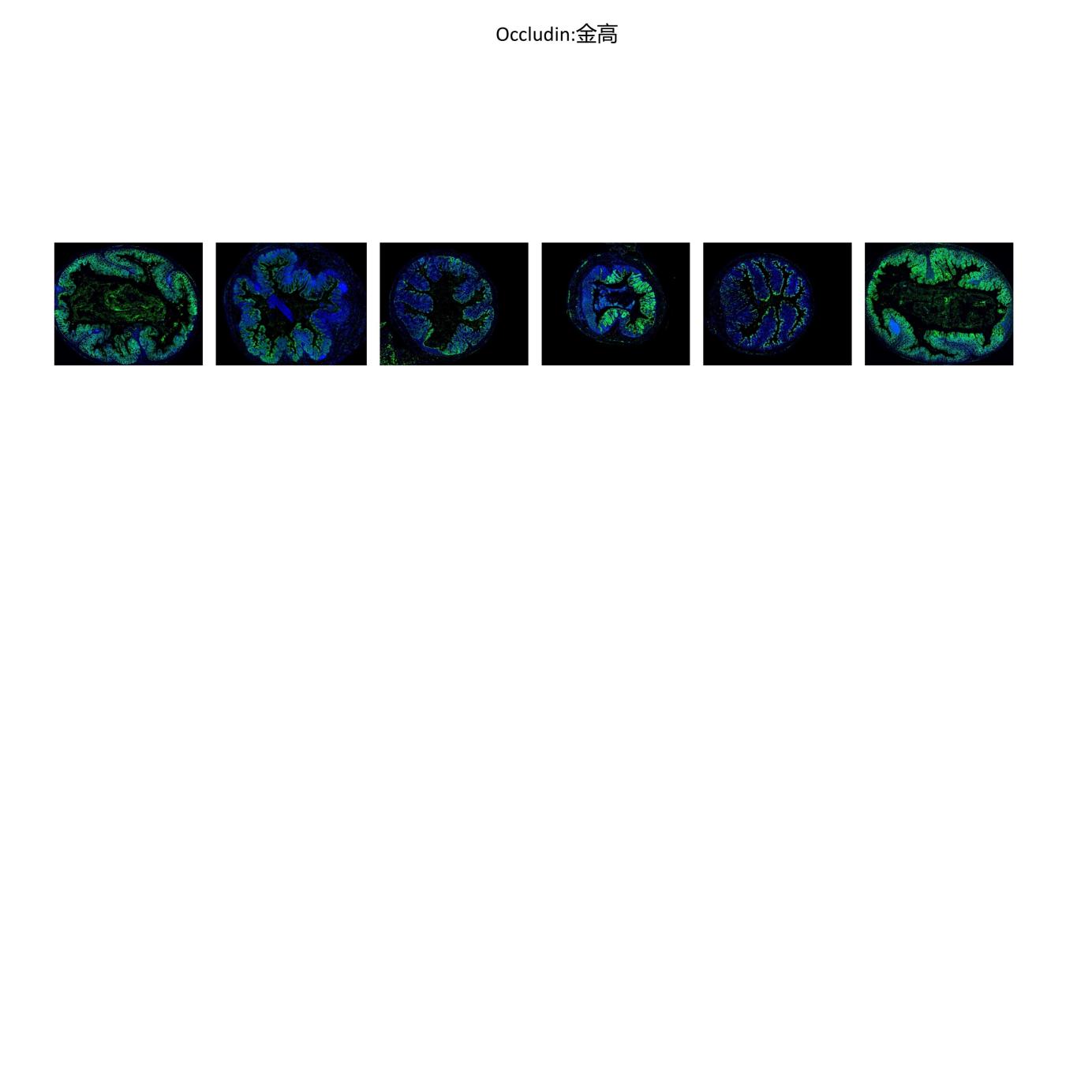


DSS+5-ASA-Occludin :


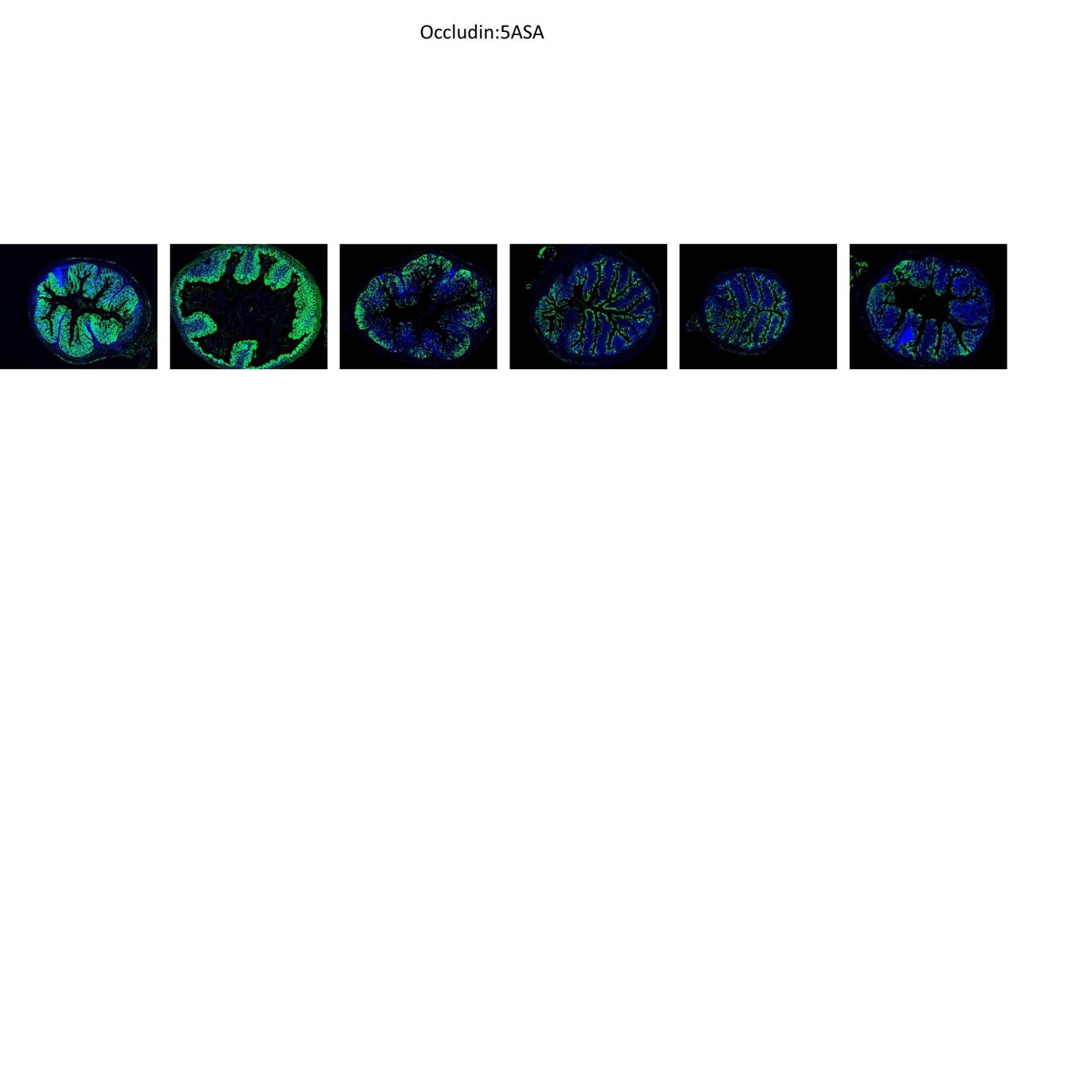


Control-ZO-1 :


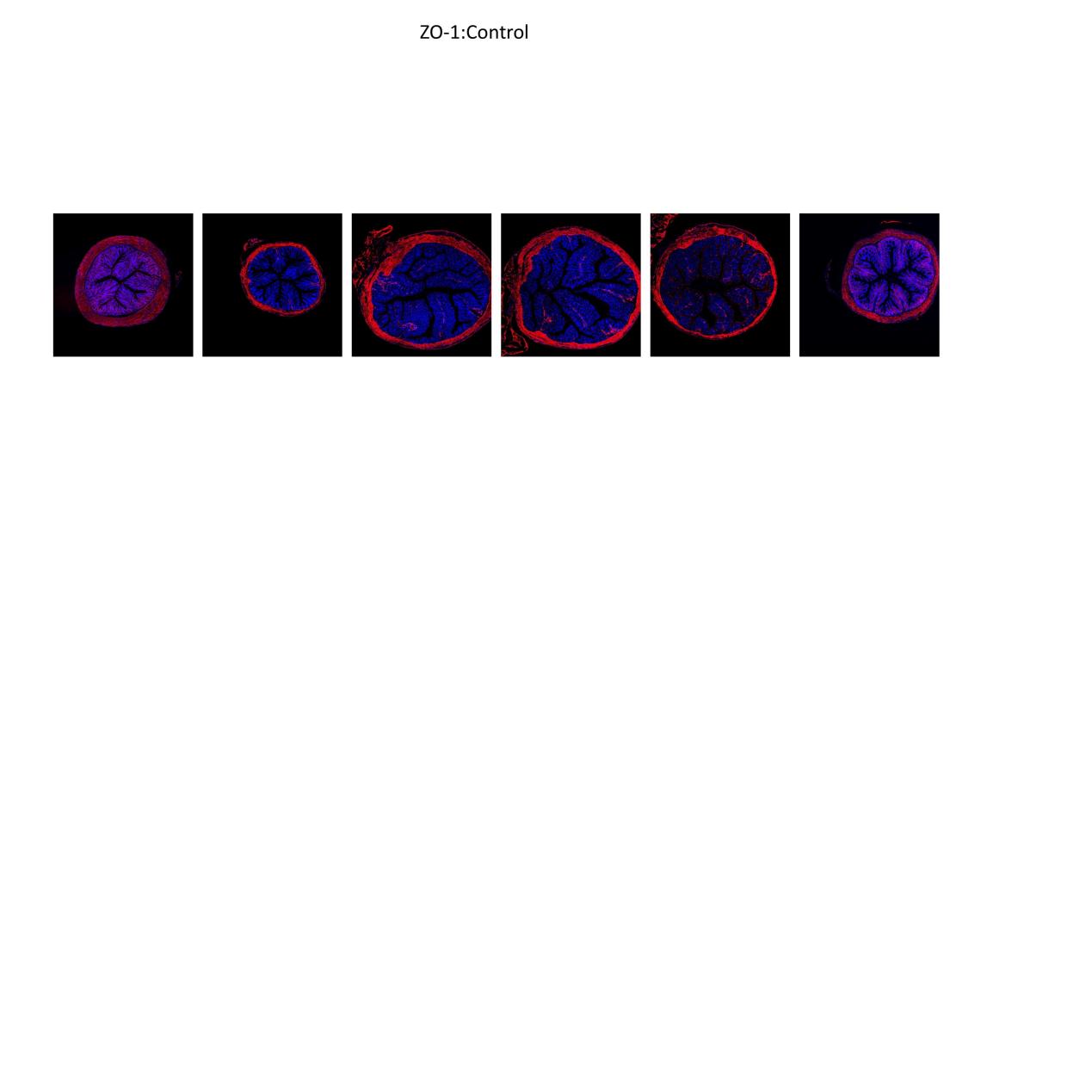


DSS-ZO-1 :


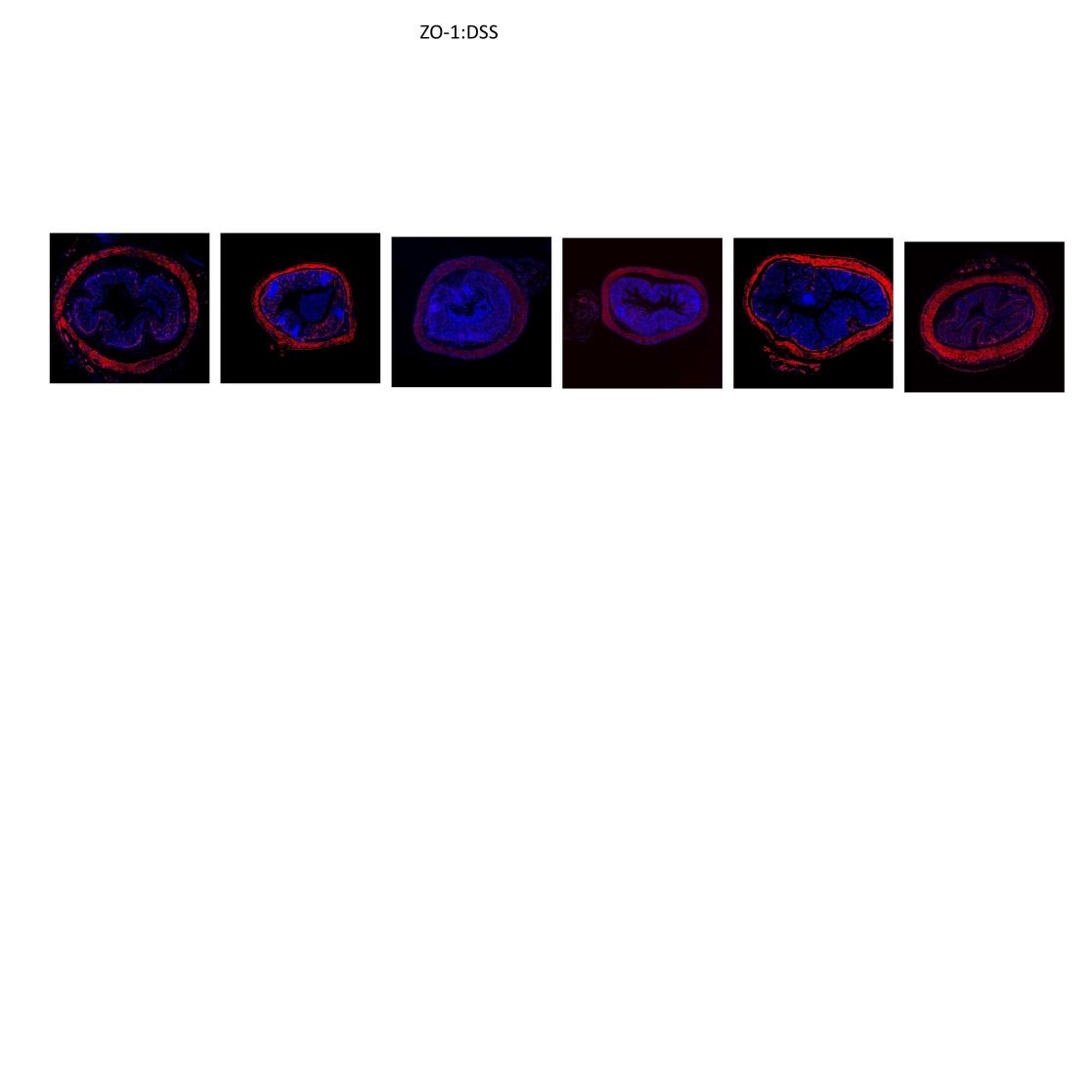


DSS+HNVs-L-ZO-1 :


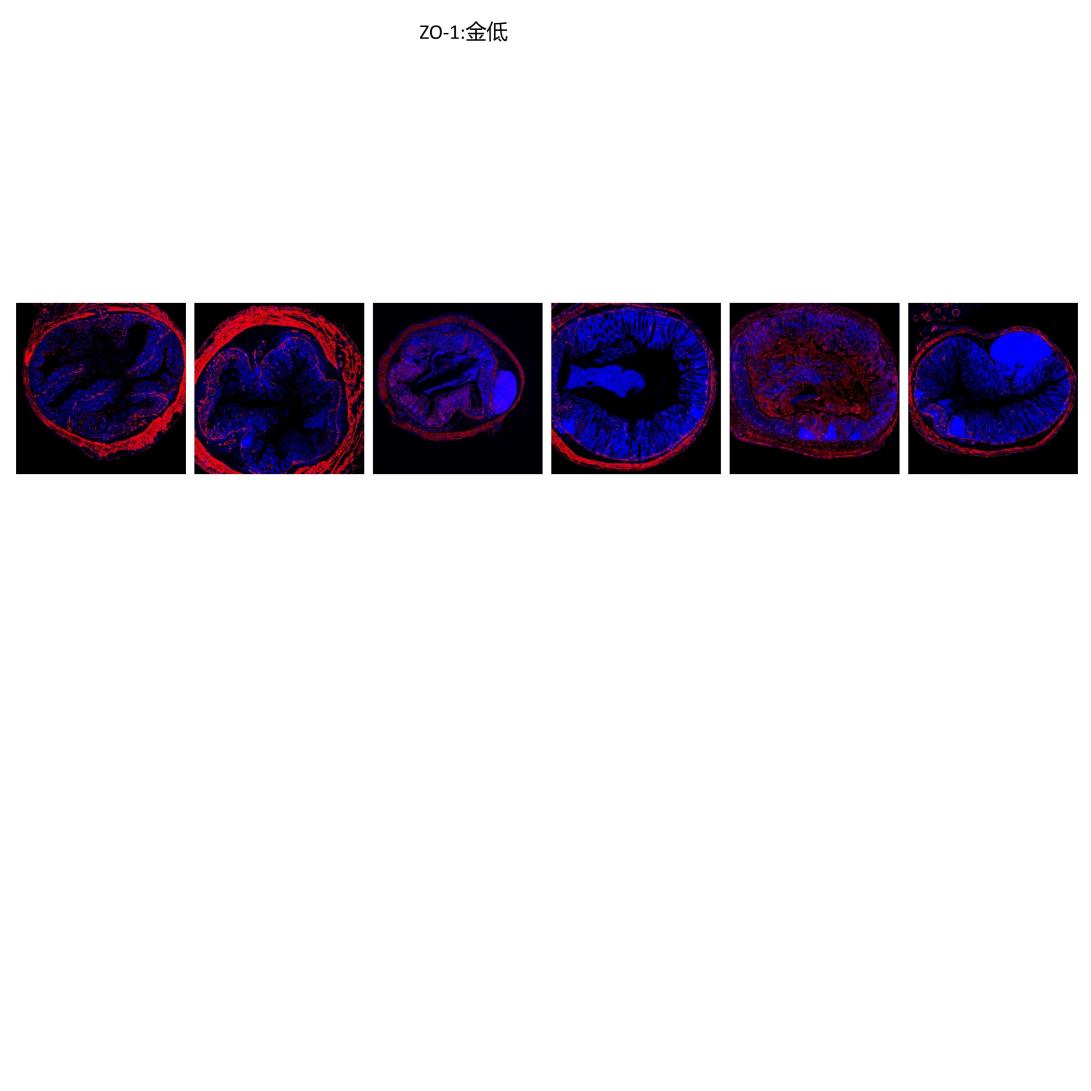


DSS+HNVs-H-ZO-1 :


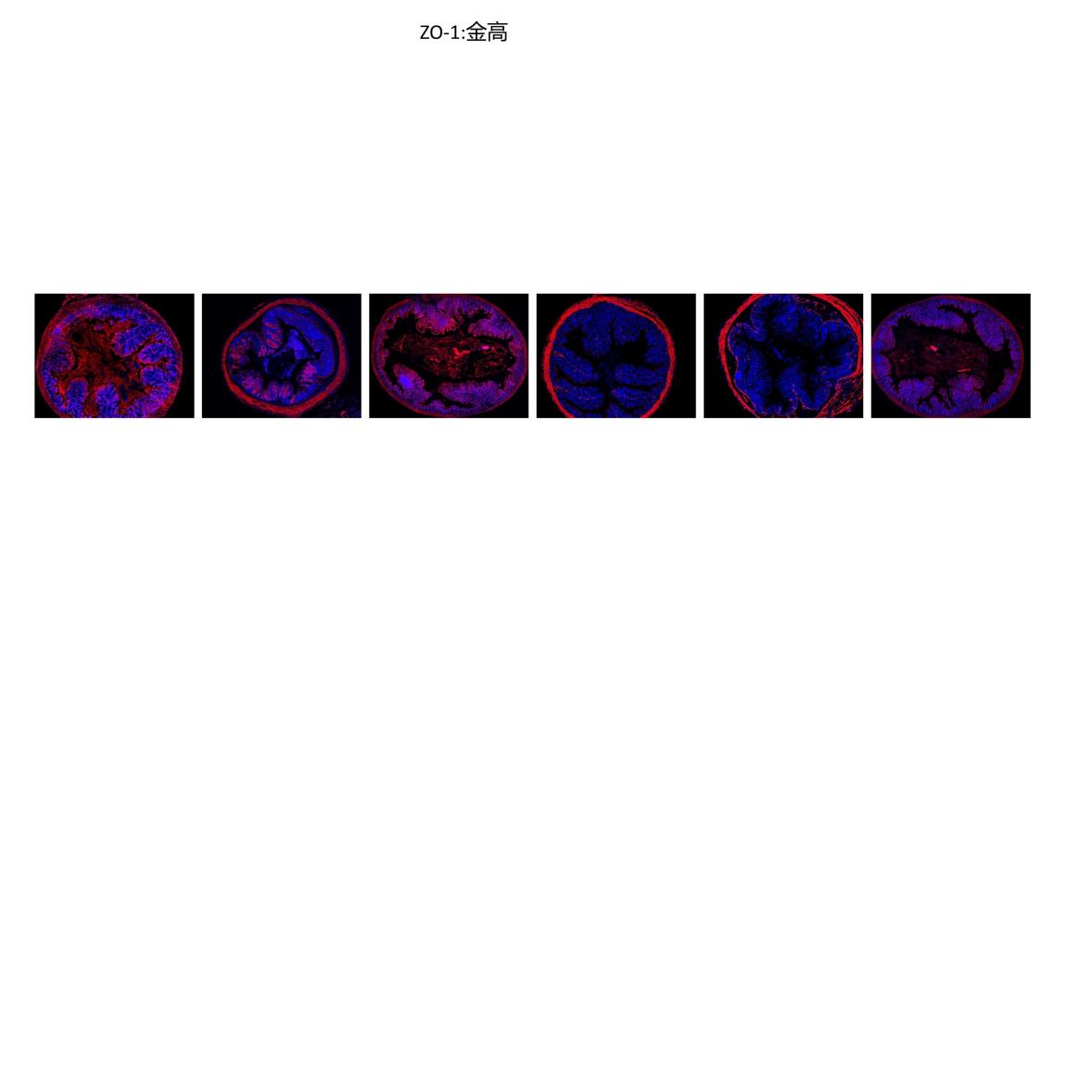


DSS+5-ASA-ZO-1 :


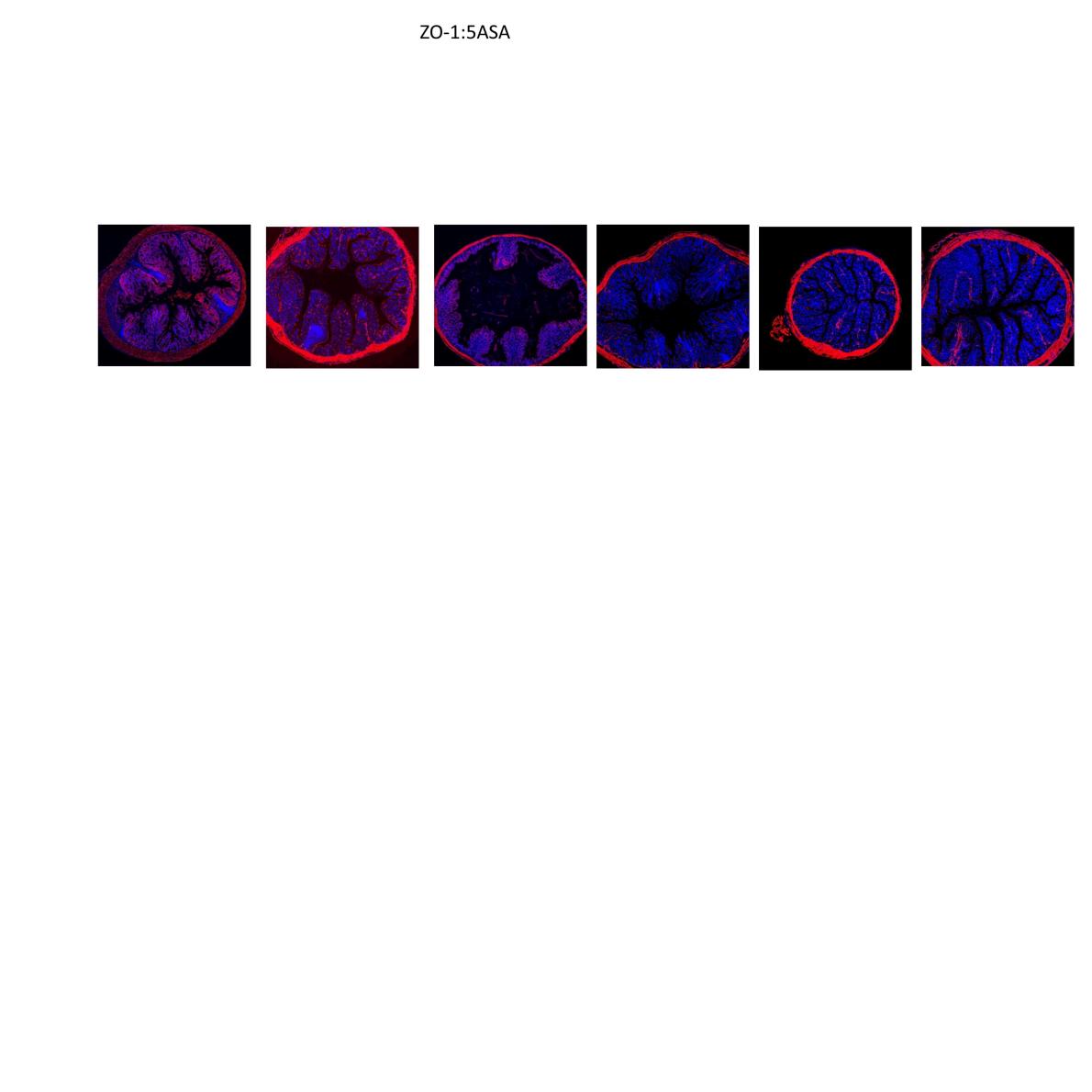


Control-Muc-2 :


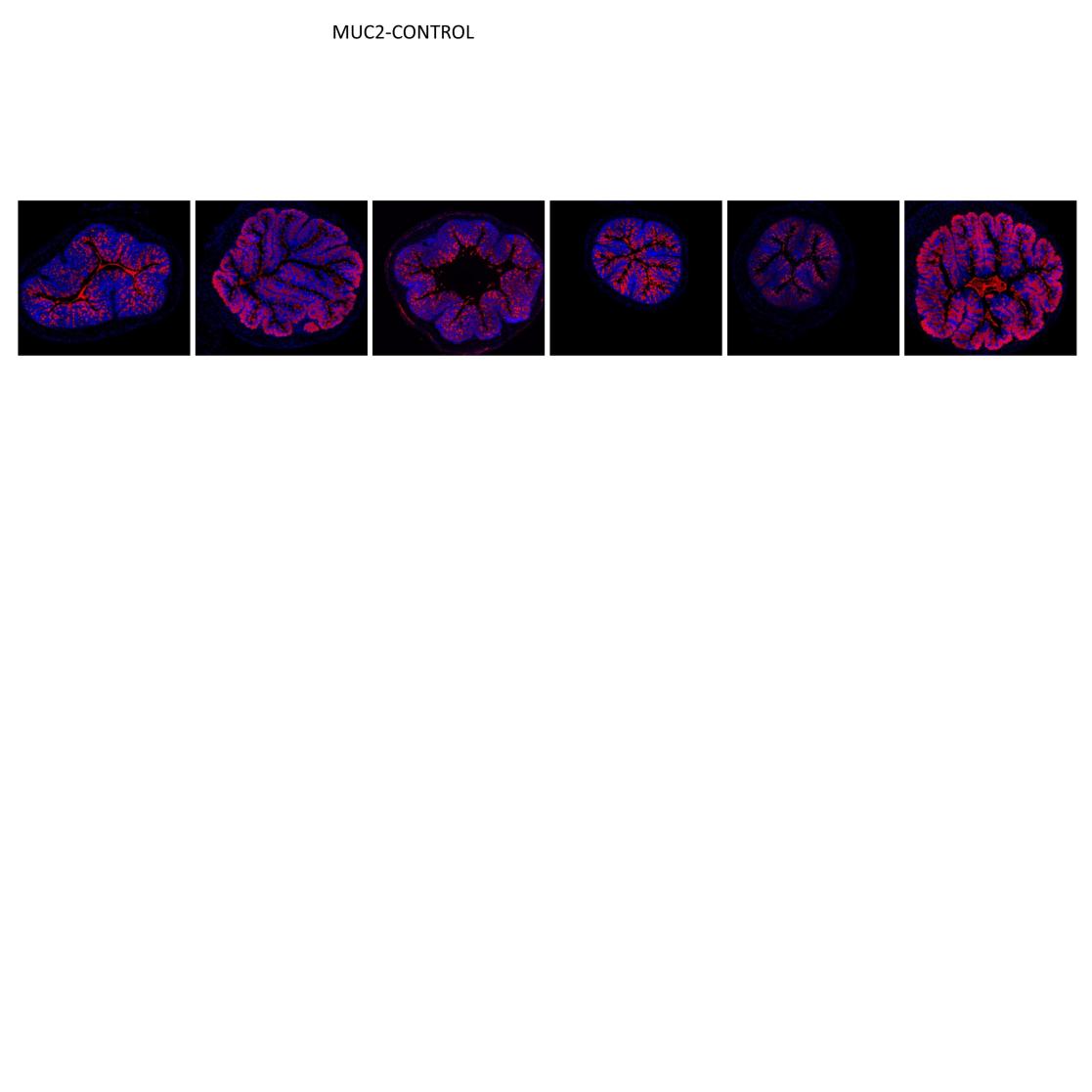


DSS-Muc-2 :


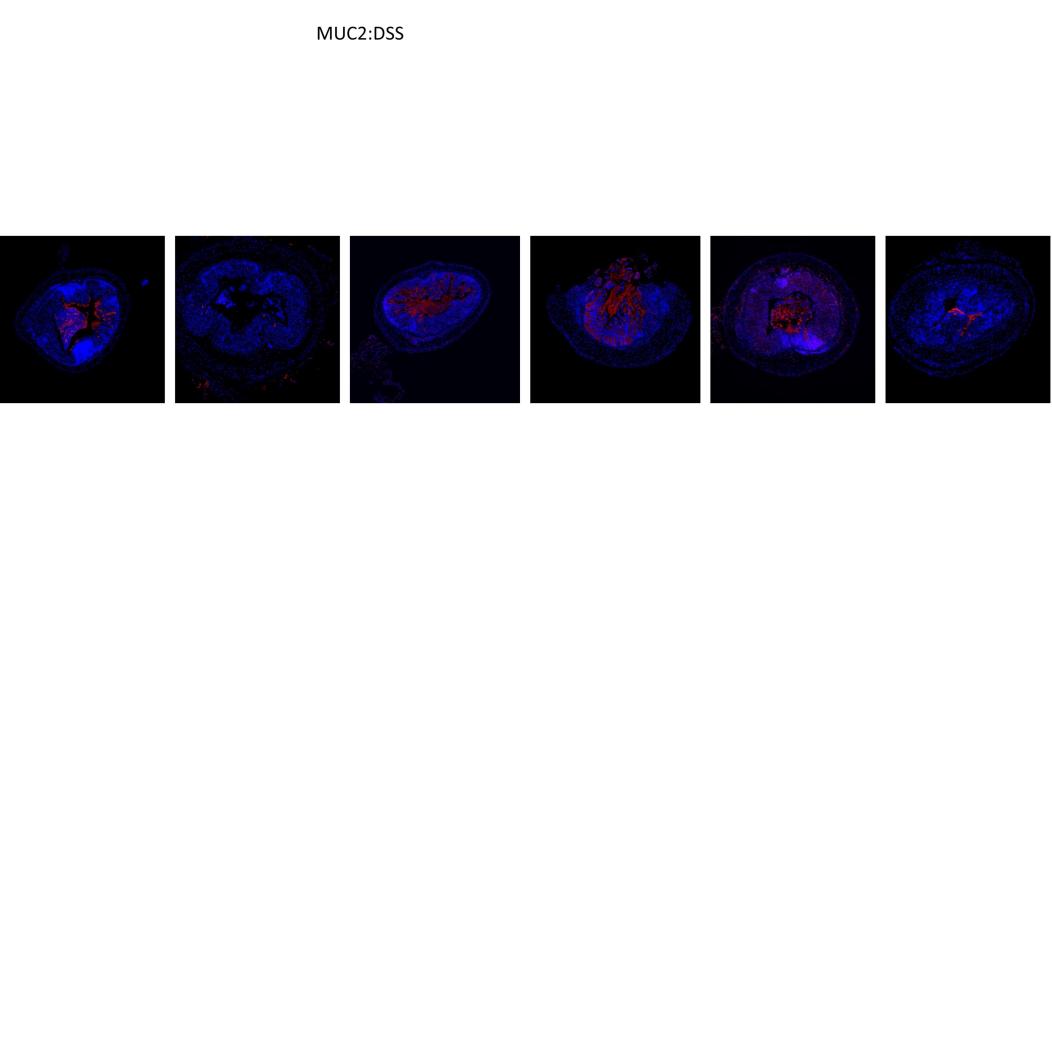


DSS+HNVs-L-Muc-2 :


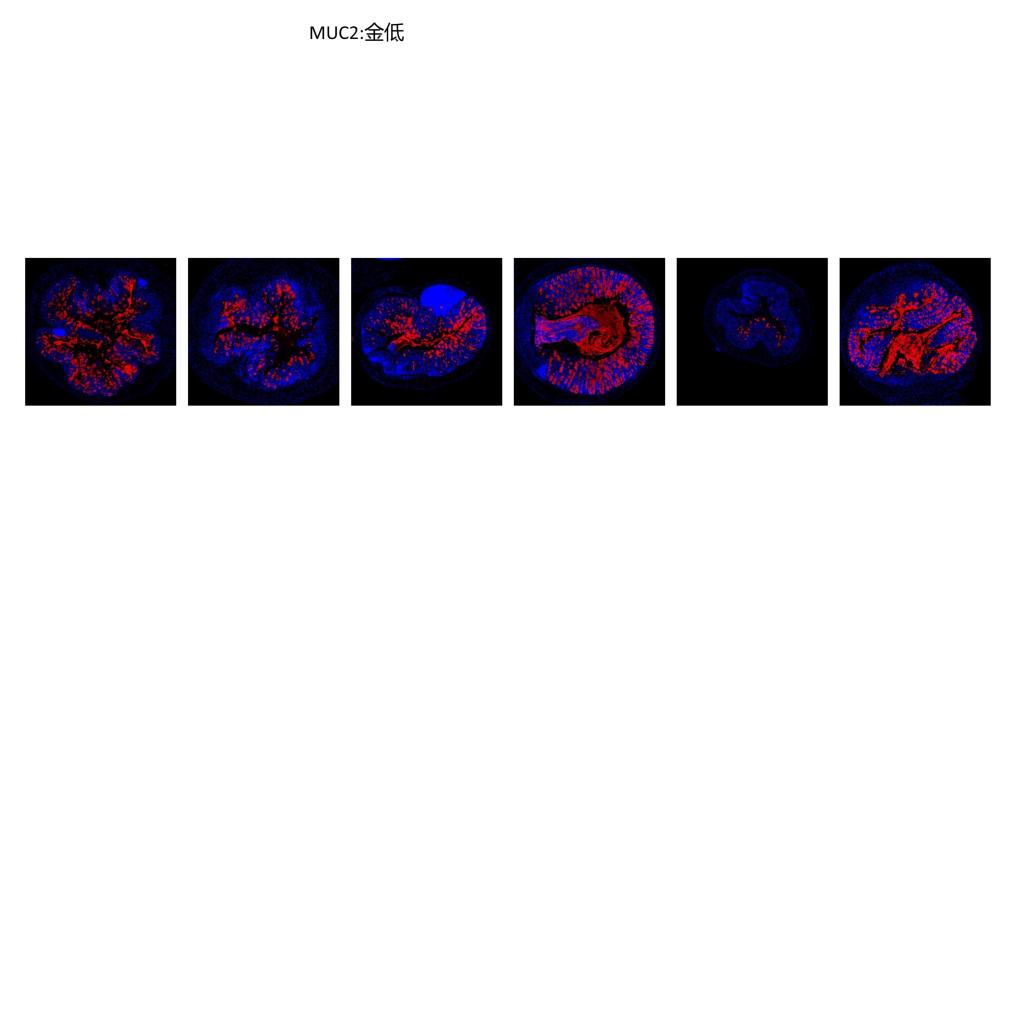


DSS+HNVs-H-Muc-2 :


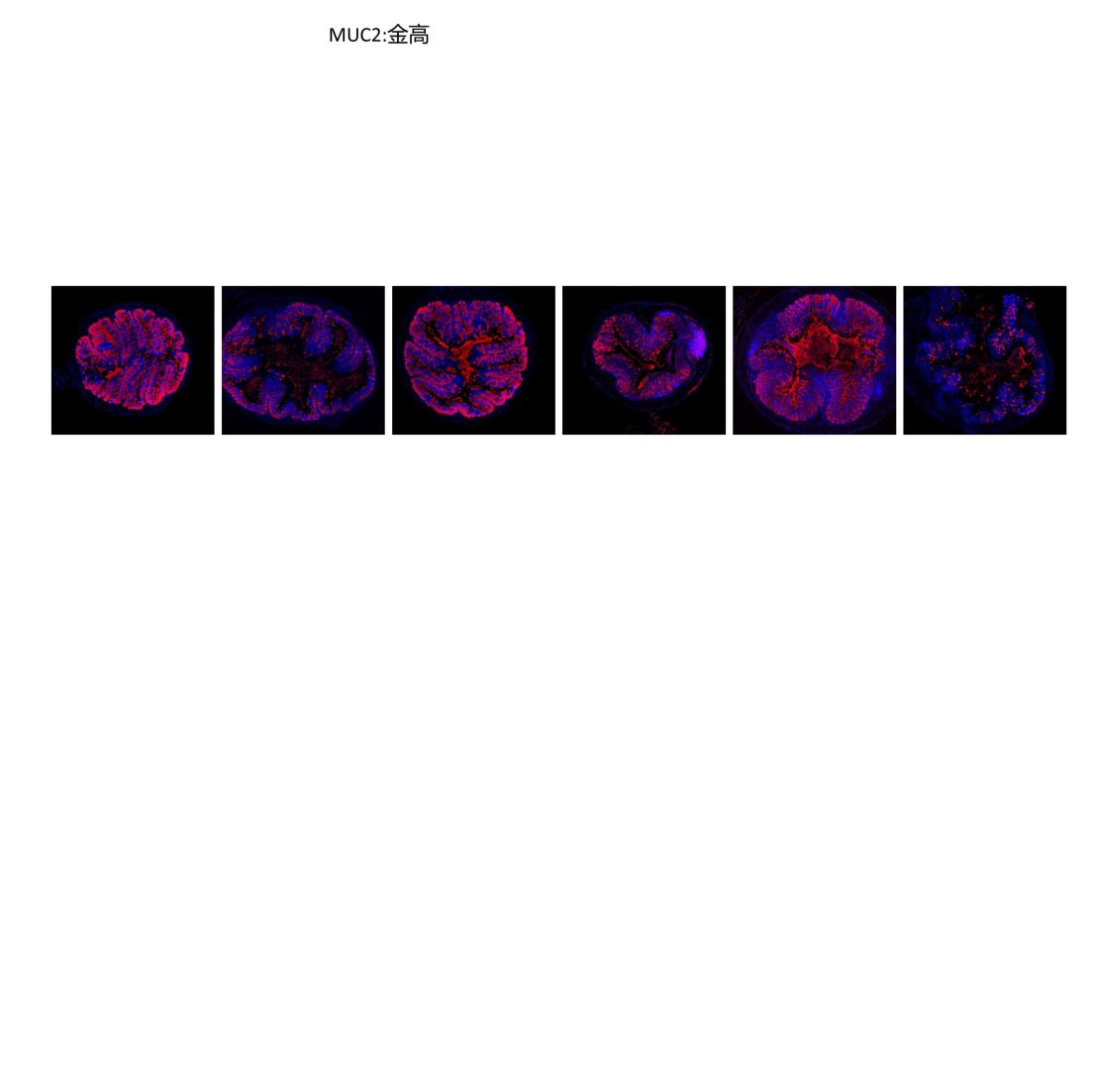


DSS+5-ASA-Muc-2 :


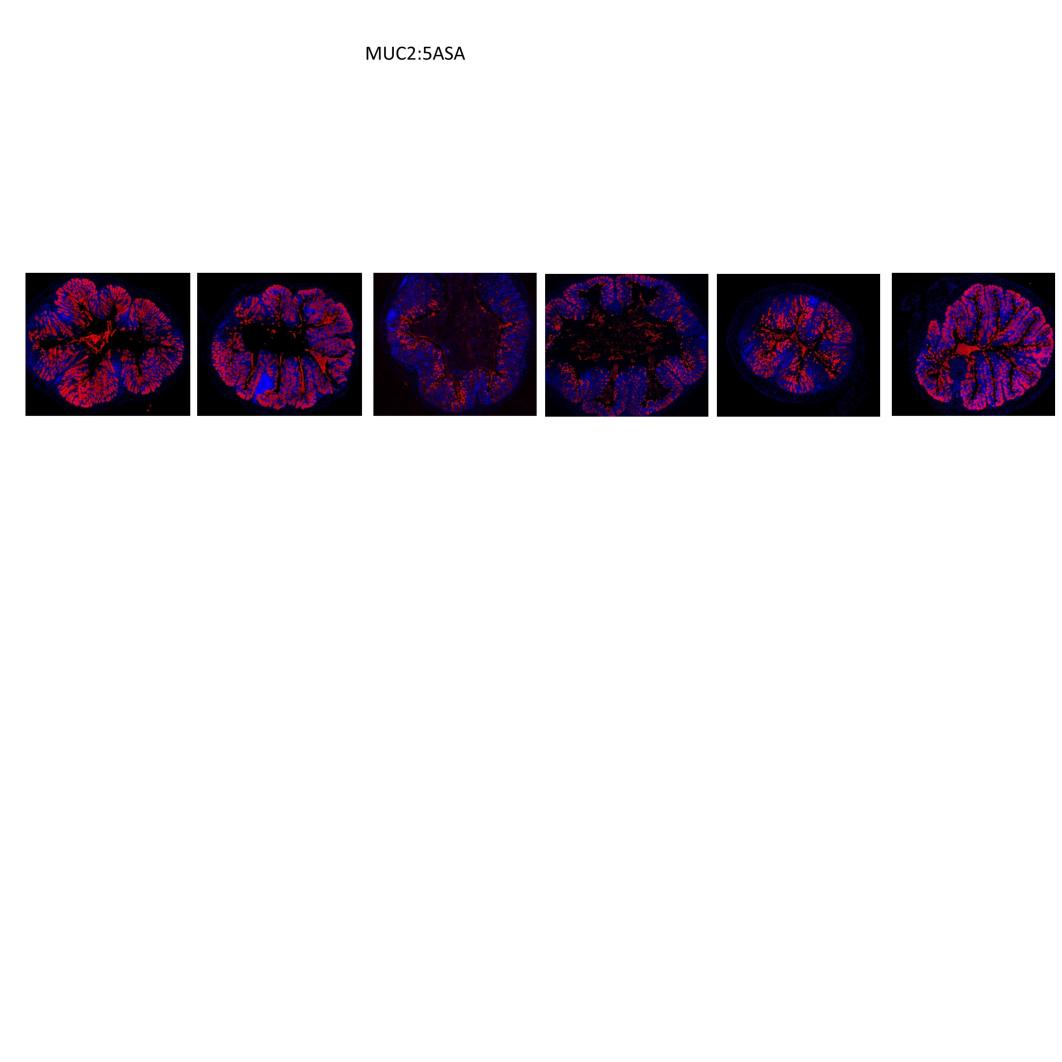


Control：Goblet cells：


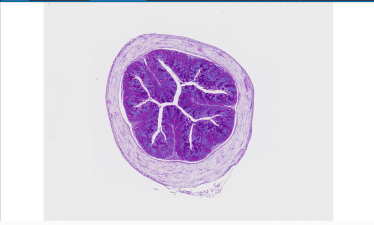

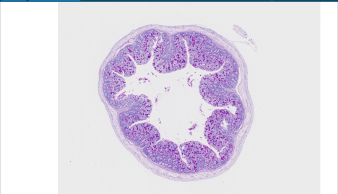

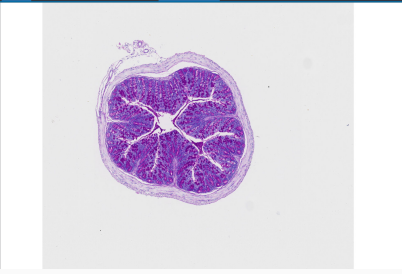

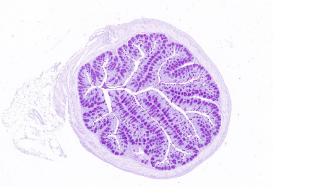

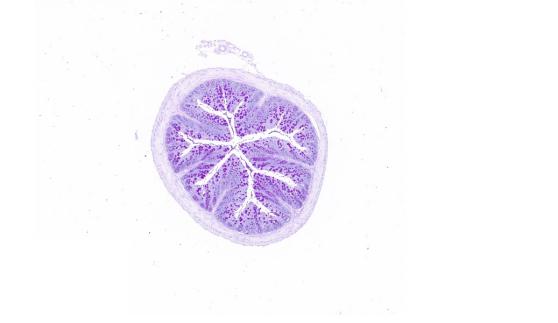

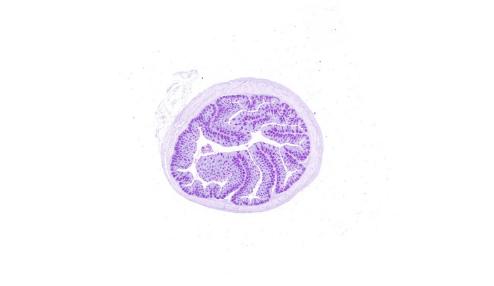


DSS：Goblet cells：


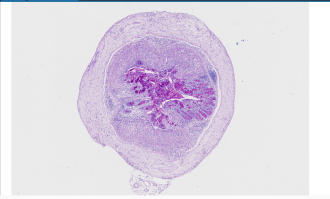

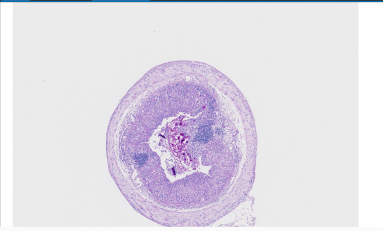

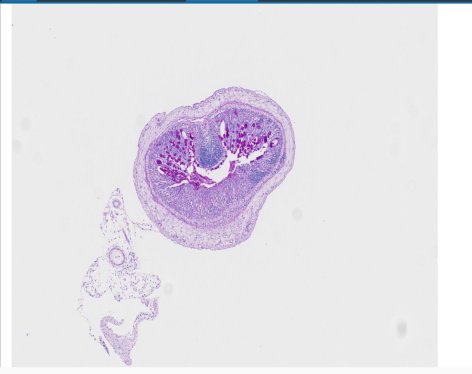

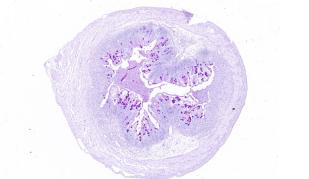

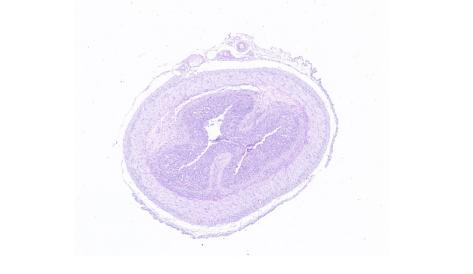

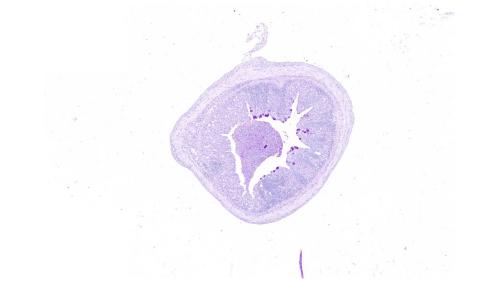


DSS+HNVs-L：Goblet cells：


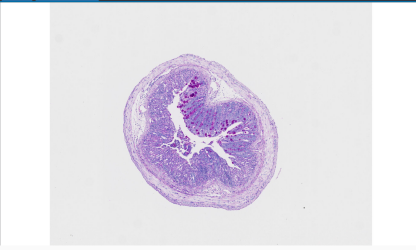

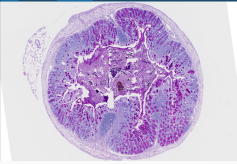

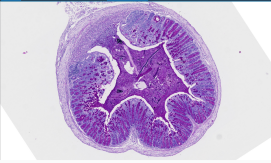

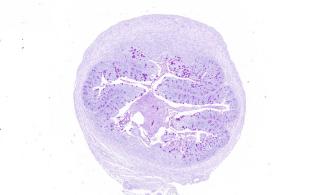

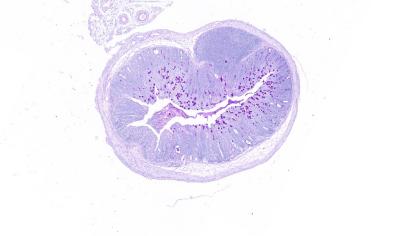

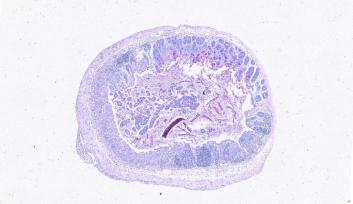


DSS+HNVs-H：Goblet cells：


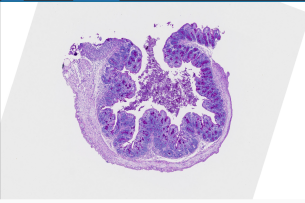

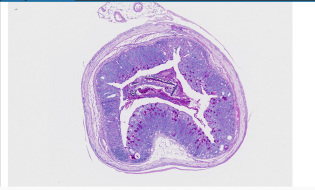

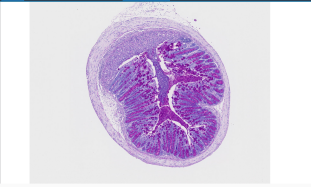

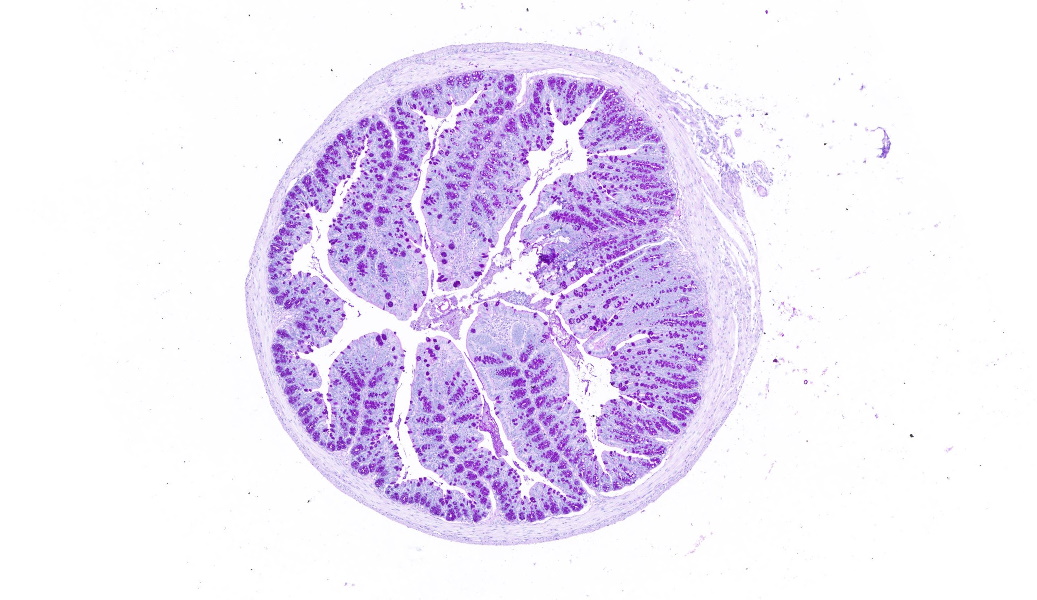

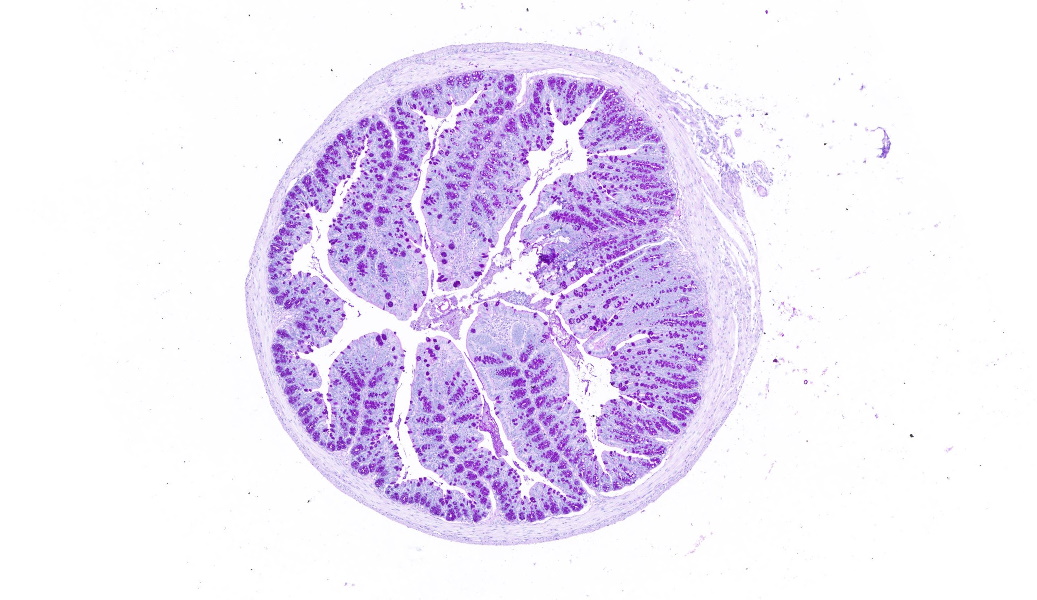


DSS+5-ASA：Goblet cells：

Control-Ki67：

DSS-Ki67：

DSS+HNVs-L-Ki67：

DSS+HNVs-H-Ki67：

DSS+5-ASA-Ki67：

Figure 8A

Control-Foxp3：

DSS-Foxp3：

DSS+HNVs-L-Foxp3：

DSS+HNVs-H-Foxp3：

DSS+5-ASA-Foxp3：

Control-IL-17：

DSS-IL-17：

DSS+HNVs-L-IL-17：

DSS+HNVs-H-IL-17：

DSS+5-ASA-IL-17：

Abx-Control：

Abx-DSS：

Abx-DSS+HNVs：

FMT-Control：

FMT-DSS:

FMT-DSS+HNVs:

FMT-DSS+mHNVs:
